# Exosome-Derived From Sepsis Patients' Blood Promoted Pyroptosis of Cardiomyocytes by Regulating miR-885-5p/HMBOX1

**DOI:** 10.3389/fcvm.2022.774193

**Published:** 2022-03-08

**Authors:** Guo-wei Tu, Jie-fei Ma, Jia-kun Li, Ying Su, Jing-chao Luo, Guang-wei Hao, Ming-hao Luo, Yi-rui Cao, Yi Zhang, Zhe Luo

**Affiliations:** ^1^Department of Critical Care Medicine, Zhongshan Hospital, Fudan University, Shanghai, China; ^2^Department of Critical Care Medicine, Xiamen Branch, Zhongshan Hospital, Fudan University, Xiamen, China; ^3^Shanghai Medical College, Fudan University, Shanghai, China; ^4^Shanghai Key Laboratory of Organ Transplantation, Shanghai, China; ^5^Biomedical Research Center, Institute for Clinical Sciences, Zhongshan Hospital, Fudan University, Shanghai, China; ^6^Shanghai Key Laboratory of Lung Inflammation and Injury, Shanghai, China

**Keywords:** sepsis, exosomes, pyroptosis, cardiomyocytes, microRNA

## Abstract

**Background:**

Septic myocardial depression has been associated with increased morbidity and mortality. miR-885-5p has been shown to regulate cell growth, senescence, and/or apoptosis. Published studies demonstrated that Homeobox-containing protein 1 (HMBOX1) inhibits inflammatory response, regulates cell autophagy, and apoptosis. However, the role of miR-885-5p/HMBOX1 in sepsis and septic myocardial depression and the underlying mechanism is not fully understood.

**Materials and Methods:**

Exosomes (exos) derived from sepsis patients (sepsis-exos) were isolated using ultracentrifugation. Rats were subjected to cecal ligation and puncture surgery and treated with sepsis-exos. HMBOX1 was knocked down or overexpressed in AC16 cells using lentiviral plasmids carrying short interfering RNAs targeting human HMBOX1 or carrying HMBOX1 cDNA. Cell pyroptosis was measured by flow cytometry. The secretion of IL-1β and IL-18 was examined by ELISA kits. Quantitative polymerase chain reaction (PCR) or western blot was used for gene expression.

**Results:**

Sepsis-exos increased the level of miR-885-5p, decreased HMBOX1, elevated IL-1β and IL-18, and promoted pyroptosis in AC16 cells. Septic rats treated with sepsis-exos increased the serum inflammatory cytokines is associated with increased pyroptosis-related proteins of hearts. MiR-885-5p bound to the three prime untranslated regions of HMBOX1 to negatively regulate its expression. Overexpressing HMBOX1 reversed miR-885-5p-induced elevation of inflammatory cytokines and upregulation of NLRP3, caspase-1, and GSDMD-N in AC16 cells. The mechanistic study indicated that the effect of HMBOX1 was NF-κB dependent.

**Conclusion:**

Sepsis-exos promoted the pyroptosis of AC16 cells through miR-885-5p *via* HMBOX1. The results show the significance of the miR-885-5p/HMBOX1 axis in myocardial cell pyroptosis and provide new directions for the treatment of septic myocardial depression.

## Introduction

Sepsis is a serious inflammatory disease caused by the systemic immune response to infections (1). About 15% of sepsis patients undergo septic shock, leading to 10% admissions to intensive care units and a 50% death rate (2). It has been estimated that 15% of sepsis-related mortality is secondary to myocardial depression (3). The best treatment for myocardial dysfunction is sepsis management. Although myocardial depression in sepsis has been the focus of many investigations, its etiology and molecular mechanism remain unclear.

Exosomes (exos) play an important role in intercellular communication (4). Exos can deliver various bioactive substances to target cells to regulate different biological processes (5). Wang et al. indicated that miR-223 in exos derived from mesenchymal stem cells inhibited macrophage inflammation in sepsis (6). Upregulated levels of serum exosomal miR-885-5p were found in hepatitis C virus-infected blood donors and pre-eclampsia women (7, 8). Besides, miR-885-5p was reported to be a tumor-suppressive factor in inhibiting cell cycle progression and cell survival (9). Therefore, further clarifying the function of serum exosomal miRNAs especially miR-885-5p in patients with sepsis will help in the understanding of the pathogenesis of sepsis affecting myocardial function.

Cardiomyocytes involve in the contractile function of the heart (10). Sepsis is characterized by overwhelming inflammation followed by immunosuppression (11). Pyroptosis, first reported in 1992, is a form of necrotic and inflammatory programmed cell death mediated by masterminds and inflammatory caspases (12). Pyroptosis could be activated *via* the canonical caspase-1 inflammasome pathway (13). The activated caspase-1 not only elevates inflammatory cytokines but also cleaves gasdermin D (GSDMD) to form membrane pores, leading to pyroptosis (14, 15). Numerous studies have proved the correlation between inflammation and pyroptosis. For example, Zeng et al. have shown that pyroptosis tightly controls the release of inflammatory cytokines, and inflammation (16). It has also been demonstrated that inflammation and subsequent myocardial dysfunction, were dramatically increased in caspase-1^−/−^ mice (17). Therefore, the pyroptosis of myocardial cells may be one of the reasons for myocardial dysfunction caused by sepsis. It has been reported that homeobox-containing protein 1 (HMBOX1) is a key immunosuppressive factor that protects hepatocytes from inflammatory responses by inhibiting the infiltration and activation of macrophages (18). In addition, HMBOX1 is also involved in regulating autophagy and apoptosis of vascular endothelial cells (19). Importantly, data collected from the Targetscan database (http://www.targetscan.org/) indicated that HMBOX1 is a potential target for miR-885-5p. However, the detailed relationship between HMBOX1 and miR-885-5p is still unclear in cardiomyocytes.

Although many studies have been carried out, the function of miR-885-5p and HMBOX1 in cardiomyocytes and the role of sepsis in affecting myocardial function are still not fully understood. Therefore, in this study, using AC16 cells, we elaborated the roles of miR-885-5p/HMBOX1 in cardiomyocyte pyroptosis.

## Materials and Methods

### Blood Samples

Blood samples of sepsis patients and corresponding healthy individuals (*n* = 15) were recruited from the Zhongshan Hospital, Fudan University from June 2017 to October 2019, including 22 males and 8 females, age range 37–67 years old, mean age 52.1 ± 6.0 years old). Septic shock was defined as persisting hypotension requiring vasopressors to maintain mean arterial pressure (MAP) ≥ 65 mmHg and a serum lactate level > 2 mmol/L (18 mg/dl) despite adequate volume resuscitation (20). We excluded patients under 18 years old; those with pregnancy, severe anemia, active bleeding, platelet disorders, or chemotherapy; or those using full-dose heparin or any other medications that interfere with platelet function. The enrolled patients had 30 ml blood samples collected from a central venous catheter. Healthy volunteers provided blood samples that served as controls. All the patients were given informed consent. The study has the approval from the ethics committee of Zhongshan Hospital (B2021-390R), Fudan University.

### Cell Culture and Transfection

Human myocardial cells (AC16) were from ATCC. Cells were cultured using DMEM with 10% FBS under a 5% CO_2_ atmosphere at 37°C. Oligonucleotides including miR-885-5p mimic and the miR-885-5p inhibitor anti-miR-885-5p were used (Thermo Scientific, Lafayette, CO, USA) for overexpression or inhibition of miR-885-5p, respectively. For cell transfection assays, the synthetic oligonucleotides were transfected into cells using a Lipofectamine RNAiMAX Kit (Invitrogen, Carlsbad, CA, USA) at about 50% confluence. The media were changed 24 h post transfection and the indicated cells were used for further investigations. In some experiments, the Nuclear factor kappa B (NF-κB) inhibitor PDTC (10 μM; S1809, Beyotime Biotechnology, China) was dissolved in DMSO (Sigma-Aldrich, St. Louis, USA) and used to treat cells.

### Exosome Collection

Exosomes from sepsis patients (sepsis-exos) or controls (control-exos) were collected as described previously (21). In brief, blood serum was centrifuged at 2,000 g for 20 min to separate cellular debris, and the supernatant was further centrifuged twice at 1,000 g for 30 min twice using a 100-kDa MWCO hollow fiber membrane (Millipore, Bedford, MA, USA). Then, the supernatant was diluted, underlaid on 30% sucrose/D_2_O cushion (density 1.210 g/cm^3^), and ultracentrifugated at 100,000 g for 1.5 h at 4°C. Exosome-enriched fractions were centrifuged thrice at 1,000 g for 30 min, and filtered. In some experiments, AC16 cells were seeded (50,000 cells/well) and provided with PKH67-labeled exos for 4 h. Cells were observed using a fluorescence microscope.

### Transmission Electron Microscopic (TEM) Analysis

The exosome pellets were suspended in PBS and then fixed in paraformaldehyde (4%) and glutaraldehyde (4%) in PBS (0.1 M, pH 7.4) at 4°C for 5 min. After adding a drop of the exosomal sample, the carbon-coated copper grid was immersed in a phosphotungstic acid solution (2%, pH 7.0) for 30 s. The morphology and size of exos were observed using TEM analysis (JEM-1200EX; Jeol Ltd., Akishima, Japan).

### Exosome Endocytosis Assay

AC16 cells were seeded into 24-well plates. A total of 250 μg sepsis-exos or control-exos were labeled using PKH Lipophilic Membrane Dyes (cat. no. PKH67GL; Sigma-Aldrich, Merck KGaA) according to the manufacturer's instructions. PKH67-labeled exos were centrifuged (40,000 × g; 4°C; 70 min) and suspended in PBS (50 μl). Cells were incubated with normal medium (Thermo Fisher Scientific, Inc., Bremen, Germany) or medium containing PKH-67-labeled exos (20 μg/ml) at 37°C for 4 h. Subsequently, DAPI was used to stain the nucleus. Cells were observed under a fluorescent microscope (Olympus IX71; Olympus, Tokyo, Japan).

### Cecal Ligation and Puncture (CLP) Surgery Model

Cecal ligation and puncture-induced sepsis model was established as previously described. In brief, rats (200–250 g) were anesthetized with 1% pentobarbital, and a 2–3 cm incision was made to expose the caecum, which was punctured once with an 18-gauge needle. A small amount of feces was extruded from the hole to ensure patency. The abdominal incision was closed by applying sample running sutures. Rats underwent the same procedure except CLP was used as the sham group. All the rats were kept in warm water immediately after surgery. In some experiments, sepsis-exos (100 μg/kg) were i.v. injected into rats after surgery. Serum and heart tissues were harvested for study 72 h after surgery.

### Quantitative PCR (QPCR)

Total RNA was extracted from AC6 cells using TRIzol® reagent (Thermo Fisher Scientific, Bremen, Germany). Total RNA (0.5 μg) in 10 μl volume was reverse transcribed into cDNA using the RevertAid™ First Strand cDNA Synthesis kit (Thermo Fisher Scientific, Bremen, Germany) according to the manufacturer's protocol. RNAs were extracted, reverse transcribed into cDNA for qPCR with the following conditions: 95°C, 10 min followed by 40 × (95°C, 15 s; 60°C, 45 s). Fold changes were calculated by 2^−Δ*ΔCt*^. The primers were listed as follows: (5′ −3′): HMBOX1, GCA CAG GTA ACA GGT ATC AG, and CAT TCA GGG TCC TCT ATT GG; GAPDH, AAT CTC ATC AGC ATC TAC and AGT CTG TAG TCA TGC TCC; hsa-miR-885-5p, GCG CGT CCA TTA CAC TAC CCT and AGT GTA GCG TCG GAG GTG TT; U6, CTC GGT TAG GCC GCT CA and AAG GCT ACA CYA ATG TGG GT.

### Western Blot

Cells were lysed and total protein was quantified using a bicinchoninic acid assay (Beyotime Biotechnology, China). Proteins (20 μg) were separated *via* 12% SDS-PAGE and transferred onto polyvinylidene difluoride membranes at 300 mA for 120 min. The membranes were blocked with 5% BSA (Beyotime Biotechnology, China) for 1 h at room temperature. Subsequently, the membranes were incubated overnight at 4°C with the following primary antibodies diluted 1,000-fold that recognized HMBOX1 (16123-1-AP, Proteintech, USA), NLRP3 (Ab214185 and ab263899, Abcam, UK), pro-caspase-1 (ab179515, Abcam, USA), active caspase-1 (sc-398715, Santa), GSDMD-N (Ab215203 and ab219800, Abcam), NF-κB (#8242, CST, USA), H3 (#4499, CST). CD9 (1:1000, Ab92726, Abcam), CD63 (Ab271286, Abcam, UK), CD81 (1:700, Ab109201, Abcam), and GAPDH (1:5000, 60004-1-1G, Proteintech). Following primary incubation, the membranes were washed with TBS-Tween-20 (1%) and incubated with a horseradish peroxidase-conjugated goat antirabbit secondary antibody (1:10,000) at room temperature for 1 h. Protein bands were visualized using an enhanced chemiluminescence system (Thermo Fisher Scientific, Bremen, Germany).

### Enzyme-Linked Immunosorbent Assay (ELISA)

The levels of IL-1β and IL-18 in supernatants of AC16 cell and rat serum were assessed with ELISA kits (R&D Systems, Minneapolis, MN, USA) according to the manufacturer's instructions.

### Knockdown and Overexpression of HMBOX1

Short interfering RNAs targeting human HMBOX1 (siHMBOX1-1: 5′- GCA UUG GAA UGG UAG AUA ATT-3′, position: 337-355; siHMBOX1-2, 5′-GCA UCU UCC UCU ACA GCU ATT-3′, position: 641-659; and siHMBOX1-3 5′-CCU AGA UGU AGA UGA UAA ATT-3′, position: 865-883), along with a scrambled control siRNA (siNC, 5′-UUC UUC GCA CGU CUC ACG UAT-3′), were ligated into lentiviral vector pLKO.1. pLVX-puro having HMBOX1 or mock plasmids were transfected using Lipofectamine 2000 reagent (Thermo Fisher scientific).

### Flow Cytometry

To assess pyroptosis, active caspase-1 was labeled with caspase-1 antibody/FITC (active caspase-1, EL900443-100, Eterlife, USA) according to manufactures instructions and propidium iodide (PI) to mark cells with membrane pores (Life technology, Carlsbad, CA, USA). Cells that underwent pyroptosis were based on the detection of active caspase-1 and PI-positive (Caspase-1^+^PI^+^) populations.

### Luciferase Reporter Assay

The wild type (WT) human HMBOX1 3′UTR sequence was amplified *via* PCR and cloned into the Dual-Luciferase Reporter Assay System (psiCHECK-2 vector; Promega, Madison, WI, USA). To construct the mutant (mut) plasmid, the complementary sequences for miR-885-5p in the 3′UTR of HMBOX1 were mutated. AC16 cells were co-transfected with HMBOX1-WT or HMBOX1-mut and miR-885-5p mimic or miR-control. At 36 h after transfection, luciferase activities were detected using the dual-luciferase reporter assay system.

### Data Analysis

Data (mean ± SD) were analyzed by Prism 7.0 (GraphPad Software, San Diego, CA, USA). For comparative analyses of normally distributed variables, Student's *t*-tests and ANOVA were used. The nonparametric Mann-Whitney test was used when the distribution was not normal. *P* < 0.05 was defined as statistically significant.

## Results

### Sepsis-Exos Promoted the Pyroptosis of Human Myocardial Cells

**Figure 1:**
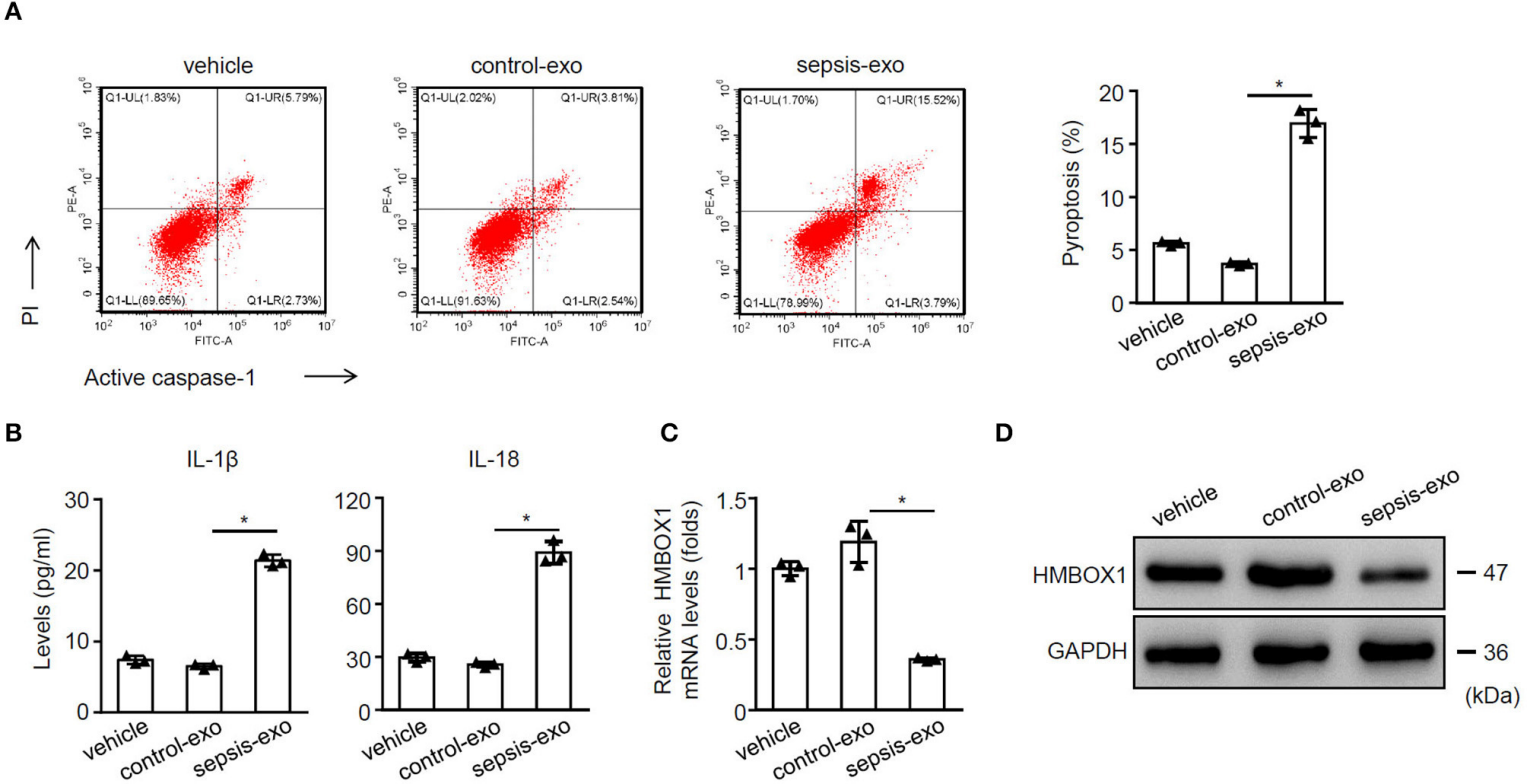
Sepsis-exos promoted the pyroptosis of human myocardial cells. Human myocardial cells AC16 were treated with 50 μg/ml exosomes isolated from sepsis patients (sepsis-exos) or corresponding healthy donors (control-exos) for 24 h. **(A)** PI^+^caspase-1^+^ pyroptosis was measured by flow cytometry. Representative images were shown on the left and summarized results are on the right. **(B)** Supernatant levels of IL-1β and IL-18 were measured by ELISA. **(C)** mRNA expression of HMBOX1 in cells was measured by qPCR. Data are normalized to expression levels of vehicle. **(D)** The protein level of HMBOX1 in cells were measured by western blot. **(A–C)** Data are mean ± SD from three independent experiments (*n* = 3 per group). *p < 0.05.

To study the effect of spesis-exos on pyroptosis, sepsis-exos, and control-exos were successfully isolated. The exos morphology was observed using TEM (Supplementary Figure 1A) and confirmed by exosomal markers CD9, CD63, and CD81 (Supplementary Figure 1B). Results also suggested that exos could be uptaken by AC16 cells (Supplementary Figure 1C). Sepsis-exos dramatically increased pyroptosis of AC16 cells compared with control-exos (Figure 1A). Sepsis-exos also enhanced the secretion of IL-1β and IL-18 (Figure 1B) and decreased HMBOX1 expression in mRNA (Figure 1C) and protein levels (Figure 1D). The data suggest that sepsis-exo treatment increased the pyroptosis, elevated inflammatory cytokines, but inhibited HMBOX1 in human myocardial cells.

### Sepsis-Exos Time-Dependently Promoted AC16 Pyroptosis

**Figure 2:**
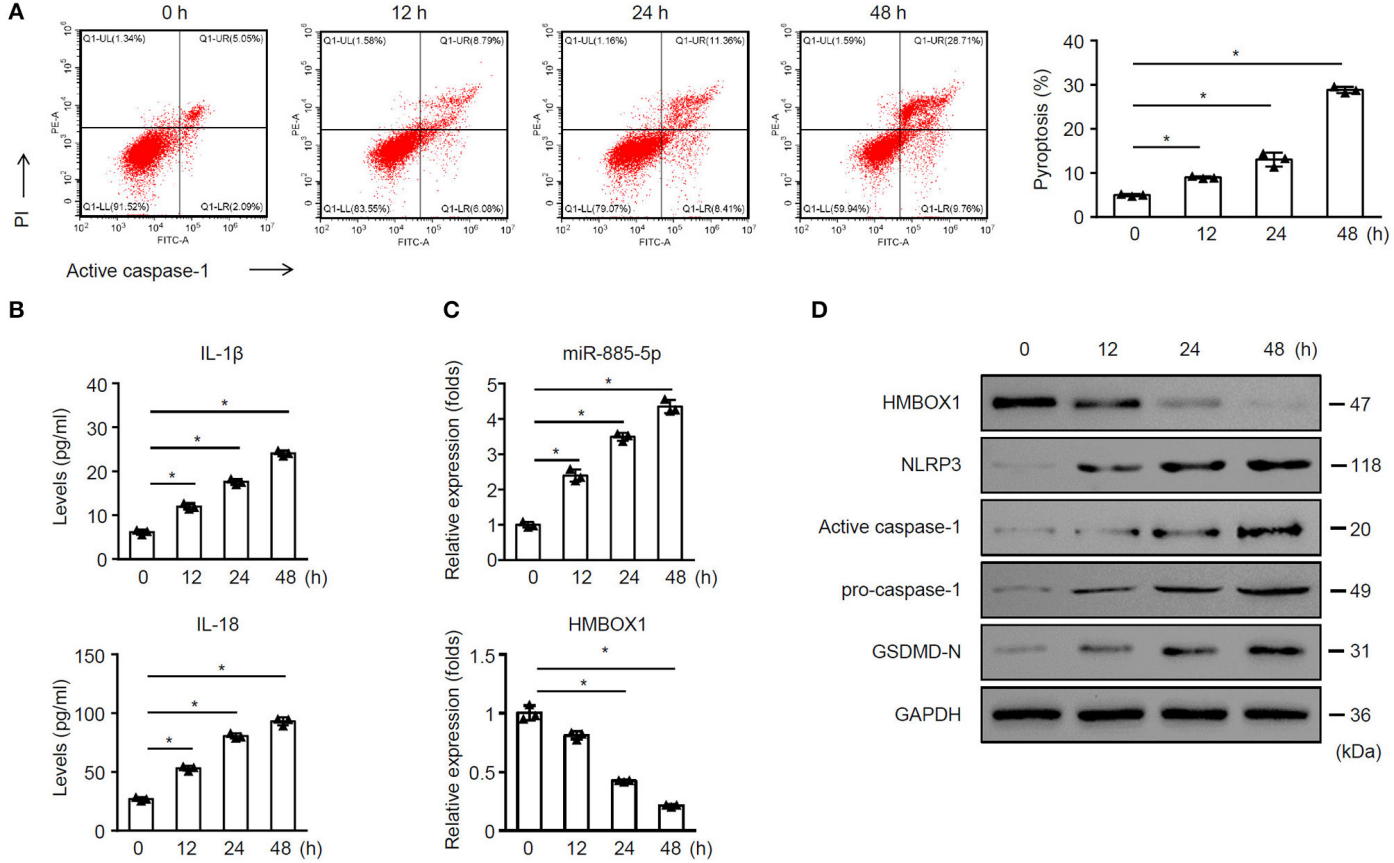
Sepsis-exos time-dependently promoted AC16 pyroptosis. AC16 cells were treated with sepsis-exos (50 μg/ml) for 0, 12, 24 and 48 h. **(A)** PI^+^caspase-1^+^ pyroptosis was measured by flow cytometry. Representative images were shown on the left and summarized results are on the right. **(B)** Supernatant levels of IL-1β and IL-18 were measured by ELISA. **(C)** Levels of miR-885-5p and HMBOX1 in cells was measured by qPCR. Data are normalized to expression levels of 0 h data. **(D)** Protein levels of HMBOX1, NLRP3, caspase-1, and GSDMD-N were measured by western blot. **(A–C)** Data are mean ± SD from three independent experiments (*n* = 3 per group). * *p* < 0.05.

The further time-course study indicated that sepsis-exos time-dependently promoted AC16 pytoptosis (Figure 2A). Sepsis-exos time-dependently elevated IL-1β and IL-18 (Figure 2B), increased miR-885-5p but decreased HMBOX1 expression (Figure 2C). Sepsis-exos also decreased the expression of HMBOX1 but increased the levels of NLRP3, active caspase-1, pro-caspase-1, and GSDMD-N (Figure 2D). The results indicated sepsis-exos time-dependently enhanced AC16 pyroptosis.

### Sepsis-Exos Promote the Severity of Sepsis in Rats

**Figure 3:**
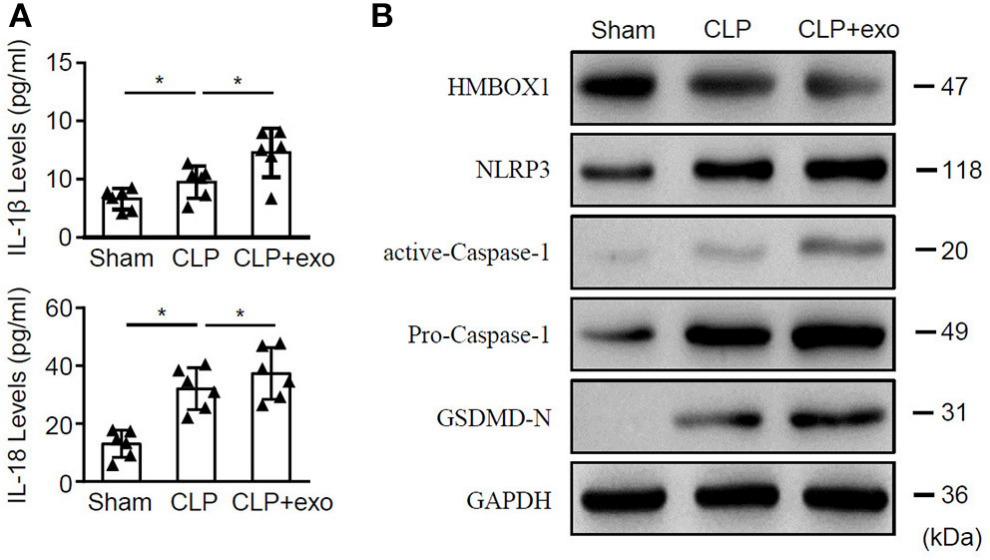
Sepsis-exos promote sepsis *in vivo*. Septic rats underwent CLP surgery were i.v. injected with sepsis-exos (100 μg/kg). Serum and heart tissues from sham, CLP, or sepsis-exo-treated mice were sampled 72 h after surgery. **(A)** Serum levels of IL-1β and IL-18 were measured by ELISA. Data are mean ± SD from three independent experiments (*n* = 6 mice per group). **(B)** Protein levels of HMBOX1, NLRP3, caspase-1, and GSDMD-N in hearts were measured by western blot. * *p* < 0.05.

To further elucidate the impact of sepsis-exos *in vivo*, septic rats underwent CLP surgery were treated with sepsis-exos. Sham rats served as control. Treatment of sepsis-exos significantly increased the serum levels of IL-1β and IL-18 72 h after surgery (Figure 3A). Moreover, levels of HMBOX1 in heart tissues were dramatically decreased, while expression of pyroptosis-related proteins NLRP3, caspase-1, and GSDMD-N were all upregulated after treatment of sepsis-exos (Figure 3B). These data demonstrated that sepsis-exos increased the inflammatory cytokines is associated with increased pyroptosis-related proteins of hearts in septic rats.

### miR-885-5p Modulated Pyroptosis of AC16 Cells

**Figure 4:**
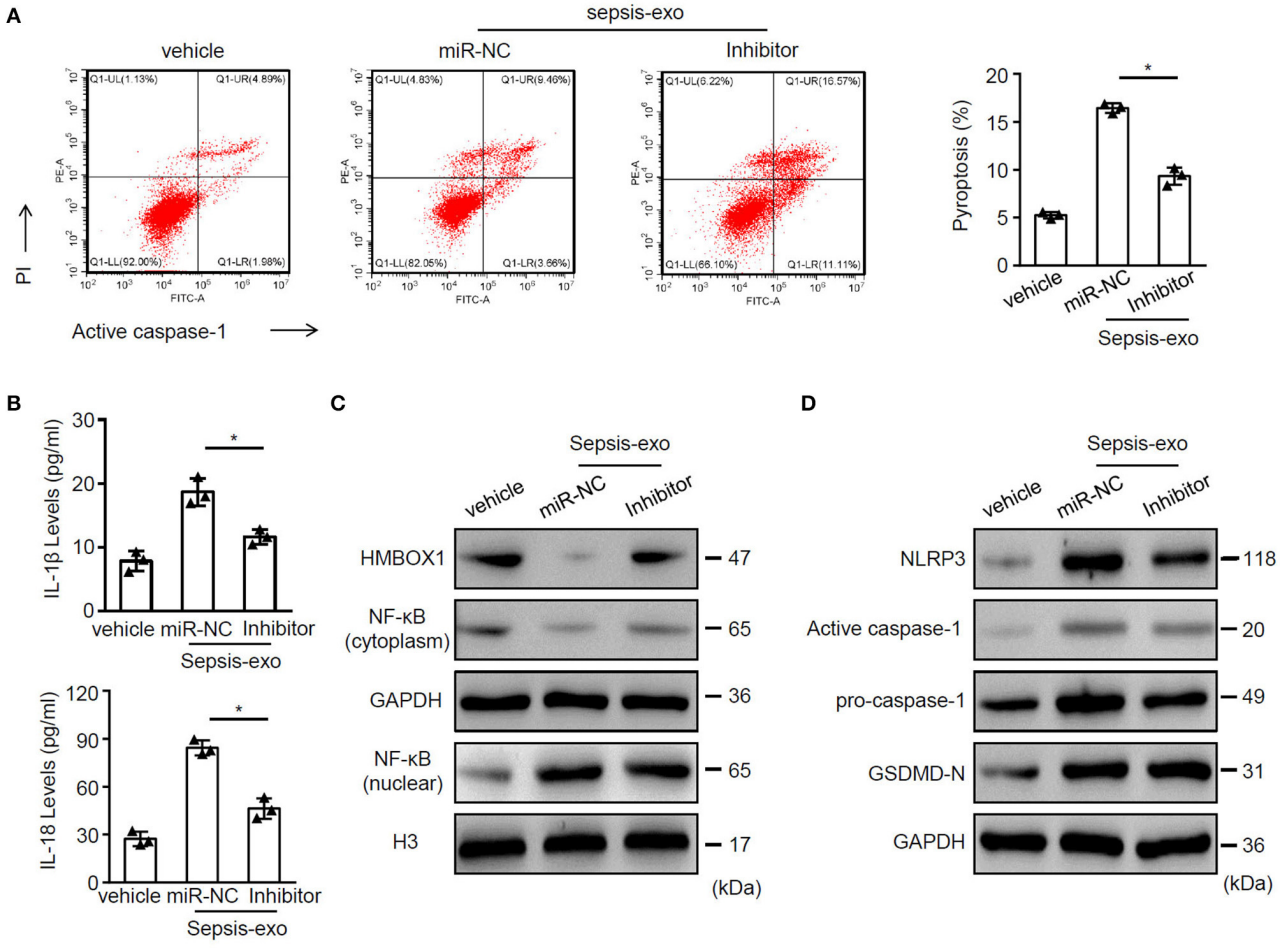
Inhibition of miR-885-5p suppressed sepsis-exo-induced cell pyroptosis. AC16 cells transfected with anti-miR-885-5p (Inhibitor) or anti-miR-control (miR-NC) were treated with sepsis-exos (50 μg/ml). **(A)** PI^+^caspase-1^+^ pyroptosis was measured by flow cytometry. Representative images were shown on the left and summarized results are on the right. **(B)** Supernatant levels of IL-1β and IL-18 were measured by ELISA. The protein levels of HMBOX1, NF-κB (cytoplasm), NF-κB (nuclear) **(C)** and NLRP3, caspase-1, GSDMD-N **(D)** were measured by western blot. **(A,B)** Data are mean ± SD from three independent experiments (*n* = 3 per group). * *p* < 0.05.

Given that miR-885-5p was aberrantly expressed in sepsis-exos, we hypothesized that miR-885-5p may regulate AC16 cell pyroptosis. To test our hypothesis, sepsis-exos were used to treat AC16 cells, with or without miR-885-5p inhibitor. We found that inhibition of miR-885-5p significantly suppressed AC16 pyroptosis (Figure 4A) and the secretion of IL-1β and IL-18 (Figure 4B). Besides, treatment of miR-885-5p inhibitor upregulated the expression of HMBOX1 and NF-κB (cytoplasm), but decreased nuclear NF-κB, NLRP3, caspase-1, and GSDMD-N (Figures 4C,D).

**Figure 5:**
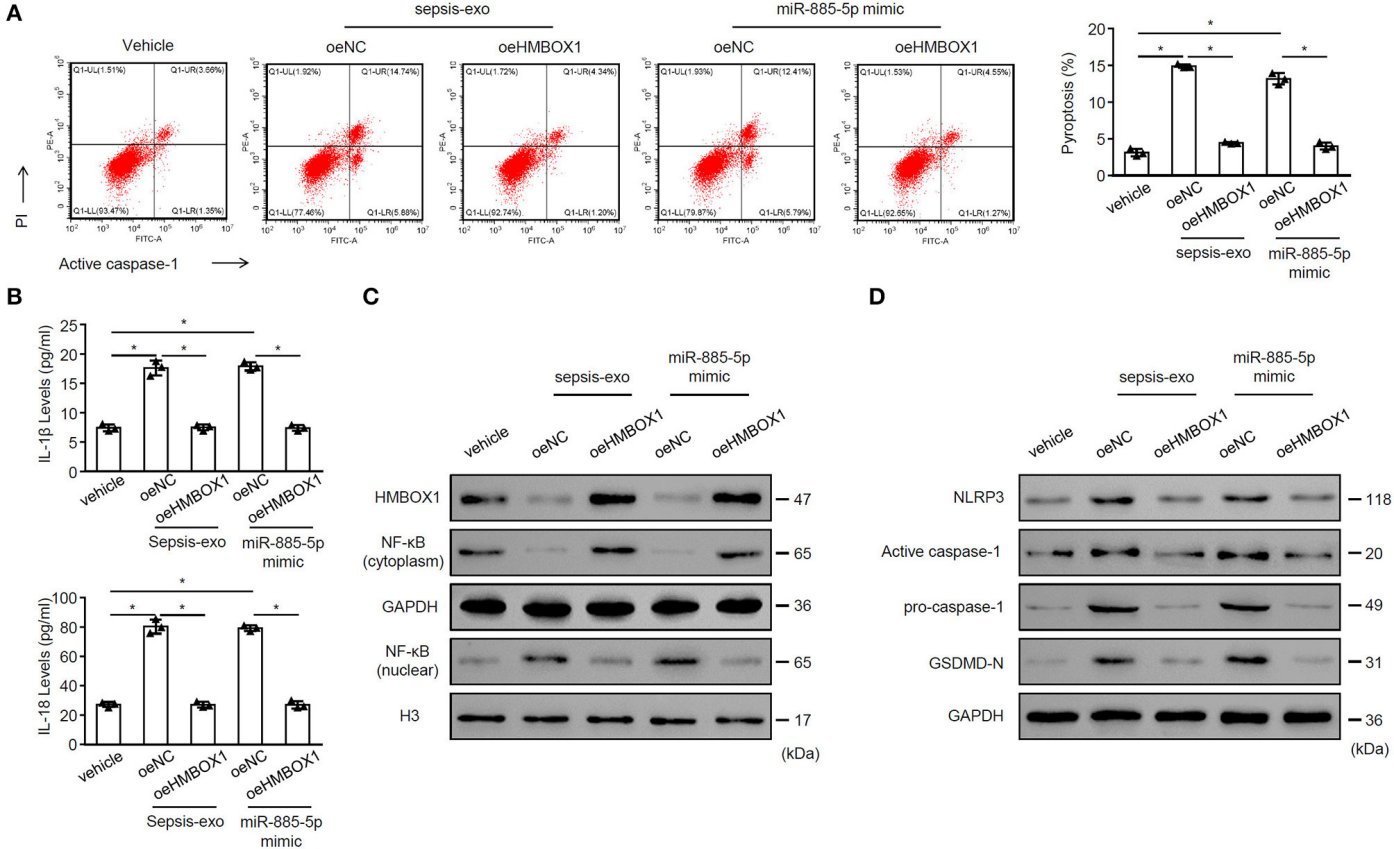
MiR-885-5p mimics and sepsis-exos showed similar effects. AC16 cells with HMBOX1 overexpression (oeHMBOX1) or not (oeNC) were treated with sepsis-exos (50 μg/mL) or miR-885-5p mimic for 24 h. **(A)** PI^+^caspase-1^+^ pyroptosis was measured by flow cytometry. Representative images were shown on the left and summarized results are on the right. **(B)** Supernatant levels of IL-1β and IL-18 were measured by ELISA. The protein levels of HMBOX1, NF-κB (cytoplasm), NF-κB (nuclear) **(C)** and NLRP3, caspase-1, GSDMD-N **(D)** were measured by western blot. **(A,B)** Data are mean ± SD from three independent experiments (*n* = 3 per group). *p < 0.05.

To evaluate whether overexpressing HMBOX1 might reverse the effect of miR-885-5p, sepsis-exos or miR-885-5p mimic was used to treat AC16 cells, with or without HMBOX1 overexpression. We found that both sepsis-exos and miR-885-5p mimic promoted AC16 pyroptosis, which was abolished by overexpressing HMBOX1 (Figure 5A). The elevated secretion of IL-1β and IL-18, and expression pattern of HMBOX1, NF-κB (cytoplasm), nuclear NF-κB, NLRP3, caspase-1, and GSDMD-N were all reversed by overexpression of HMBOX1 (Figures 5B–D), showing that sepsis-exos and miR-885-5p mimic had similar effects on AC16 cells. These data demonstrated that miR-885-5p modulated AC16 pyroptosis is associated with HMBOX1.

### miR-885-5p Suppressed HMBOX1 *via* Binding to 3′UTR

**Figure 6:**
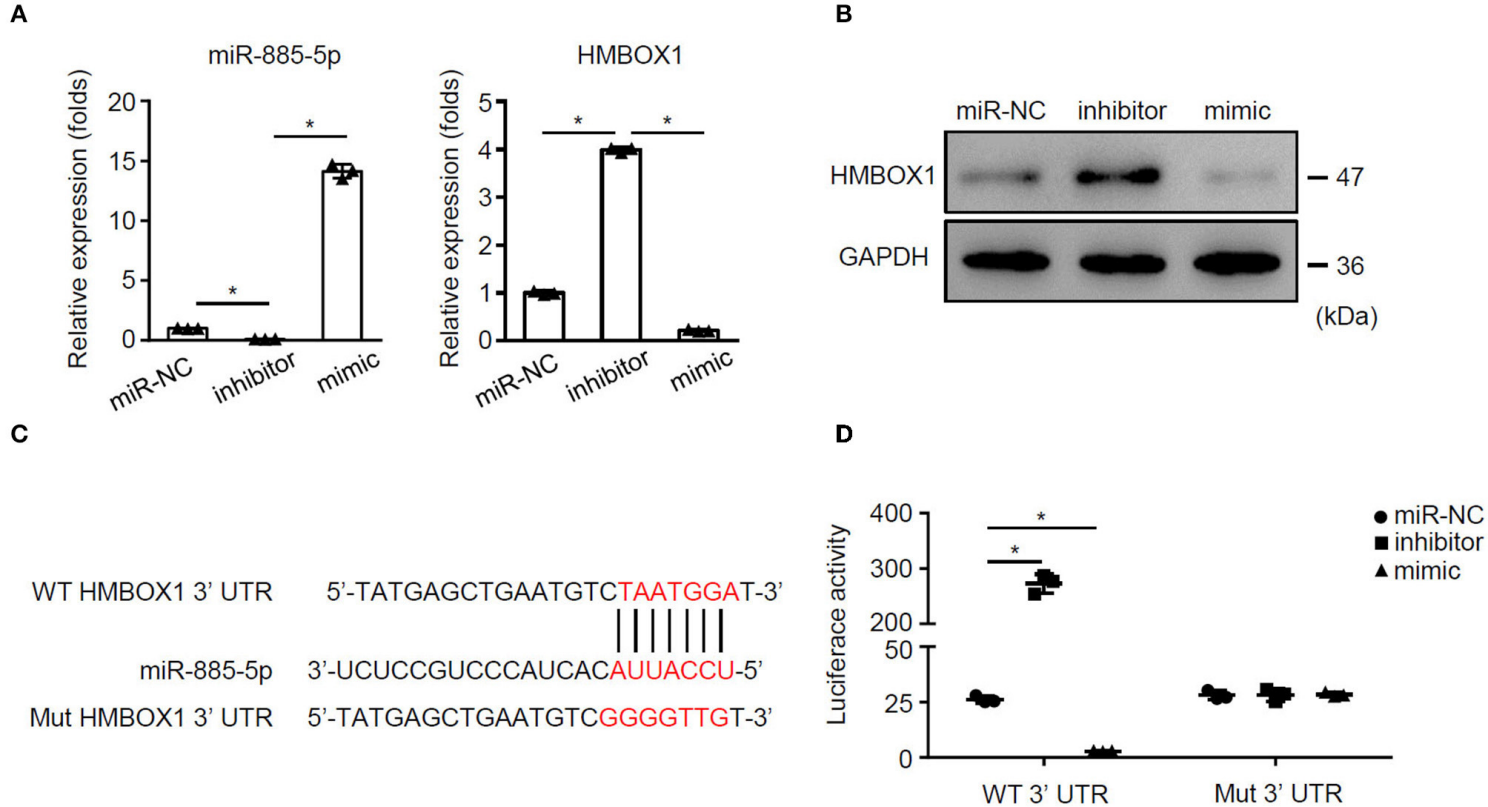
miR-885-5p inhibited HMBOX1 through binding on its 3′UTR. AC16 cells were treated with anti-miR-control (miR-NC), inhibitor or mimic of miR-885-5p. **(A)** Levels of miR-885-5p and HMBOX1 in cells was measured by qPCR. Data are normalized to expression levels of miR-control. **(B)** Protein levels of HMBOX1 were measured by western blot. **(C)** Wild type (WT) or mutant (mut) miR-885-5p binding sites. **(D)** Luciferase activity was determined 36 h after transfection. (A,D) Data are mean ± SD from three independent experiments (*n* = 3 per group). * *p* < 0.05.

To investigate the mechanism of miR-885-5p's regulation on HMBOX1 expression, miR-885-5p was silenced/overexpressed. Silencing miR-885-5p increased HMBOX1 while overexpressing miR-885-5p decreased HMBOX1 (Figures 6A,B). Bioinformatic analysis showed a binding site of miR-885-5p in 3′UTR of HMBOX1 (Figure 6C). Next, HMBOX1 HMBOX1 3′UTR-WT/3′UTR-Mut and miR-885-5p inhibitor/mimics were transfected. Inhibiting enhanced HMBOX1 promoter activity, which was suppressed by miR-885-5p overexpression. Mutating HMBOX1's miR-885-5p binding site abolished miR-885-5p's effect (Figure 6D). The data suggested that miR-885-5p is bound to 3′UTR to inhibit HMBOX1.

### Nuclear Factor Kappa B Inhibitor PDTC Rescued the Function of HMBOX1 in AC16 Cells

**Figure 7:**
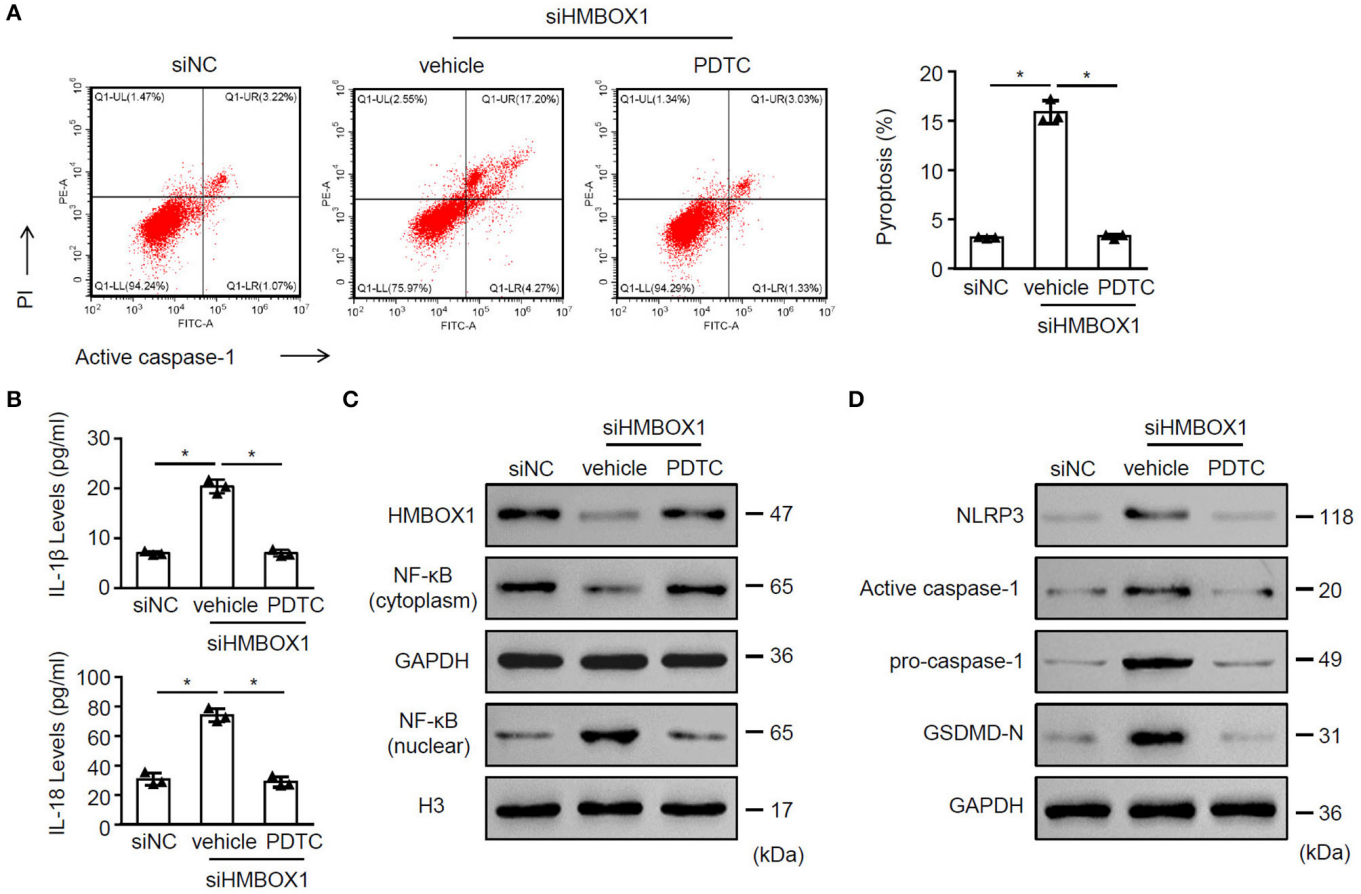
The NF-κB inhibitor PDTC rescued the function of HMBOX1 in AC16 cells. AC16 cells with HMBOX1 silencing (siHMBOX1) or not (siNC) were treated with NF-κB inhibitor PDTC (10 μM) for 24 h. **(A)** PI^+^caspase-1^+^ pyroptosis was measured by flow cytometry. Representative images were shown on the left and summarized results are on the right. **(B)** Supernatant levels of IL-1β and IL-18 were measured by ELISA. The protein levels of HMBOX1, NF-κB (cytoplasm), NF-κB (nuclear) **(C)** and NLRP3, caspase-1, GSDMD-N **(D)** were measured by western blot. **(A,B)** Data are mean ± SD from three independent experiments (*n* = 3 per group). * *p* < 0.05.

To understand the role of NF-κB in pyroptosis, NF-κB inhibitor PDTC was introduced in this study. Silencing of HMBOX1 caused significant increase of pyroptosis in AC16 cells, which was blocked by the administration of NF-κB inhibitor PDTC (Figure 7A). Silencing of HMBOX1 also resulted in the elevated secretion of IL-1β and IL-18, which was diminished by PDTC treatment (Figure 7B). Administration of NF-κB inhibitor PDTC also blocked HMBOX1 silencing-caused downregulation of HMBOX1 and NF-κB (cytoplasm), and upregulation of NF-κB (nuclear), NLRP3, caspase-1, and GSDMD-N in AC16 cells (Figures 7C,D). These findings demonstrated that the administration of NF-κB inhibitor PDTC rescued the effect of HMBOX1 silencing on AC16 cells.

## Discussion

We showed that sepsis-exo treatment elevated IL-1β and IL-18, decreased HMBOX1, and promoted pyroptosis in AC16 cells. The mechanistic study indicated that miR-885-5p negatively regulates HMBOX1. Overexpression of HMBOX1 significantly reduced the cardiomyocyte pyroptosis caused by sepsis-exos or miR-885-5p mimic.

MicroRNAs (miRNAs) function by negatively regulating their target genes. Different miR-885-5p targets have been shown over the years. Afanasyeva et al. demonstrated that miR-885-5p suppresses neuroblastoma *via* inhibiting cyclin-dependent kinase (9). Yu et al. indicated that overexpressing miR-885-5p enhanced proliferation of MG-63 cells by targeting cell division cycle protein 73 homolog (22). Xu et al. have demonstrated that miR-885-5p overexpression suppressed hepatocellular carcinoma cell proliferation through regulating its target hexokinase 2 (23). HMBOX1 is characterized by containing homeobox domain (24). Studies revealed that HMBOX1 promotes the proliferation of gastric cancer cells (25). Loss of HMBOX1 has been demonstrated to promote LPS-induced apoptosis of vascular endothelial cells (26). Our data indicated that miR-885-5p negatively regulated HMBOX1. Overexpressing HMBOX1 reversed miR-885-5p-induced elevation of inflammatory factors, and upregulation of nuclear NF-κB, NLRP3, caspase-1, and GSDMD-N, and promotion of AC16 pyroptosis. The results increase our knowledge of miR-885-5p/HMBOX1 in the injury of cardiomyocytes and broaden our understanding of septic myocardial depression.

Pyroptosis involves different diseases. For example, dysregulation of pyroptosis led to tissues damage (27). Pyroptosis has also been related to type II diabetic mellitus through activated caspase-1 and elevated IL-1β/IL-18 (28). In gastric cancer cells, increased GSDMD inhibits tumor development through cell cycle arrest (29). An *in vivo* study by Lei et al. demonstrated that pyroptosis contributes to cardiomyocyte injury in myocardial infarction (30). We showed that sepsis-exo or miR-885-5p mimic not only elevated IL-1β and IL-18, but also increased NLRP3, caspase-1, and GSDMD-N, leading to promotion of pyroptosis. These results demonstrated the significance of pyroptosis in regulating miR-885-5p/HMBOX1-mediated cardiomyocyte injury. We also further showed that miR-885-5p/HMBOX1-mediated cardiomyocyte injury is NF-κB-dependent. It has been confirmed that activation of the NF-κB-dependent pathway induces NLRP3-mediated pyroptosis (31). In this study, our results indicated that overexpression of HMBOX1 inhibited the translocation of NF-κB from the cytoplasm to nuclear in sepsis-exo-treated AC16 cells. Importantly, the NF-κB inhibitor PDTC rescued the function of HMBOX1 in siHMBOX1 transfecting cells. Hence, these findings demonstrated that HMBOX1 is involved in the network of NF-κB-dependent pathways and suppressed the translocation of NF-κB from the cytoplasm to nuclear. Moreover, these findings indicate the functional role of miR-885-5p/HMBOX1 in cardiomyocyte injury, and therefore, improve our knowledge of septic myocardial depression. Although future studies based on animals or clinical specimens can provide more relevant and pervasive data, we show a novel mechanism underlying septic myocardial depression. However, although our present data showed that exsomal miR-885-5p plays a critical role in human myocardial cell pyroptosis, we still cannot exclude that miR-885-5p might work in cell apoptosis. The cellular and molecular mechanisms of exsomal miR-885-5p in other forms of cell death need further investigation.

**Figure 8:**
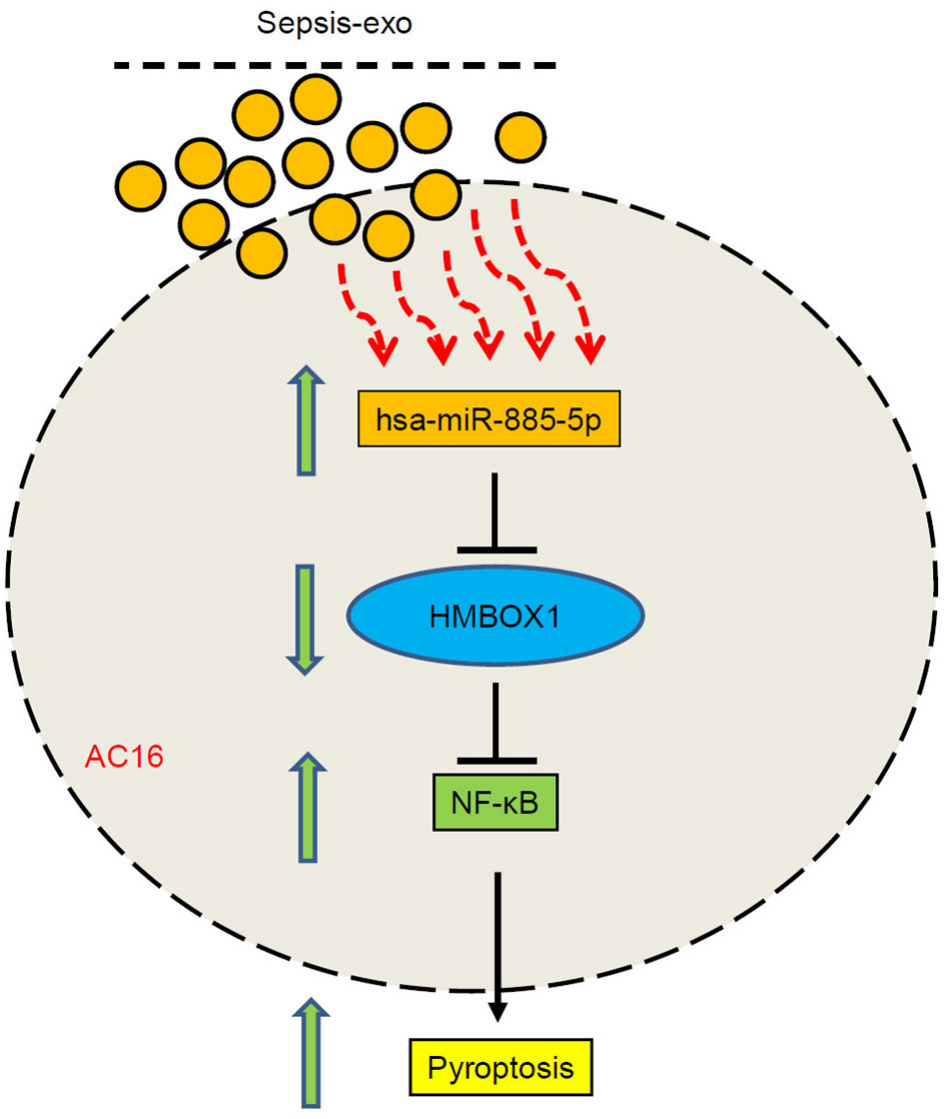
A schematic diagram showing that sepsis-exos induced cardiomyocytes pyroptosis by regulating miR-885-5p/HMBOX1 *via* NF-κB signaling.

In short, this study revealed a new function of miR-885-5p/HMBOX1 signaling, showing that sepsis-exos-induced cardiomyocytes damage *via* miR-885-5p and HMBOX1 (Figure 8). These findings identified an important role of the miR-885-5p/HMBOX1 axis which is relevant to cardiomyocytes pyroptosis, and might contribute to septic myocardial depression treatment.

## Data Availability Statement

The raw data supporting the conclusions of this article will be made available by the authors, without undue reservation.

## Ethics Statement

The studies involving human participants were reviewed and approved by Ethics Committee of Zhongshan Hospital, Fudan University. The patients/participants provided their written informed consent to participate in this study.

## Author Contributions

YZ and ZL conceived the project, designed the project, and approved the final manuscript. G-wT drafted the manuscript. G-wT, J-fM, and J-kL conducted the experiments. YS, J-cL, G-wH, and Y-rC analyzed data and contributed to experiments. All authors contributed to the article and approved the submitted version.

## Funding

This article was supported by grants from the Natural Science Foundation of Shanghai (20ZR1411100 to ZL, 21ZR1412900 to YS), Science and Technology Commission of Shanghai Municipality (20DZ2261200 to ZL), Program of Shanghai Academic/Technology Research Leader (20XD1421000 to G-wT), National Natural Science Foundation of China (82070085 to G-wT), Construction program of key but weak disciplines of shanghai health commission (2019ZB0105 to ZL), Clinical Research Project of Zhongshan Hospital (2020ZSLC38 to G-wT, 2020ZSLC27 to ZL), Smart Medical Care of Zhongshan Hospital (2020ZHZS01 to ZL), and Project for elite backbone of Zhongshan Hospital (2021ZSGG06 to G-wT).

## Conflict of Interest

The authors declare that the research was conducted in the absence of any commercial or financial relationships that could be construed as a potential conflict of interest.

## Publisher's Note

All claims expressed in this article are solely those of the authors and do not necessarily represent those of their affiliated organizations, or those of the publisher, the editors and the reviewers. Any product that may be evaluated in this article, or claim that may be made by its manufacturer, is not guaranteed or endorsed by the publisher.

## Supplementary Material

Supplementary Figure 1 Characterization of exosomes. **(A)** Sepsis-exos and control-exos were subjected to TEM observation. Scale bar, 1 μm. **(B)** The protein levels exosome markers CD9, CD63, and CD81 were measured by western blot. **(C)** The exosome endocytosis assay was observed by a fluorescent microscope. Scale bar, 25 μm. **(A–C)** Data are from three independent experiments. ^*^ *p* < 0.05.

Supplementary Figure 2 Overexpression and knockdown of HMBOX1 in AC cells. **(A,B)** HMBOX1 was overexpressed in AC16 cells. **(A)** mRNA expression of HMBOX1 in cells was measured by qPCR. Data are normalized to expression levels of control. **(B)** The protein level of HMBOX1 in cells were measured by western blot. **(C,D)** HMBOX1 was silenced in AC cells with 3 siRNAs, respectively. **(C)** mRNA expression of HMBOX1 in cells was measured by qPCR. Data are normalized to expression levels of control. **(D)** The protein level of HMBOX1 in cells were measured by western blot. **(A–C)** Data are mean ± SD from three independent experiments (*n* = 3 per group). ^*^ *p* < 0.05.
