# Supplementary material for: Exosome-Derived From Sepsis Patients' Blood Promoted Pyroptosis of Cardiomyocytes by Regulating miR-885-5p/HMBOX1
Source: Front Cardiovasc Med. 2022 Mar 8;9:774193. doi: 10.3389/fcvm.2022.774193 (PMC8957255; doi:10.3389/fcvm.2022.774193)
Supplement: Supplementary file 1 [file Data_Sheet_1.ZIP › Source data-Fig.1/Fig.1A/Gating strategies.docx]

AC16 cells with different treatment were loaded and analyzed by flow cytometry. After gating live cells according to FSC and SSC, cells were further analyzed by dot plots labeled with FITC-active caspase-1 and PE-PI.
