# Supplementary figures and images for: Exosome-Derived From Sepsis Patients' Blood Promoted Pyroptosis of Cardiomyocytes by Regulating miR-885-5p/HMBOX1
Source: Front Cardiovasc Med. 2022 Mar 8;9:774193. doi: 10.3389/fcvm.2022.774193 (PMC8957255; doi:10.3389/fcvm.2022.774193)

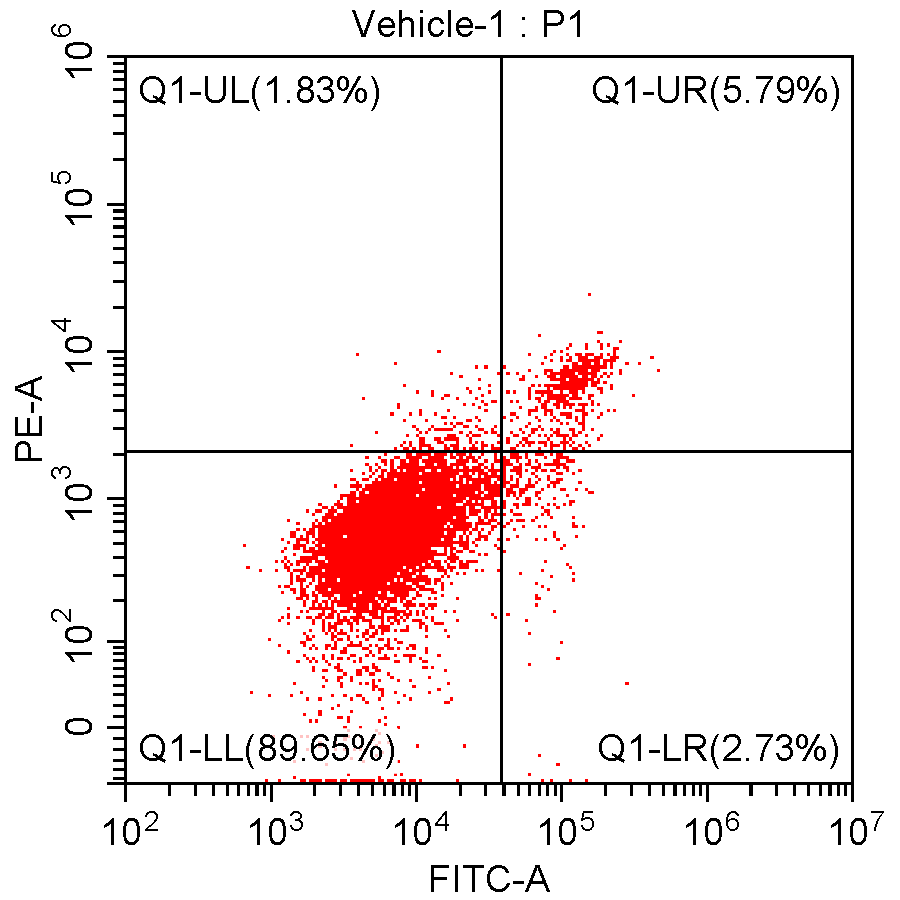

Supplement: Supplementary file 1 [file Data_Sheet_1.ZIP › Source data-Fig.1/Fig.1A/Vehicle-1.bmp]

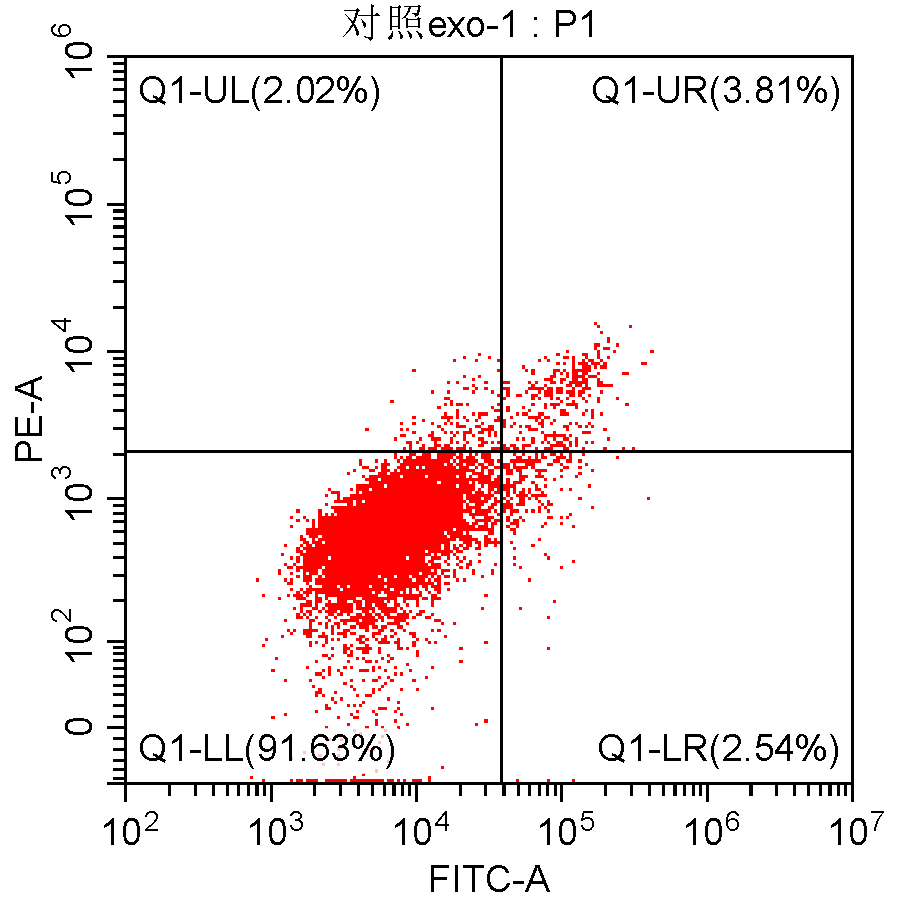

Supplement: Supplementary file 1 [file Data_Sheet_1.ZIP › Source data-Fig.1/Fig.1A/control-exo.bmp]

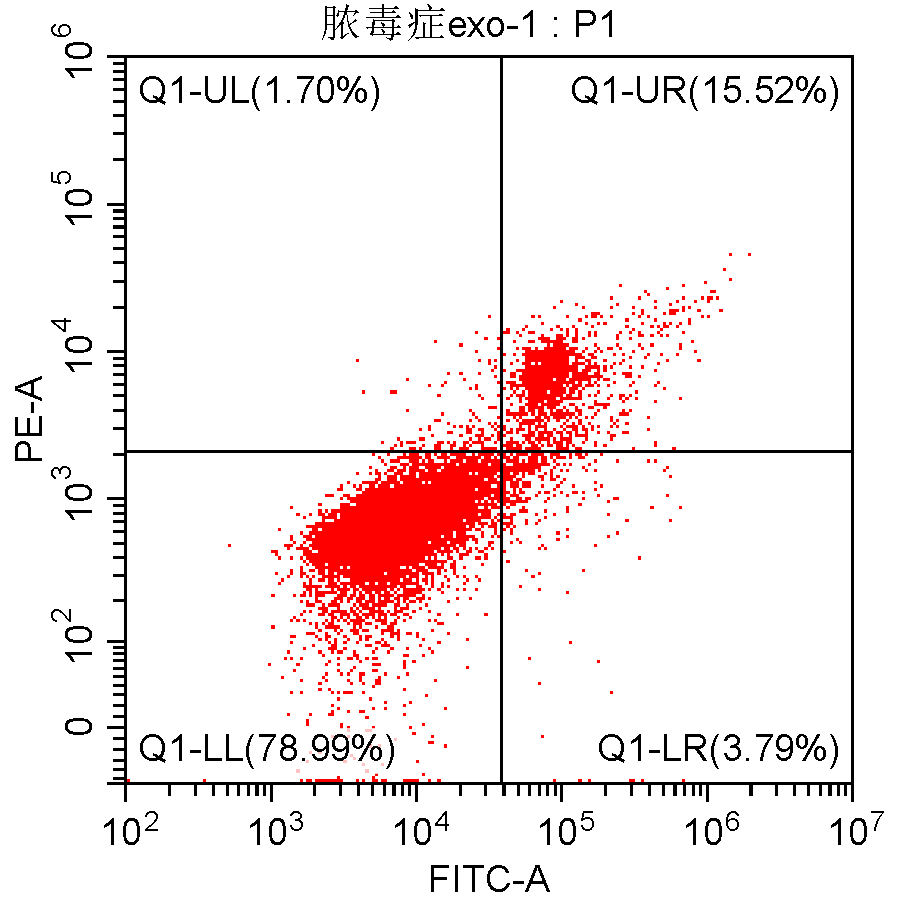

Supplement: Supplementary file 1 [file Data_Sheet_1.ZIP › Source data-Fig.1/Fig.1A/spssis-exo.bmp]

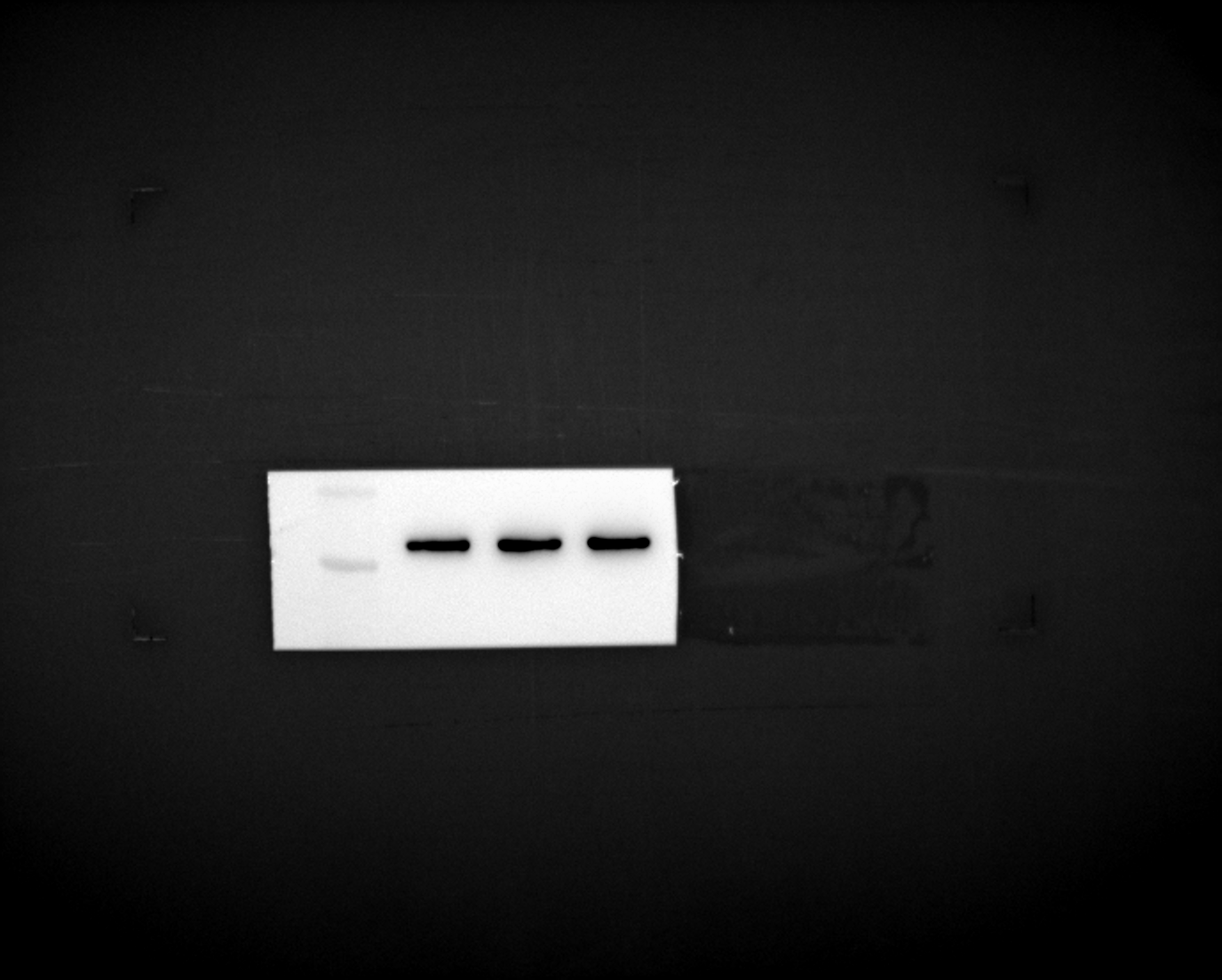

Supplement: Supplementary file 1 [file Data_Sheet_1.ZIP › Source data-Fig.1/Fig.1D/GAPDH.tif]

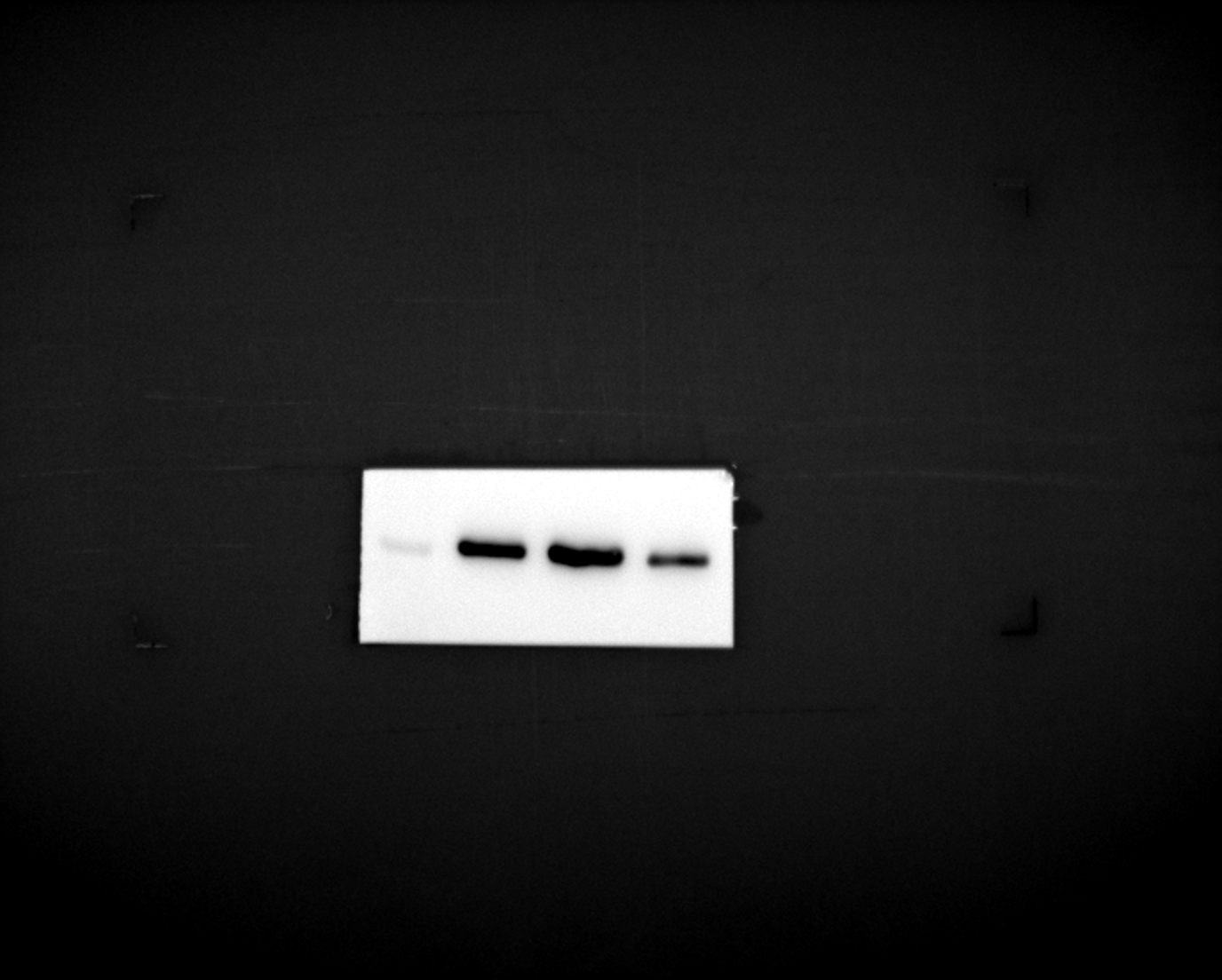

Supplement: Supplementary file 1 [file Data_Sheet_1.ZIP › Source data-Fig.1/Fig.1D/HMBOX1.tif]

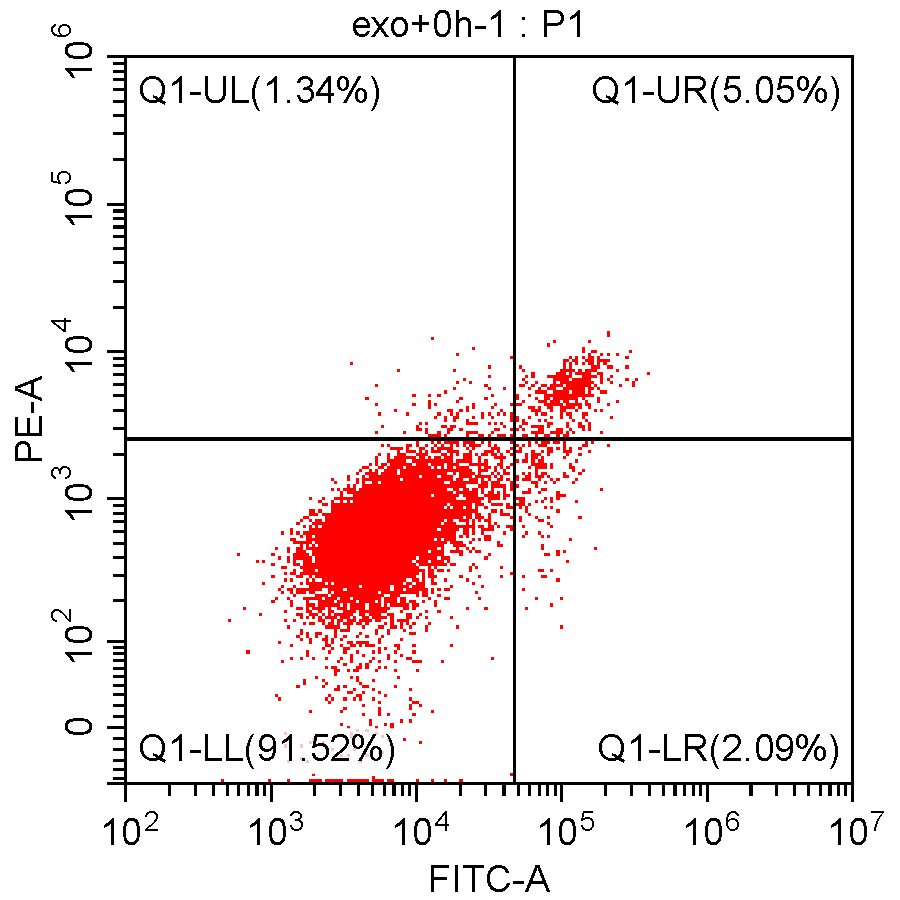

Supplement: Supplementary file 2 [file Data_Sheet_2.ZIP › Source data-Fig.2/Fig.2A/0h.bmp]

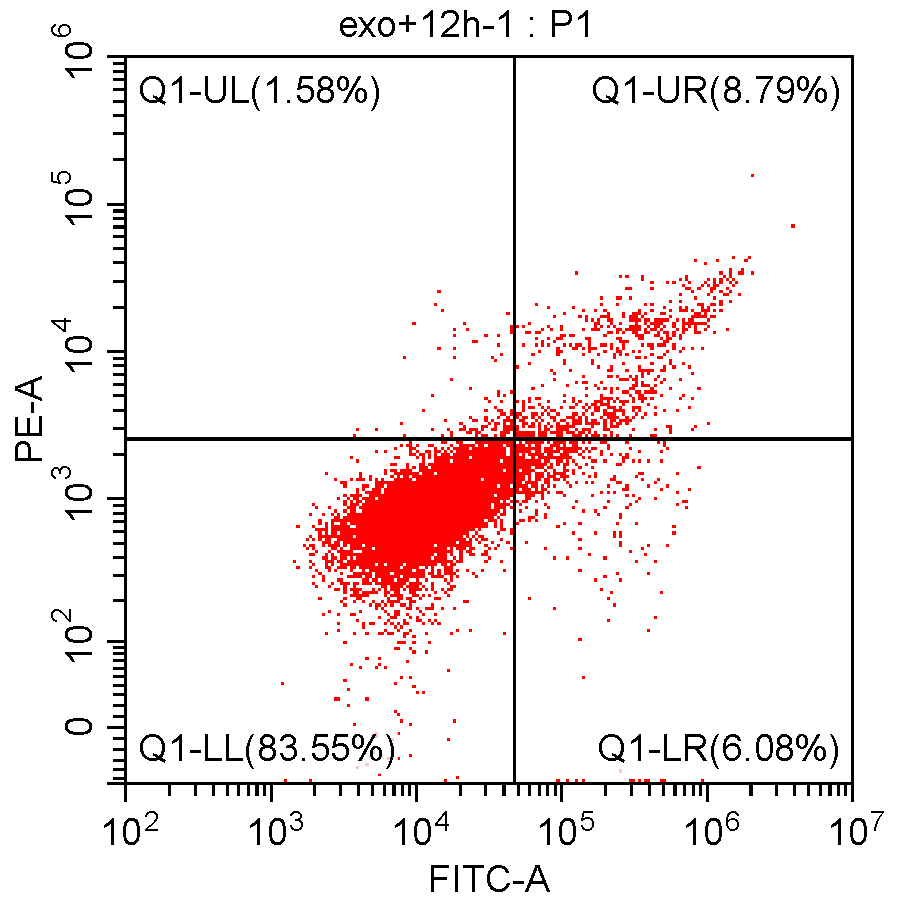

Supplement: Supplementary file 2 [file Data_Sheet_2.ZIP › Source data-Fig.2/Fig.2A/12h.bmp]

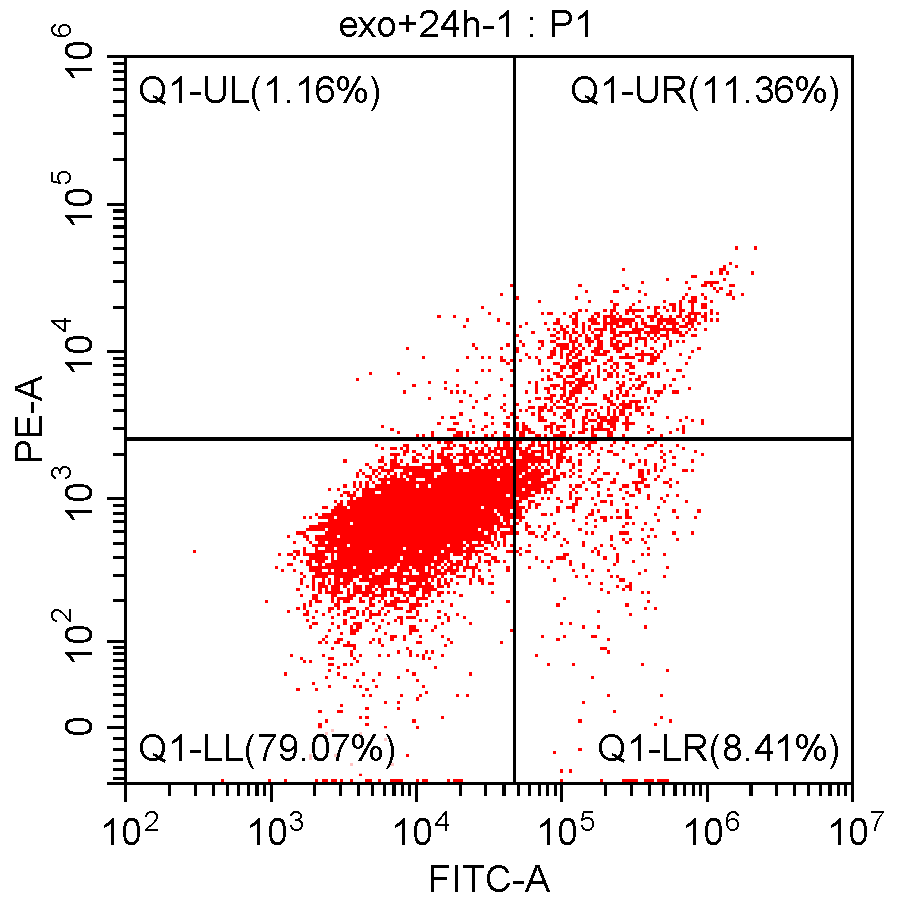

Supplement: Supplementary file 2 [file Data_Sheet_2.ZIP › Source data-Fig.2/Fig.2A/24h.bmp]

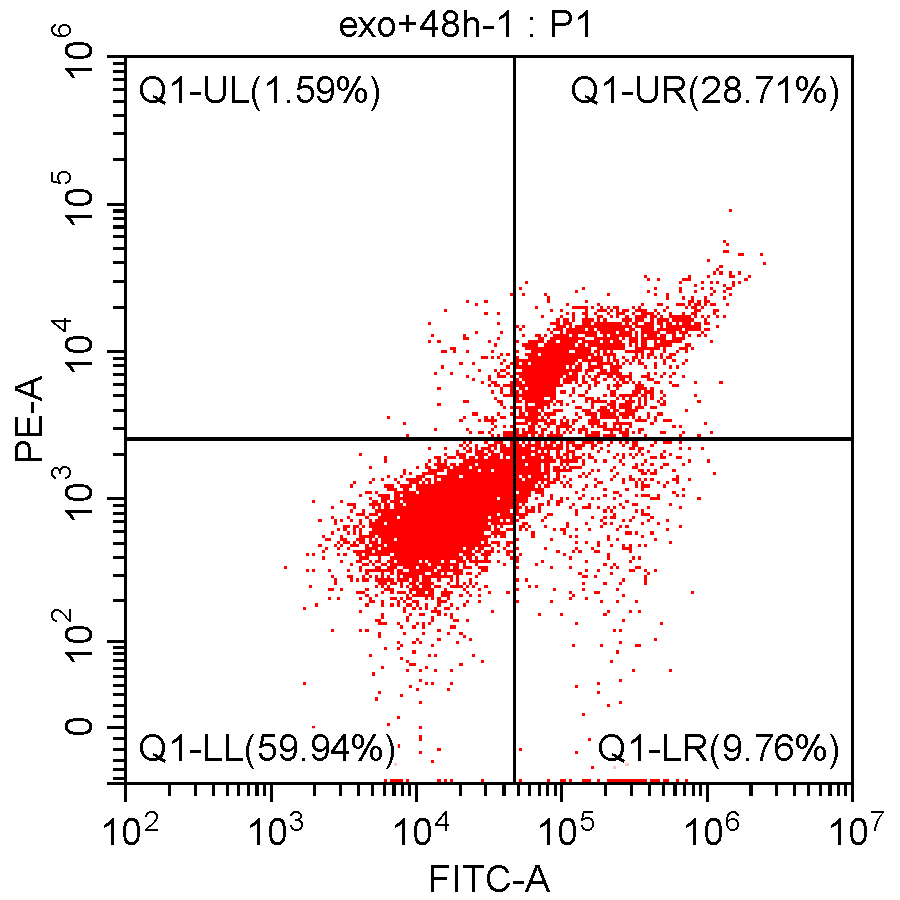

Supplement: Supplementary file 2 [file Data_Sheet_2.ZIP › Source data-Fig.2/Fig.2A/48h.bmp]

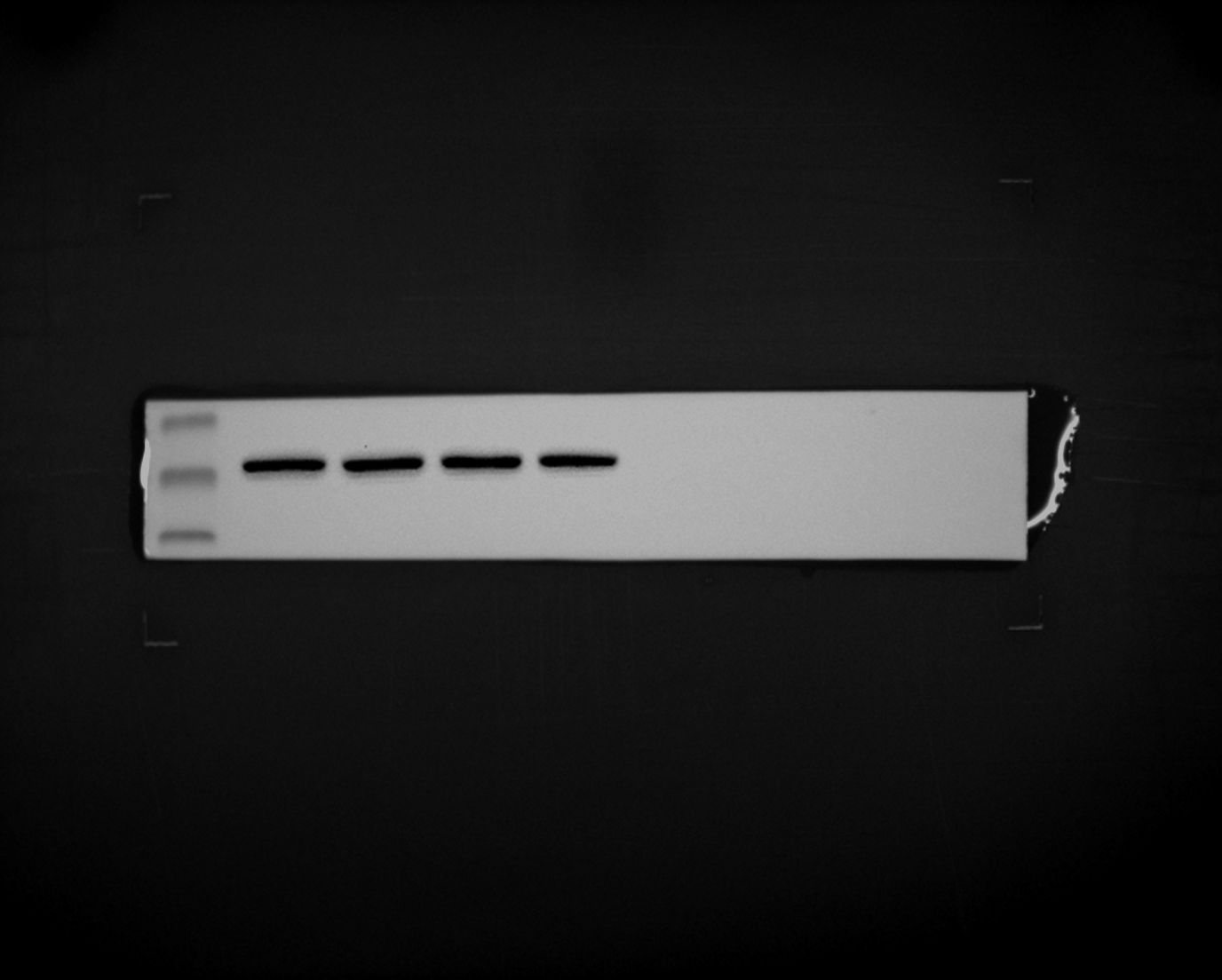

Supplement: Supplementary file 2 [file Data_Sheet_2.ZIP › Source data-Fig.2/Fig.2D/GAPDH.tif]

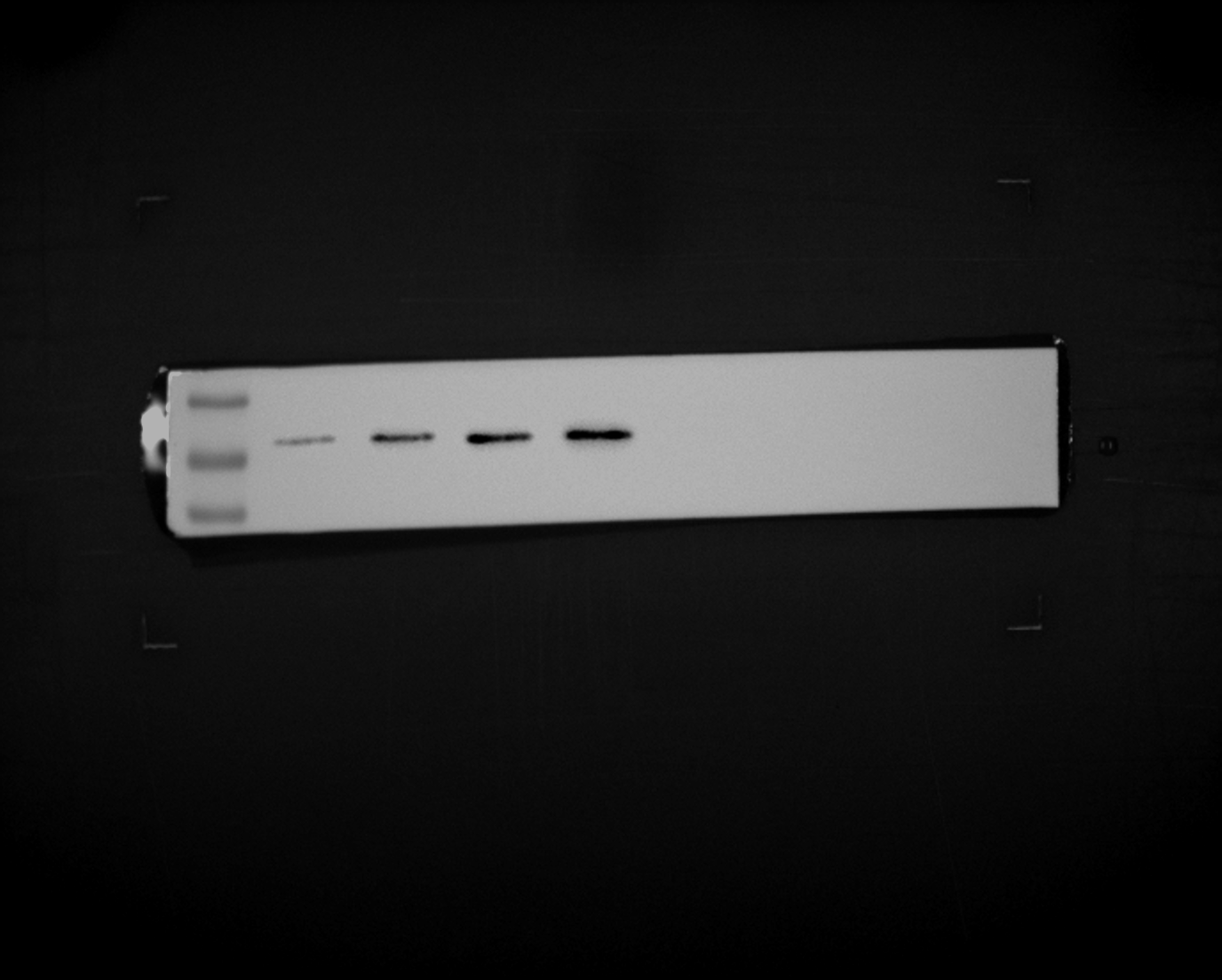

Supplement: Supplementary file 2 [file Data_Sheet_2.ZIP › Source data-Fig.2/Fig.2D/GSDMD-N.tif]

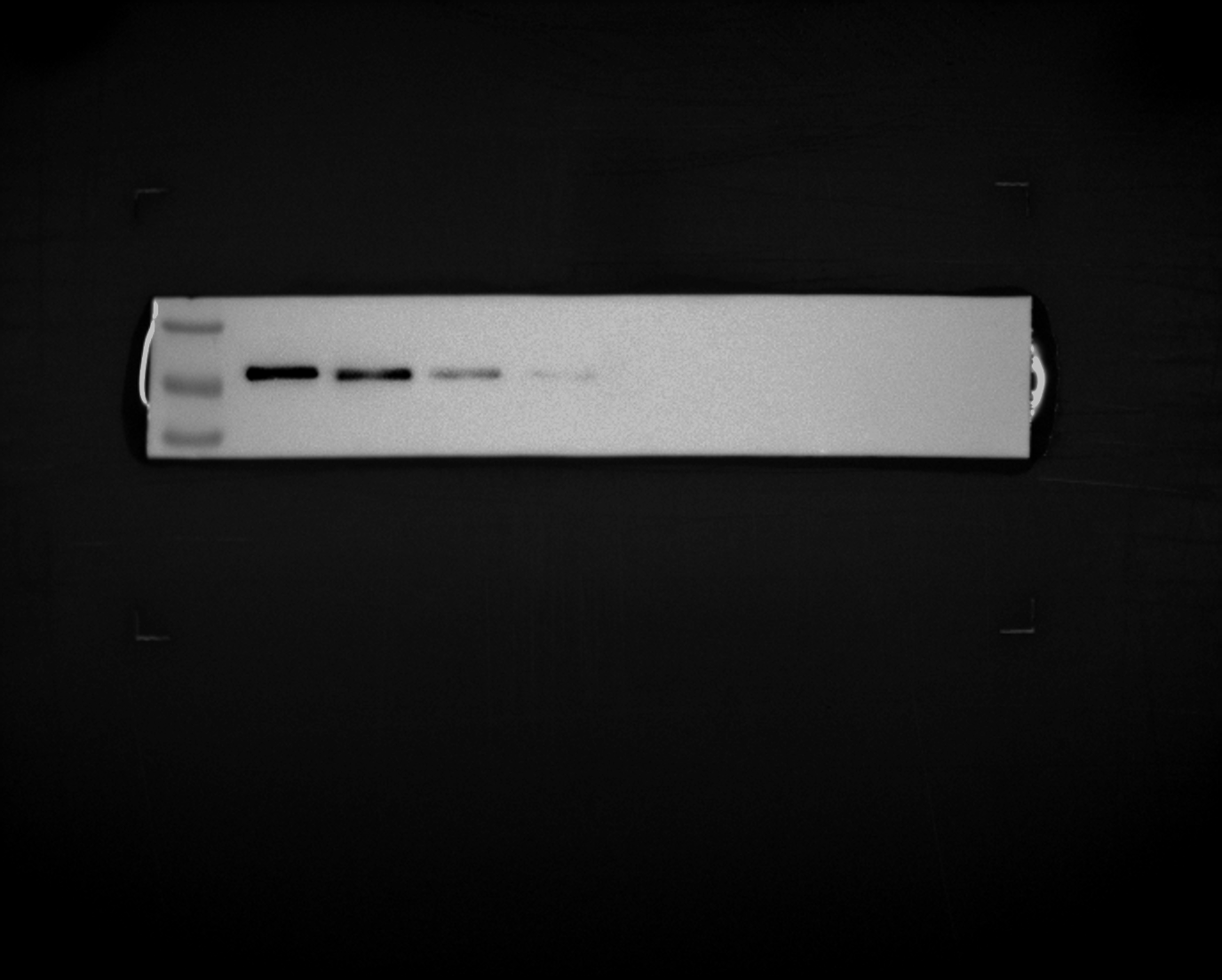

Supplement: Supplementary file 2 [file Data_Sheet_2.ZIP › Source data-Fig.2/Fig.2D/HMBOX1.tif]

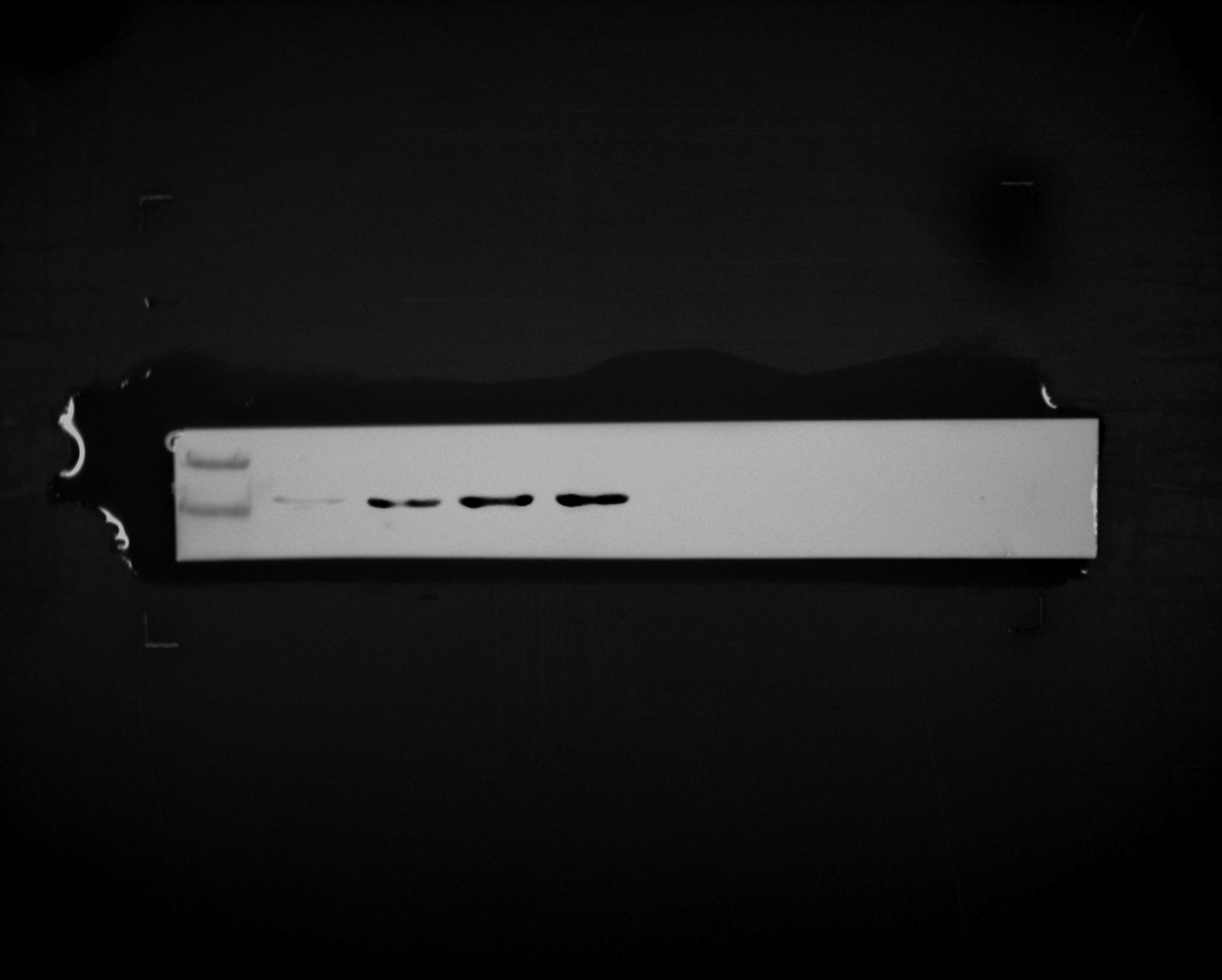

Supplement: Supplementary file 2 [file Data_Sheet_2.ZIP › Source data-Fig.2/Fig.2D/NLRP3.tif]

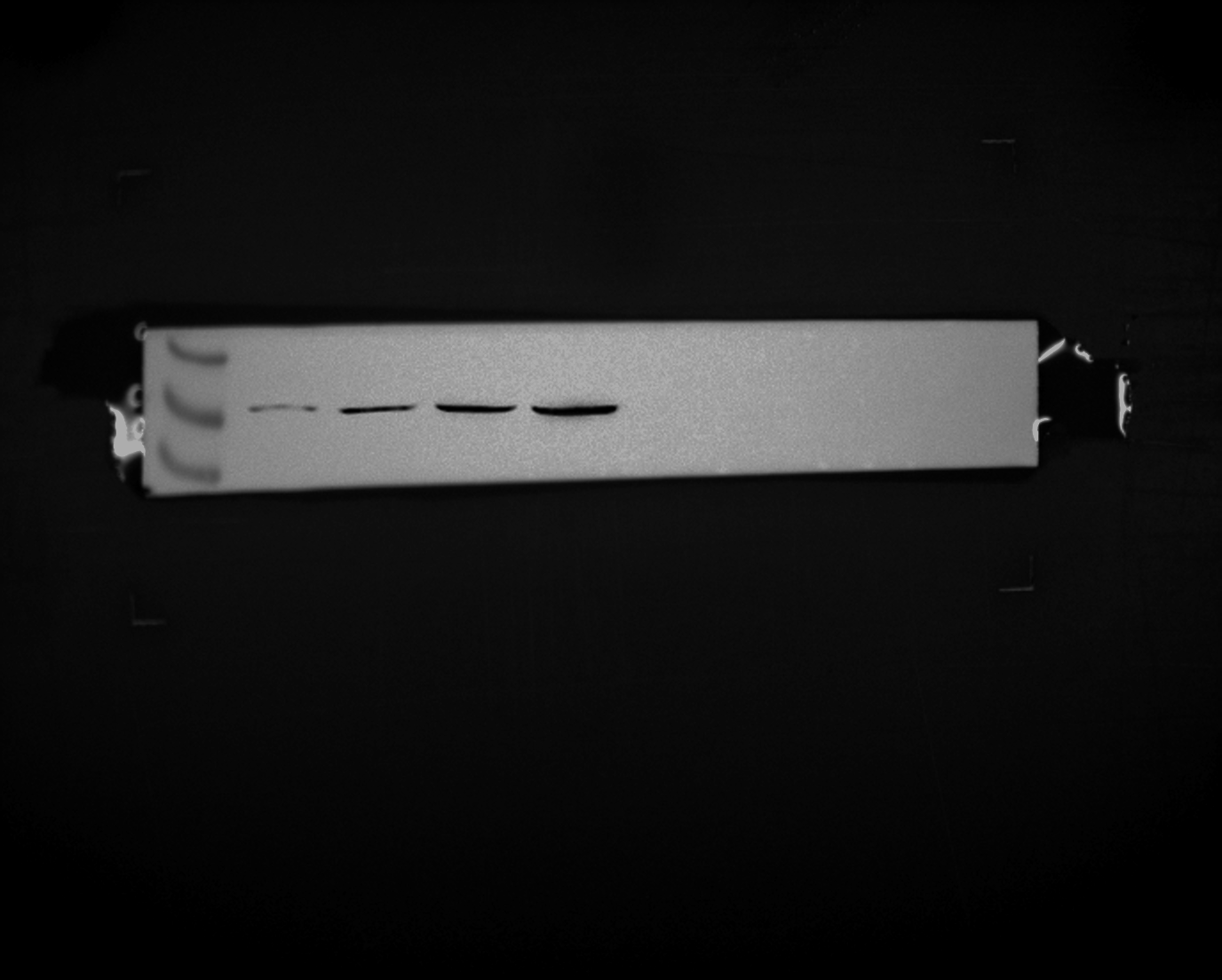

Supplement: Supplementary file 2 [file Data_Sheet_2.ZIP › Source data-Fig.2/Fig.2D/Pro-Caspase-1.tif]

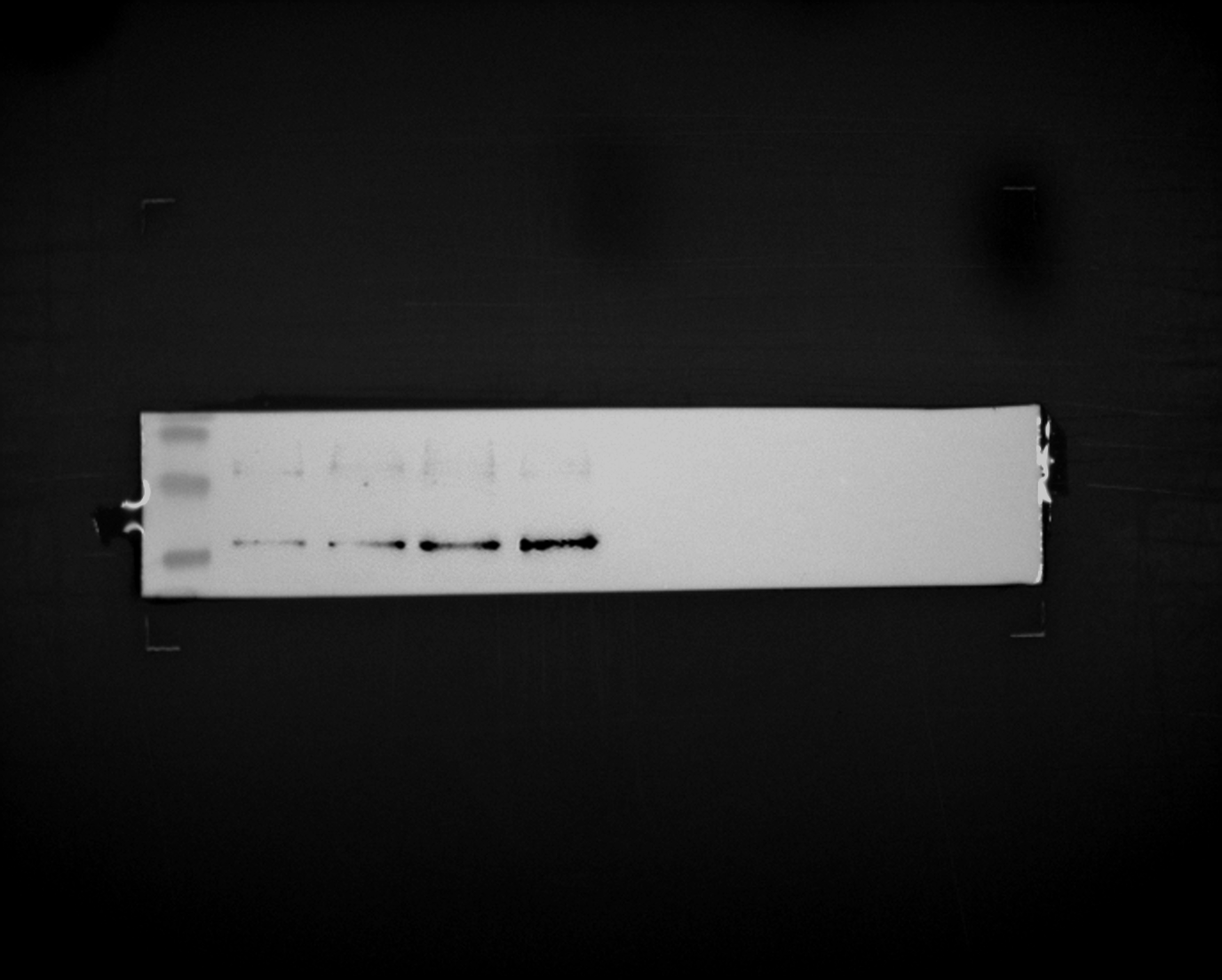

Supplement: Supplementary file 2 [file Data_Sheet_2.ZIP › Source data-Fig.2/Fig.2D/active Caspase-1.tif]

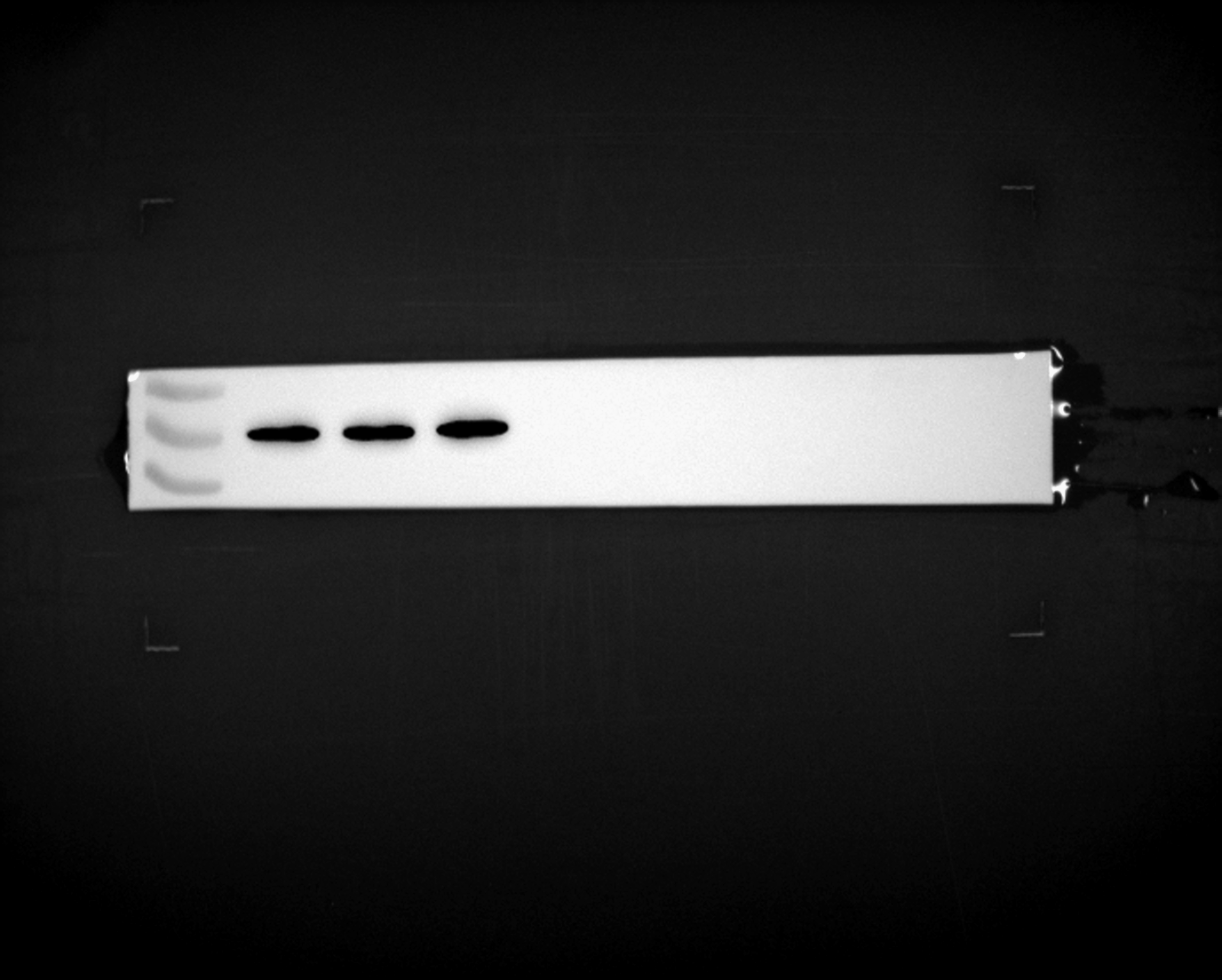

Supplement: Supplementary file 3 [file Data_Sheet_3.ZIP › Source data-Fig.3/Fig.3B/GAPDH.tif]

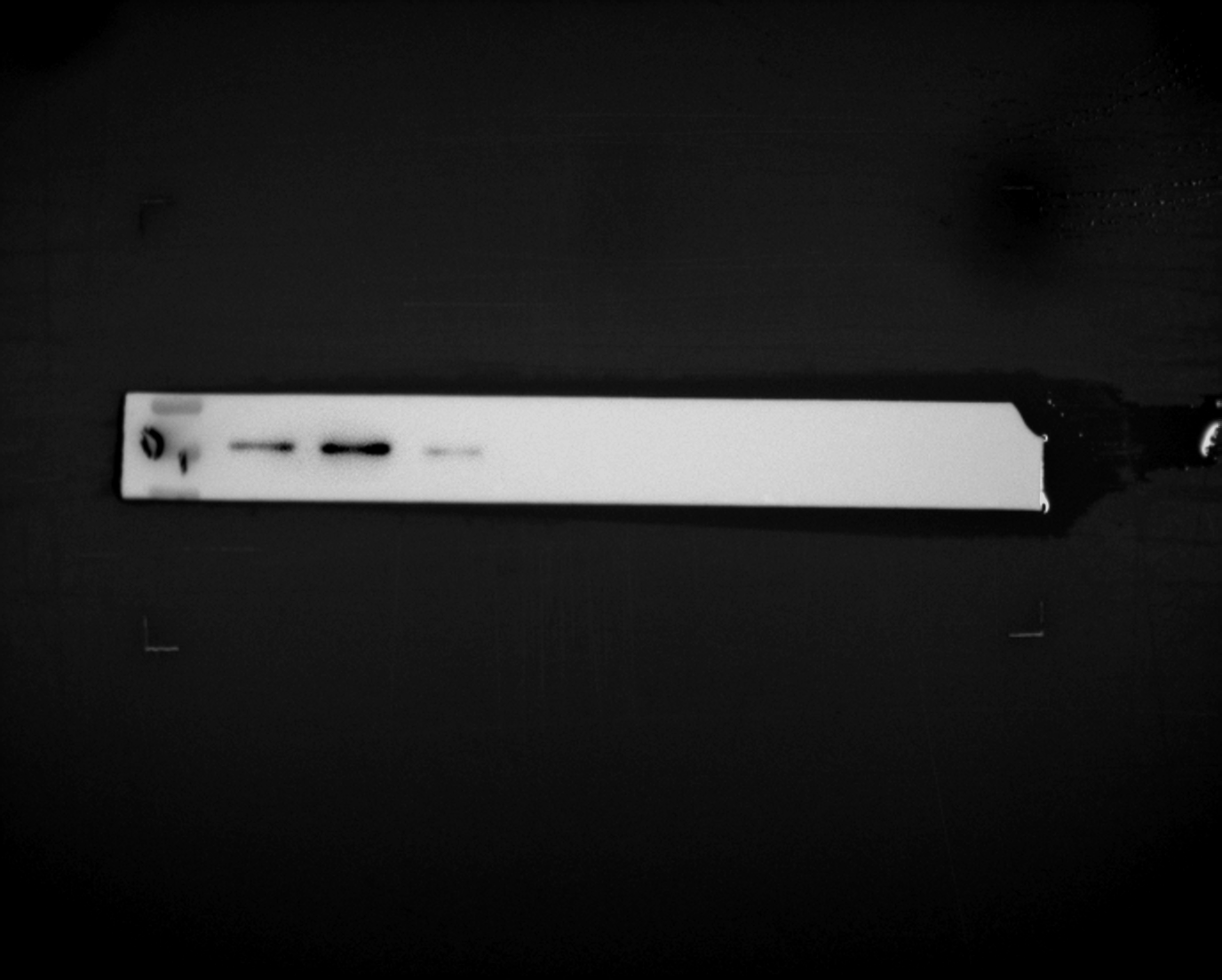

Supplement: Supplementary file 3 [file Data_Sheet_3.ZIP › Source data-Fig.3/Fig.3B/HMBOX1.tif]

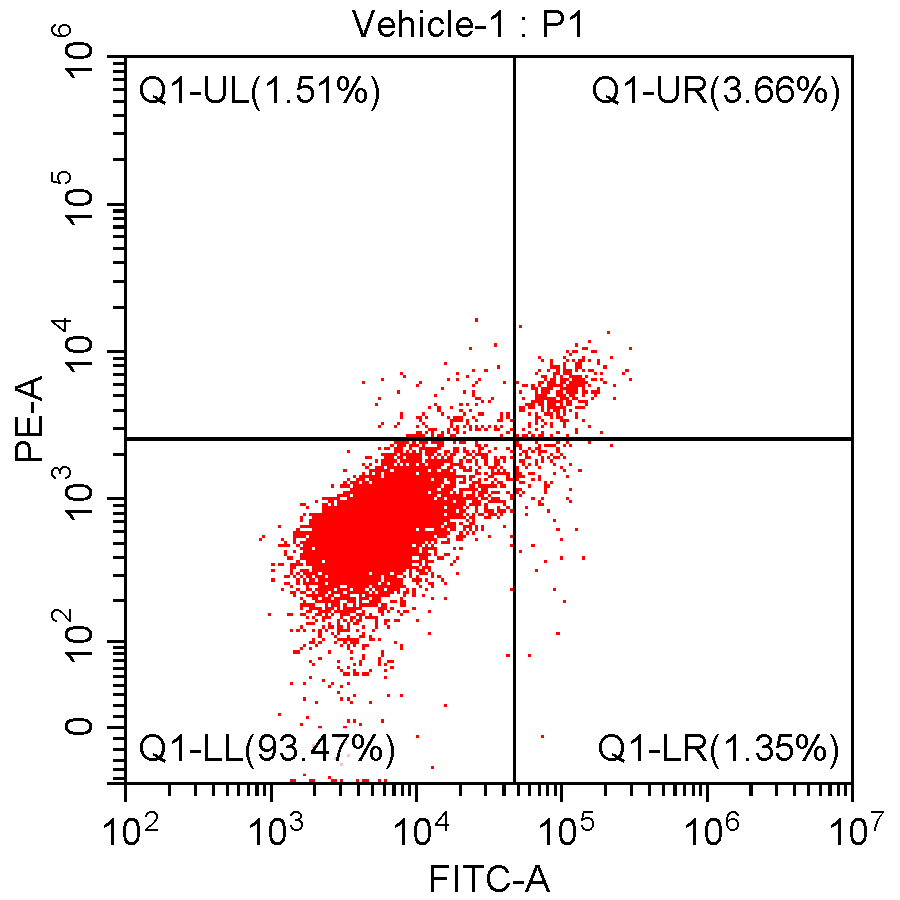

Supplement: Supplementary file 4 [file Data_Sheet_4.ZIP › Source data-Fig.4/Fig.4A/Vehicle.bmp]

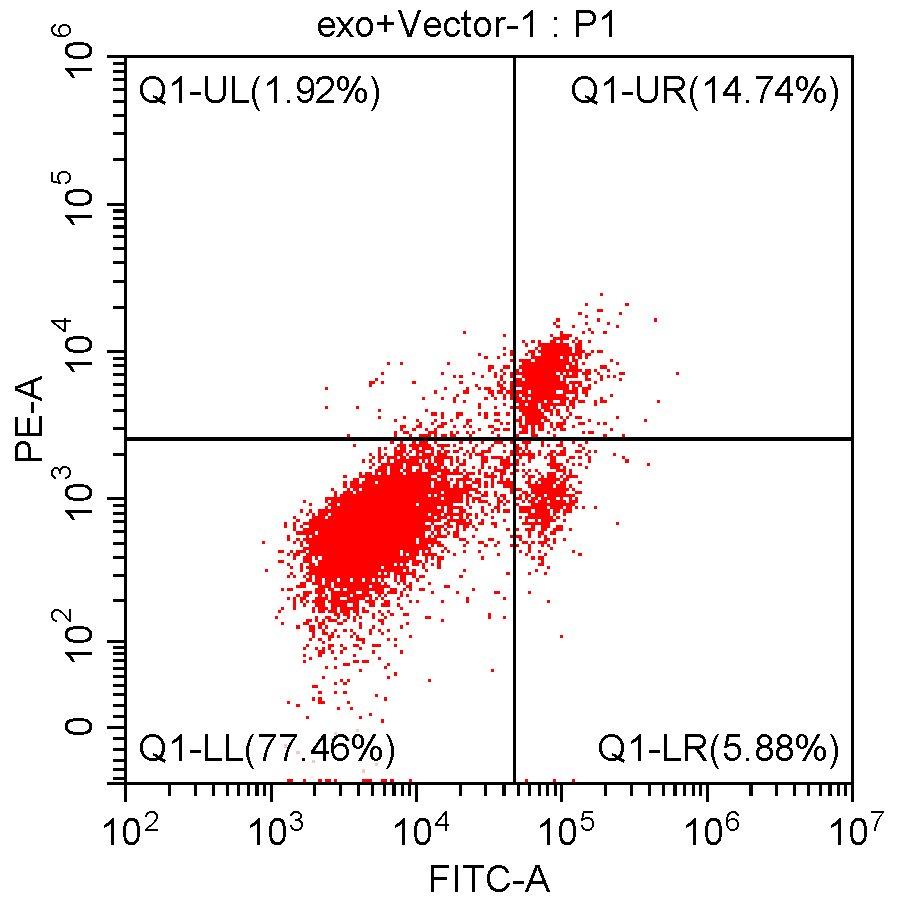

Supplement: Supplementary file 4 [file Data_Sheet_4.ZIP › Source data-Fig.4/Fig.4A/exo+Vector.bmp]

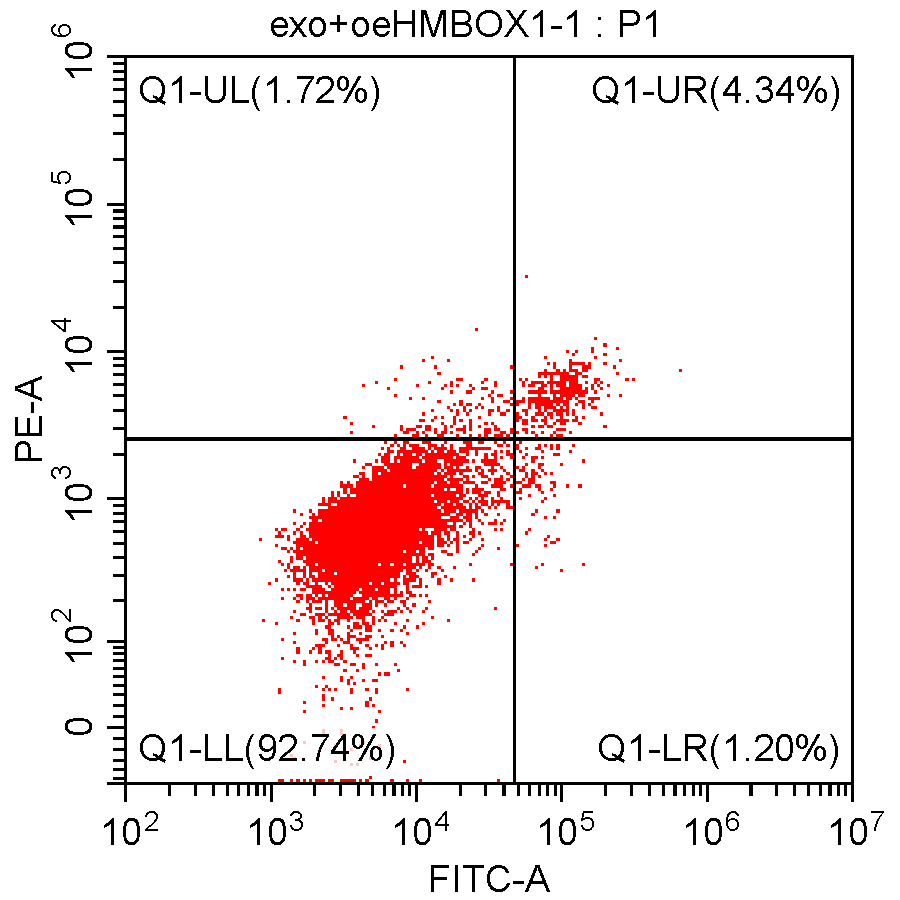

Supplement: Supplementary file 4 [file Data_Sheet_4.ZIP › Source data-Fig.4/Fig.4A/exo+oeHMBOX1.bmp]

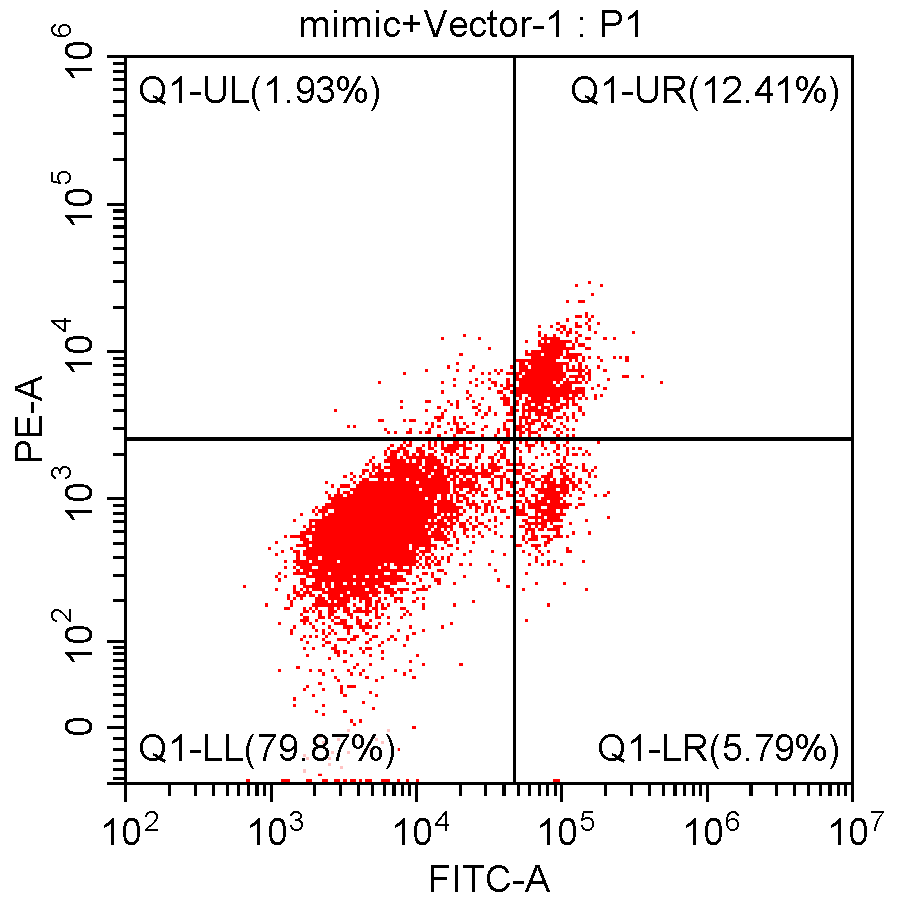

Supplement: Supplementary file 4 [file Data_Sheet_4.ZIP › Source data-Fig.4/Fig.4A/mimic+Vector.bmp]

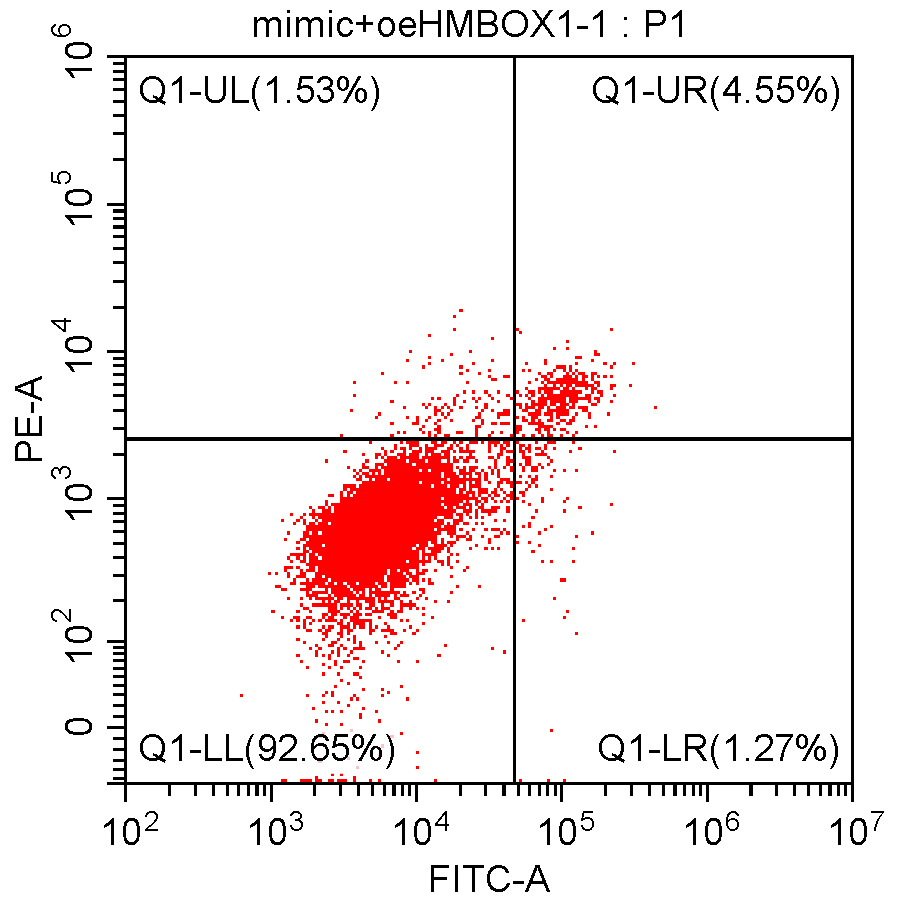

Supplement: Supplementary file 4 [file Data_Sheet_4.ZIP › Source data-Fig.4/Fig.4A/mimic+oeHMBOX1.bmp]

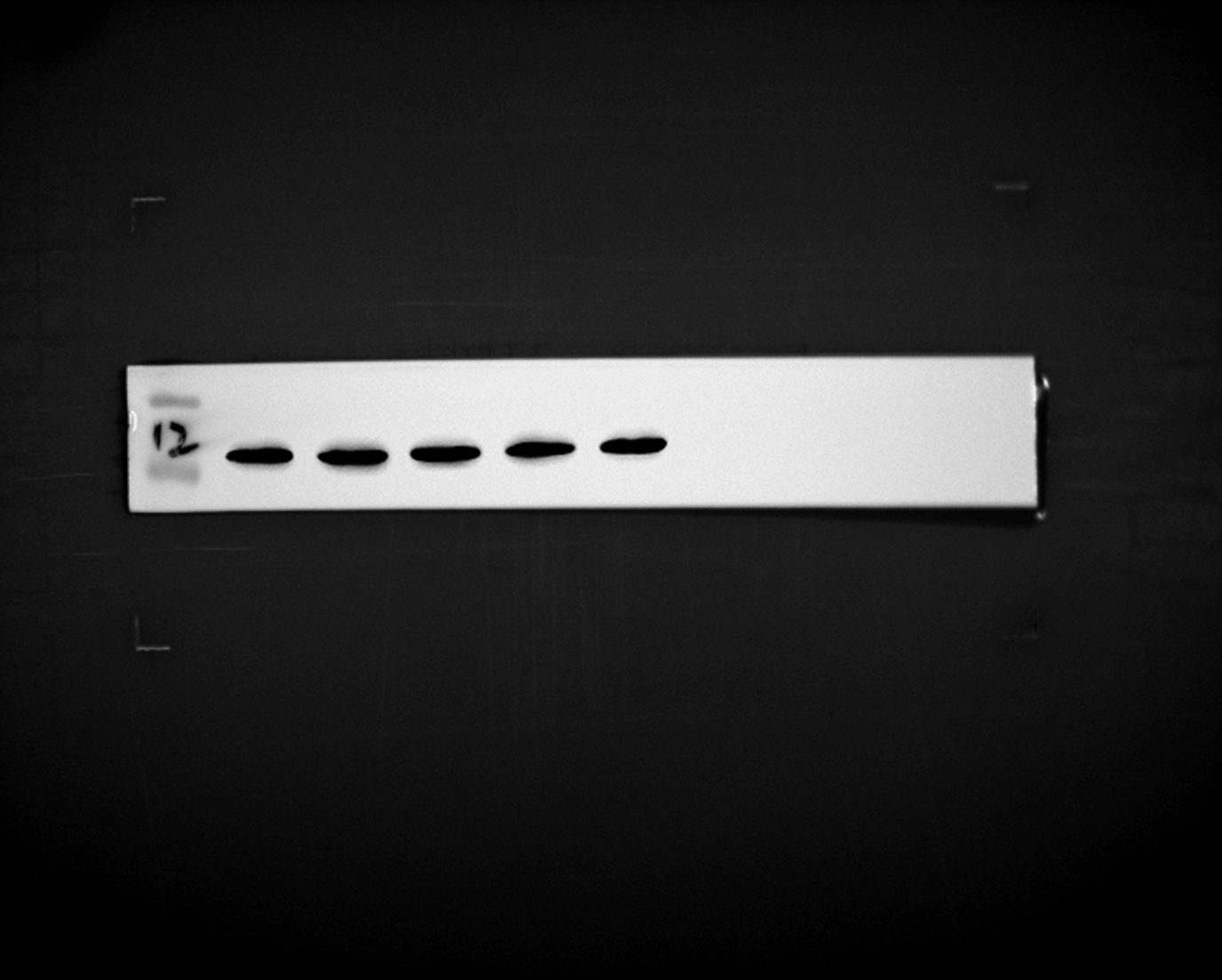

Supplement: Supplementary file 4 [file Data_Sheet_4.ZIP › Source data-Fig.4/Fig.4C/GADPH.tif]

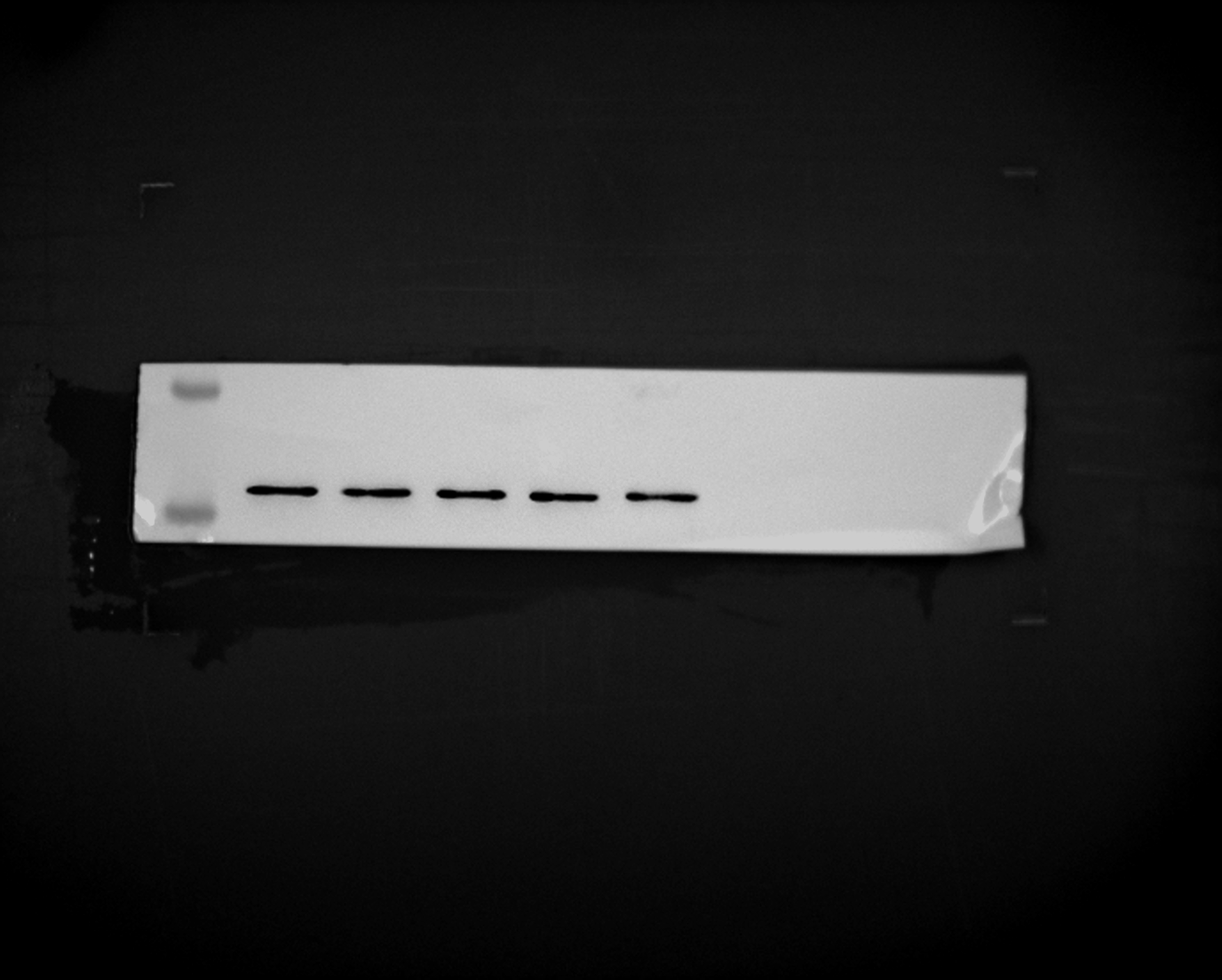

Supplement: Supplementary file 4 [file Data_Sheet_4.ZIP › Source data-Fig.4/Fig.4C/H3.tif]

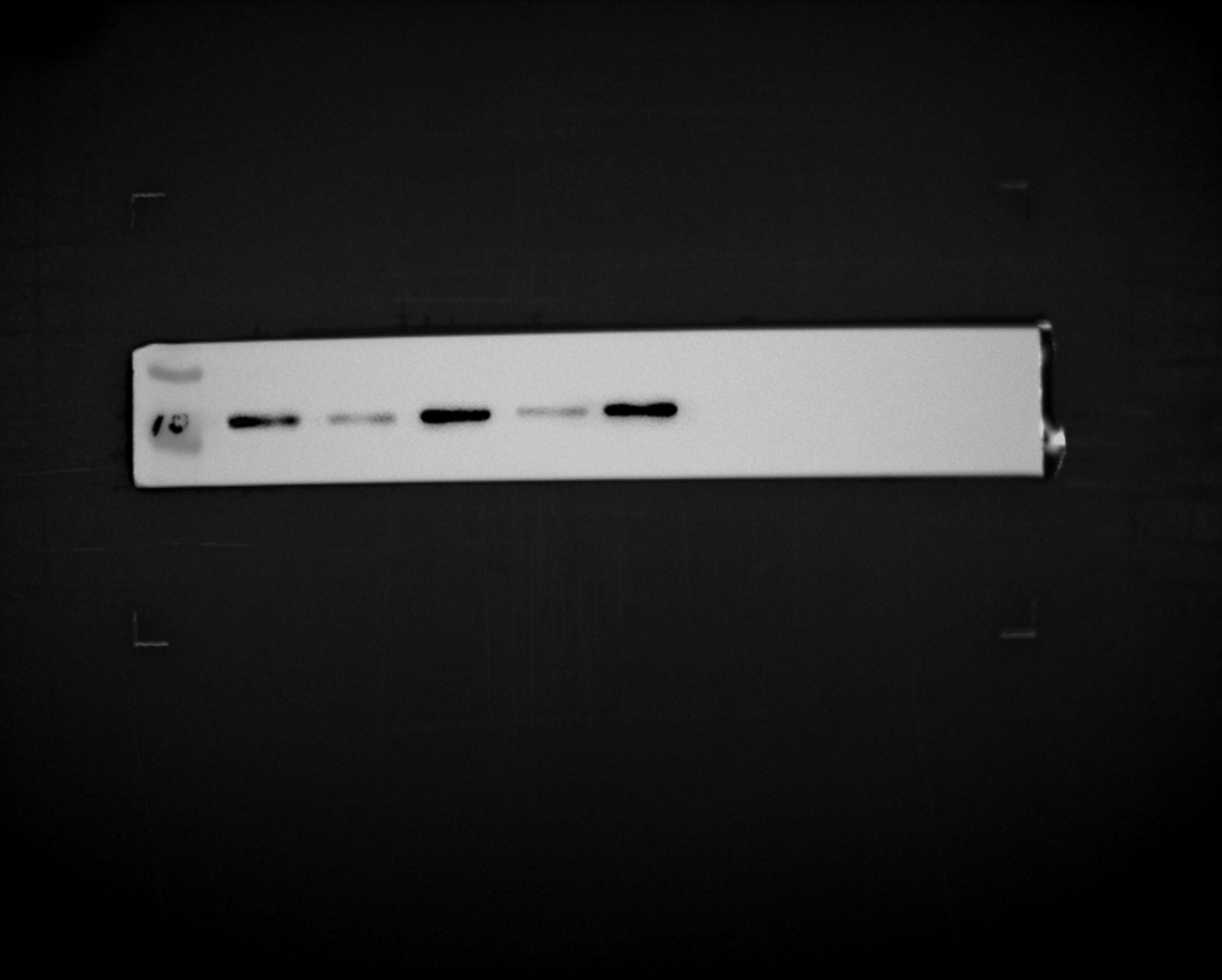

Supplement: Supplementary file 4 [file Data_Sheet_4.ZIP › Source data-Fig.4/Fig.4C/HMBOX1.tif]

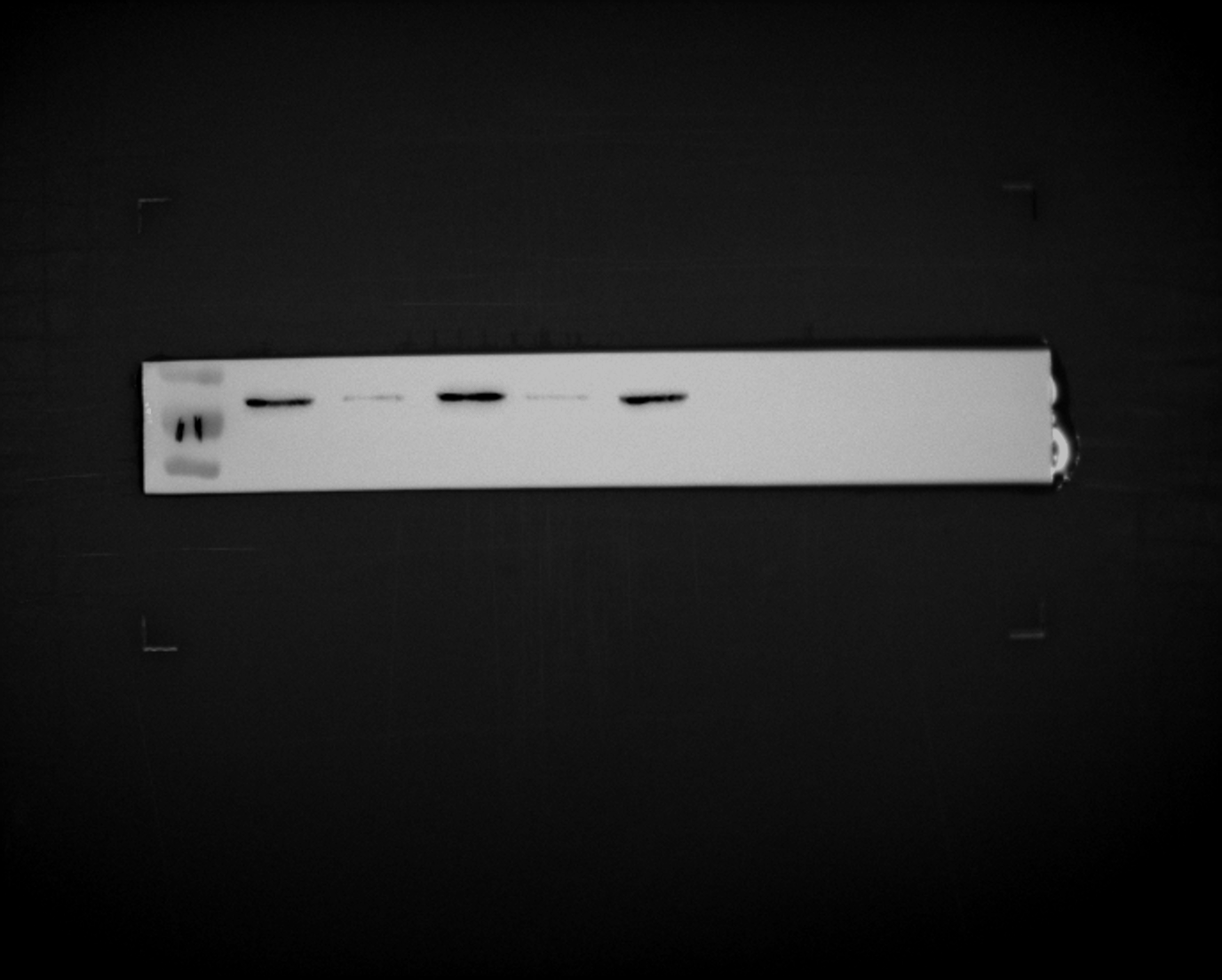

Supplement: Supplementary file 4 [file Data_Sheet_4.ZIP › Source data-Fig.4/Fig.4C/NF-a╩B(cyto).tif]

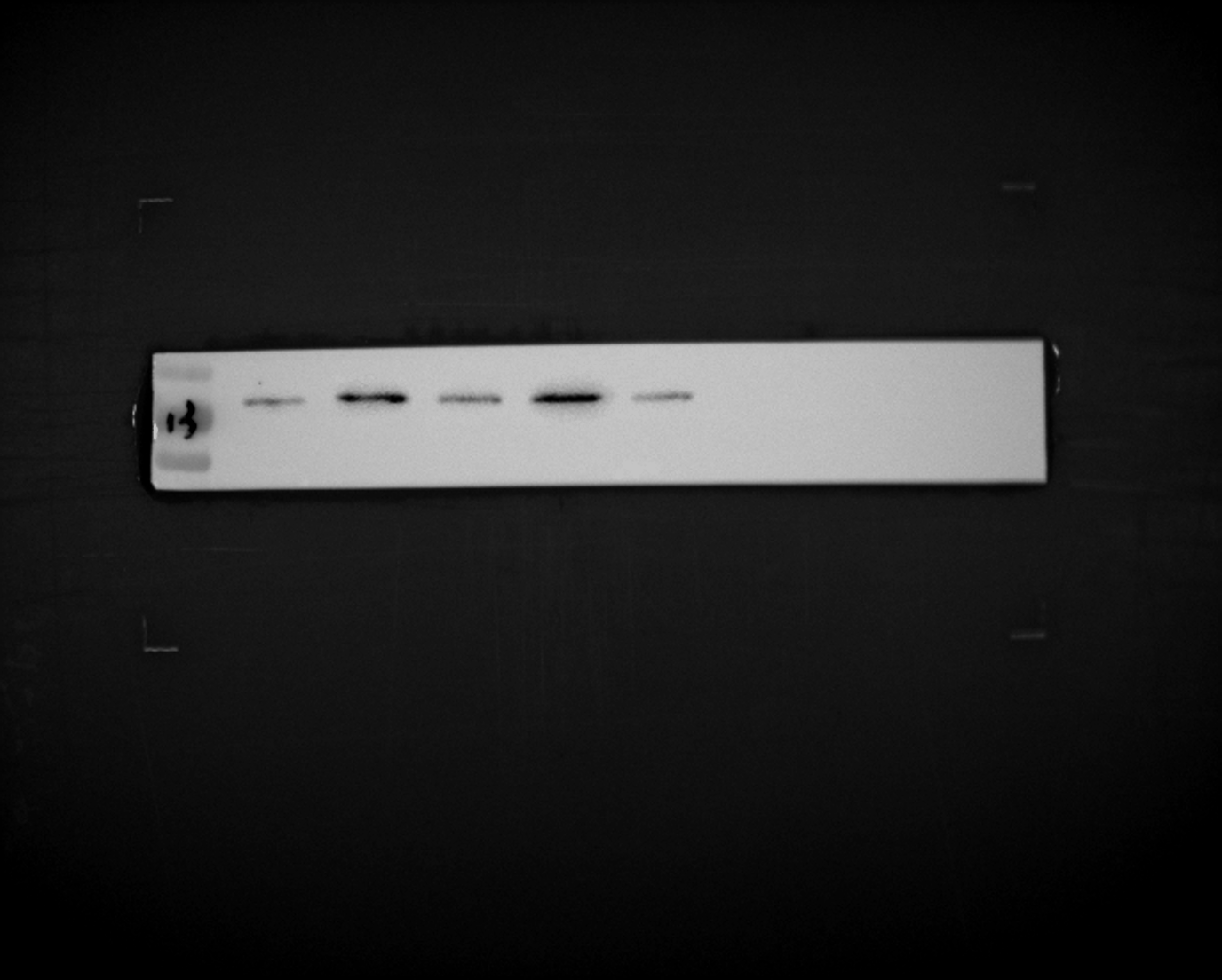

Supplement: Supplementary file 4 [file Data_Sheet_4.ZIP › Source data-Fig.4/Fig.4C/NF-a╩B(nuclear).tif]

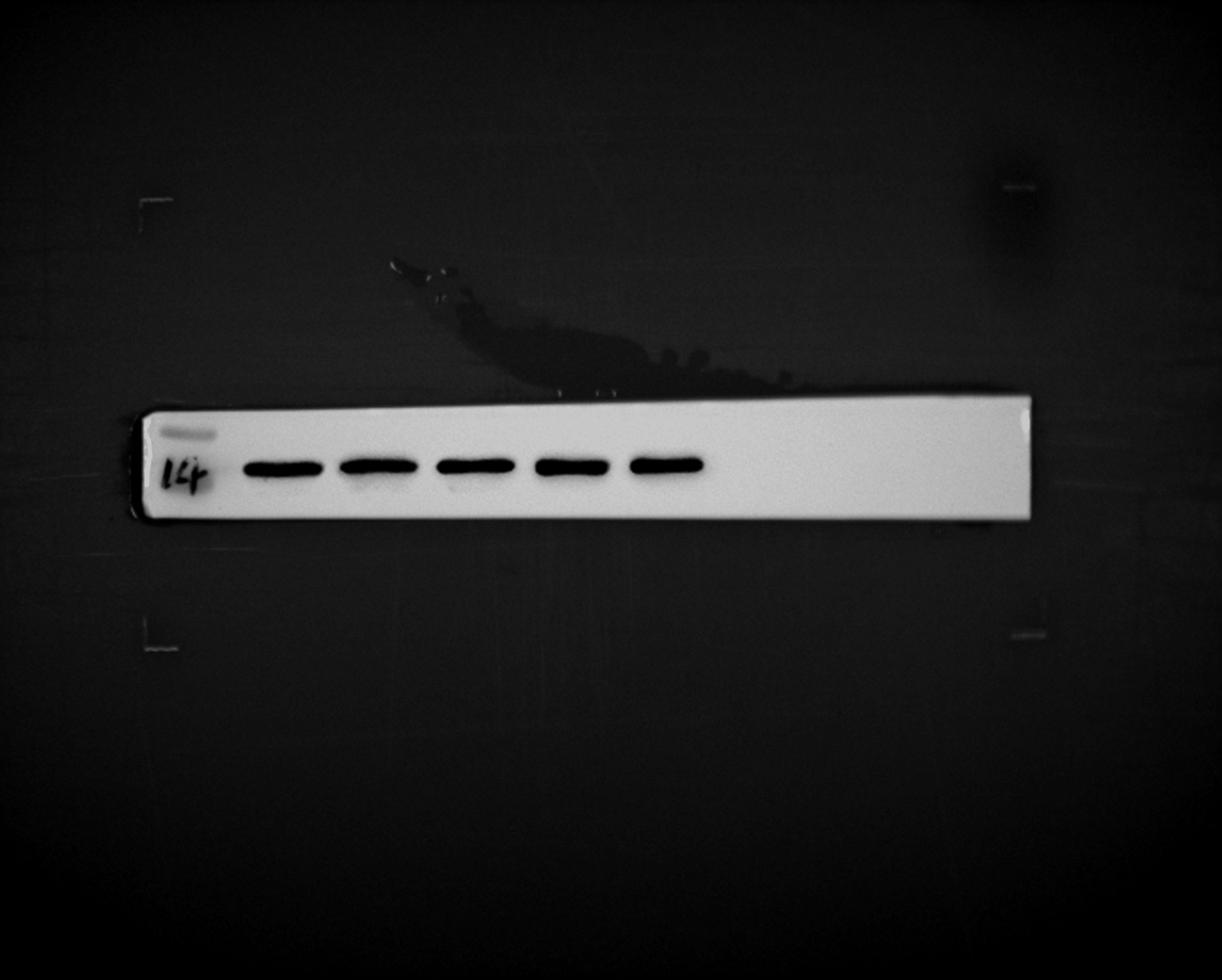

Supplement: Supplementary file 4 [file Data_Sheet_4.ZIP › Source data-Fig.4/Fig.4D/GAPDH.tif]

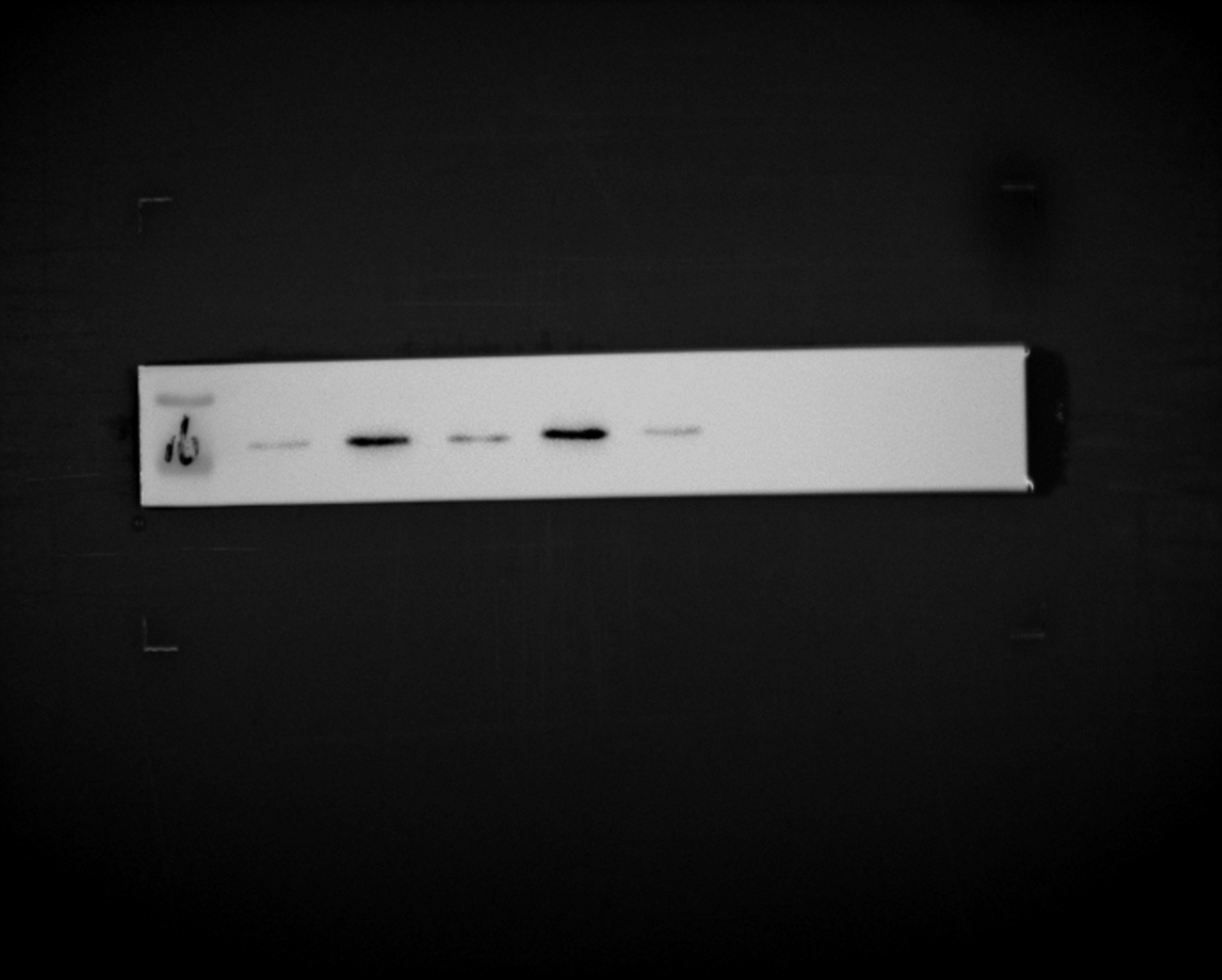

Supplement: Supplementary file 4 [file Data_Sheet_4.ZIP › Source data-Fig.4/Fig.4D/GSDMD-N.tif]

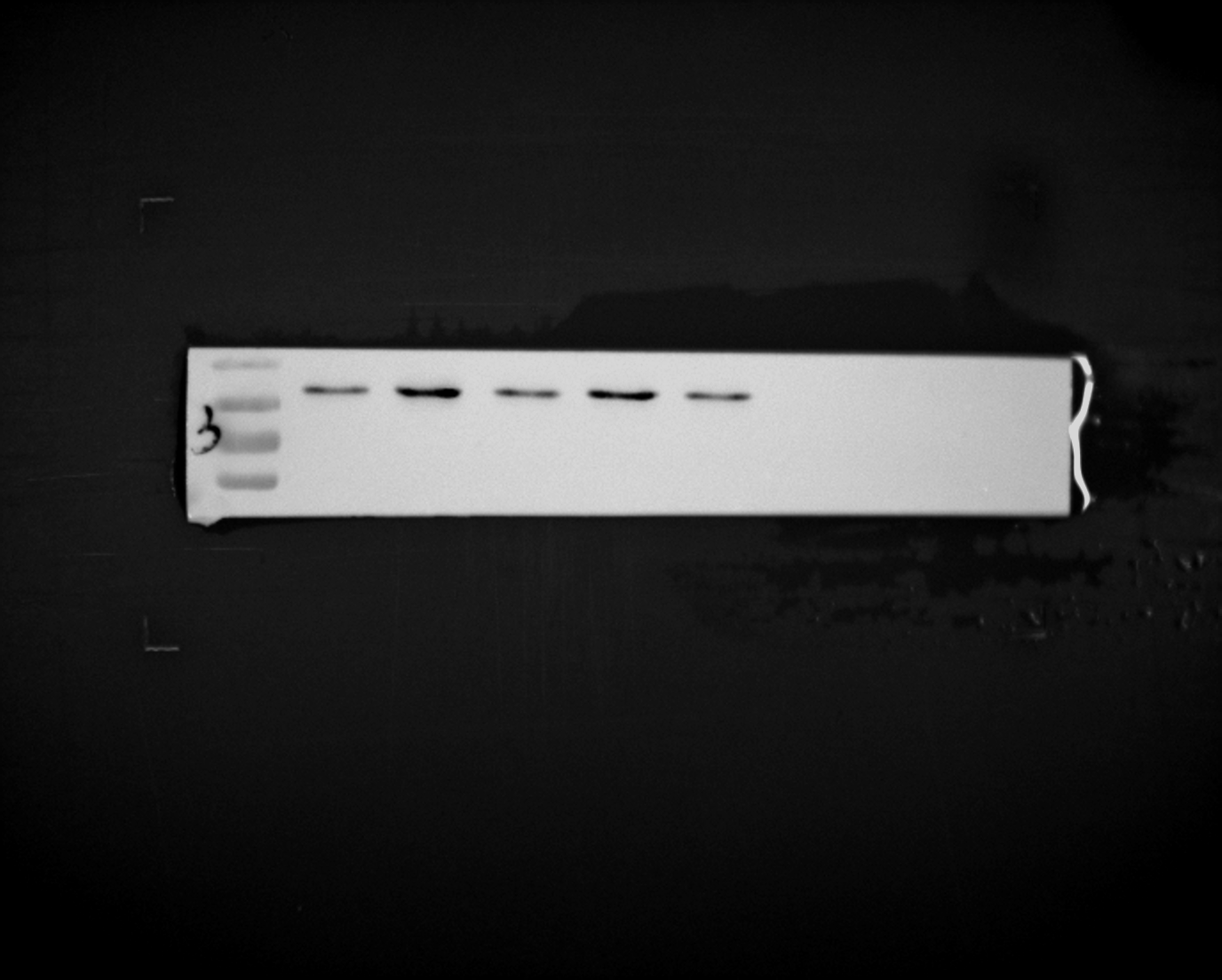

Supplement: Supplementary file 4 [file Data_Sheet_4.ZIP › Source data-Fig.4/Fig.4D/NLRP3.tif]

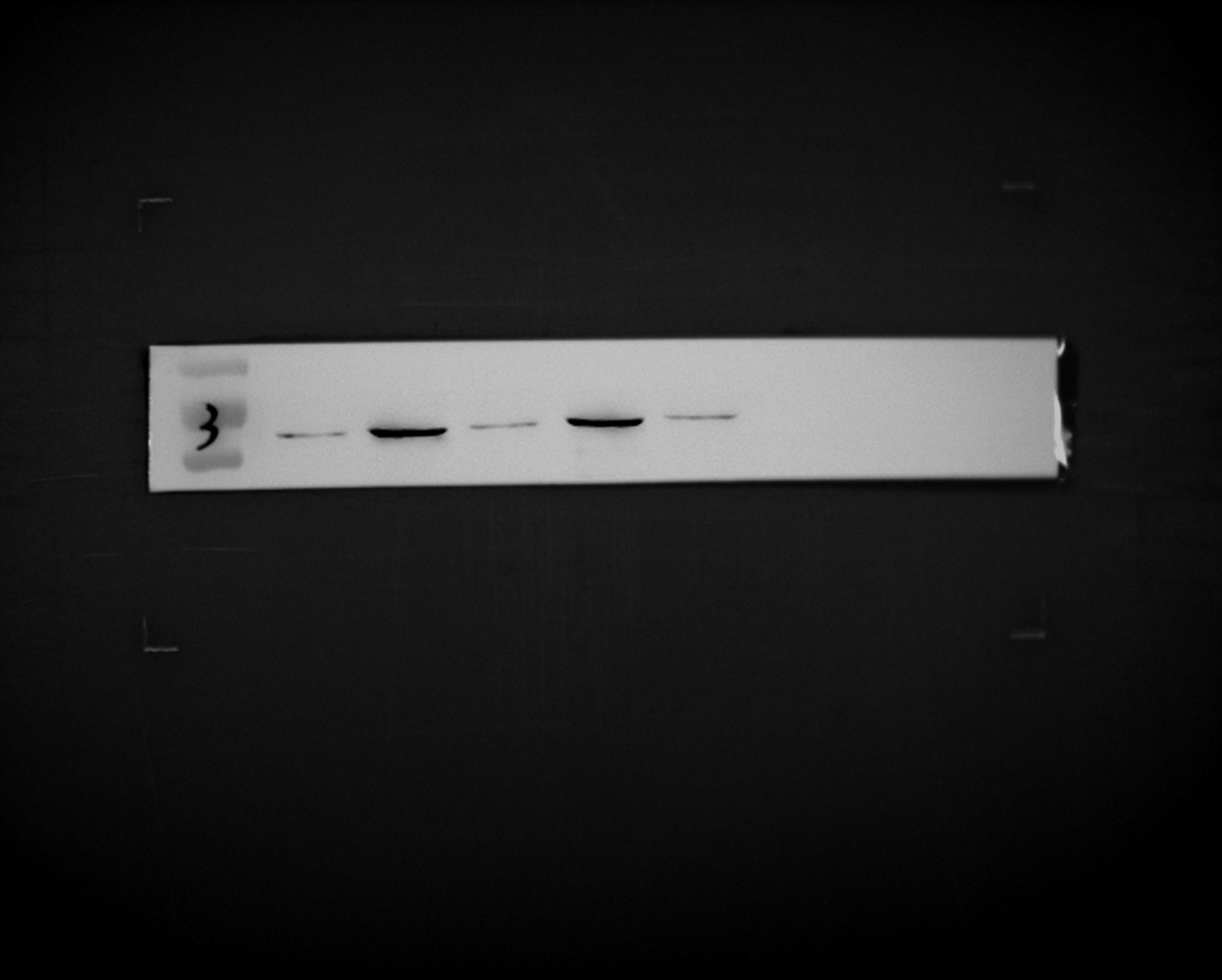

Supplement: Supplementary file 4 [file Data_Sheet_4.ZIP › Source data-Fig.4/Fig.4D/Pro-Caspase-1.tif]

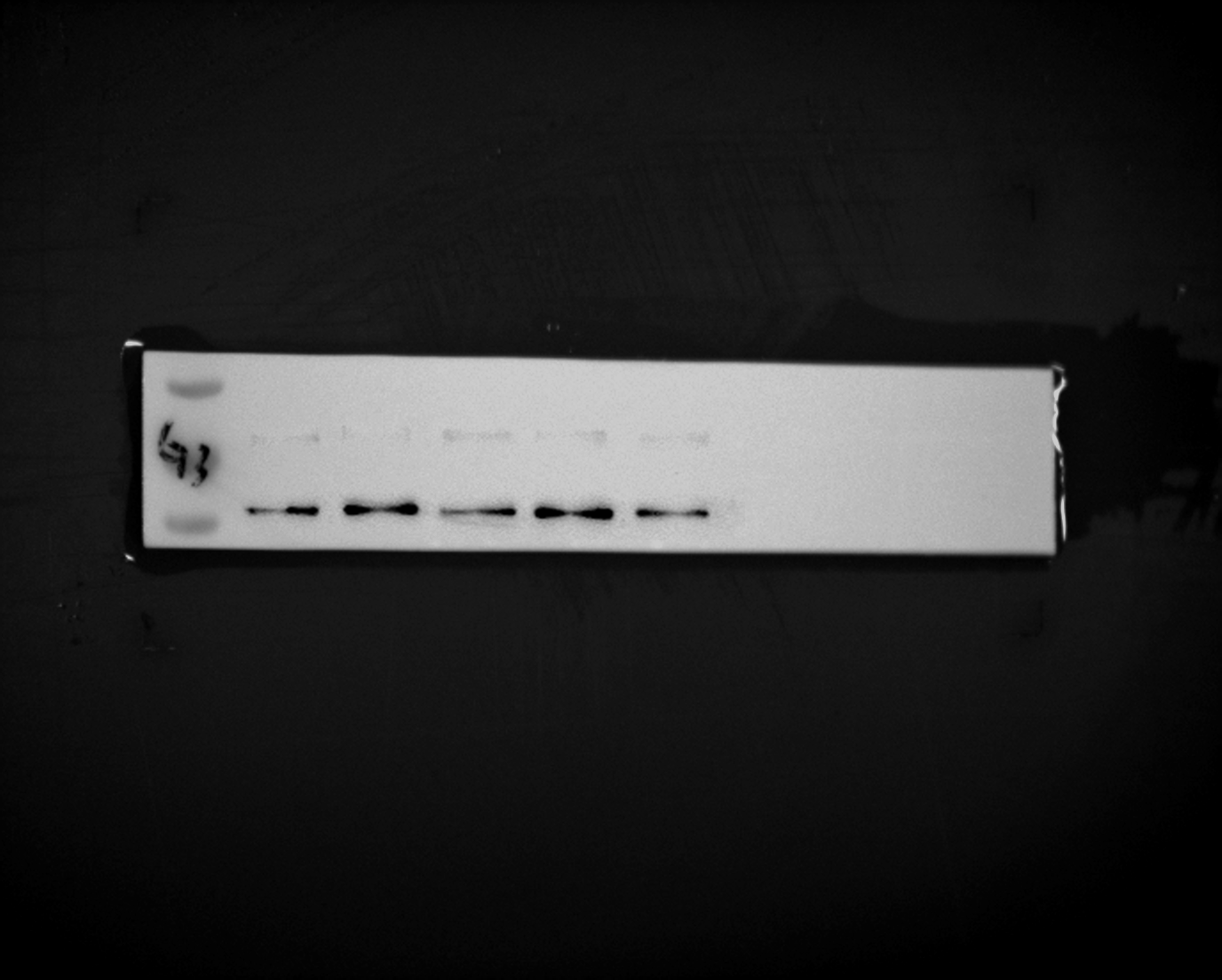

Supplement: Supplementary file 4 [file Data_Sheet_4.ZIP › Source data-Fig.4/Fig.4D/active Caspase-1.tif]

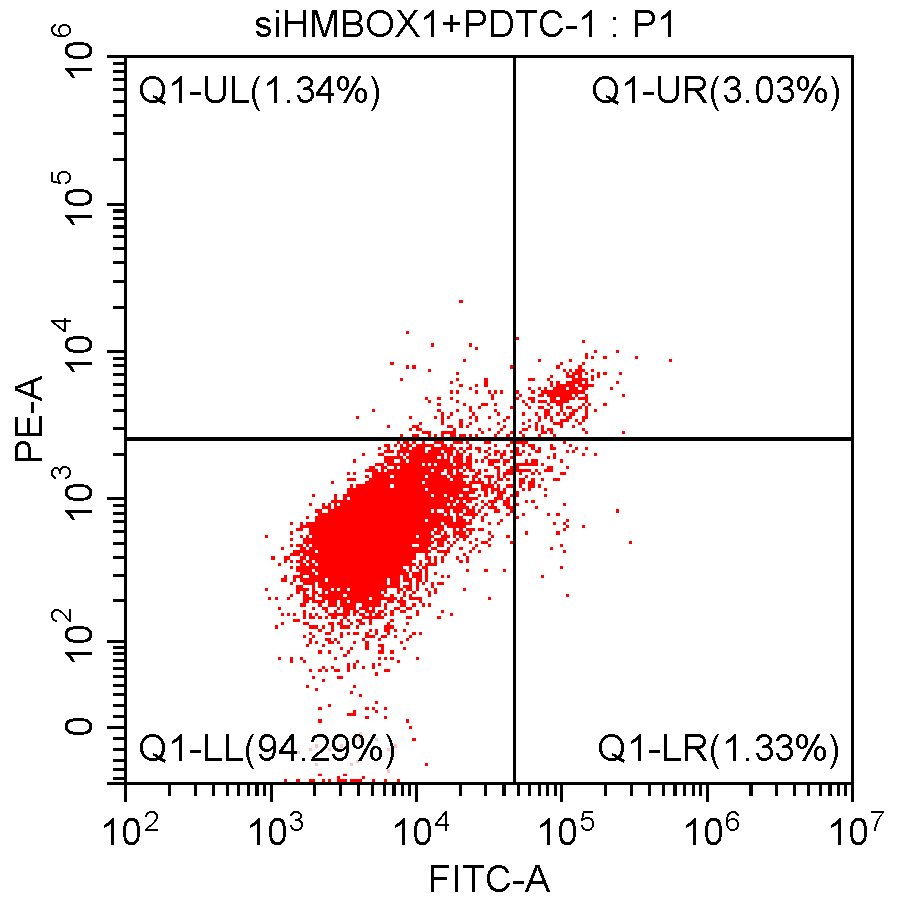

Supplement: Supplementary file 5 [file Data_Sheet_5.ZIP › Source data-Fig.5/Fig.5A/siHMBOX1+PDTC.bmp]

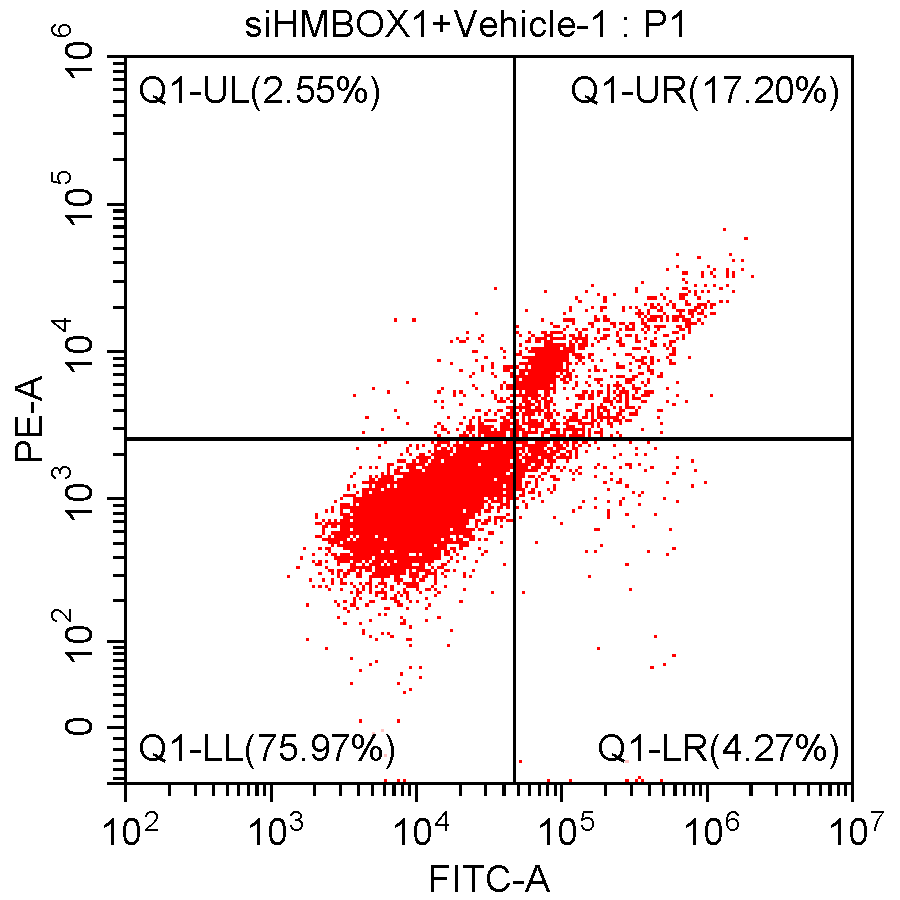

Supplement: Supplementary file 5 [file Data_Sheet_5.ZIP › Source data-Fig.5/Fig.5A/siHMBOX1+Vehicle.bmp]

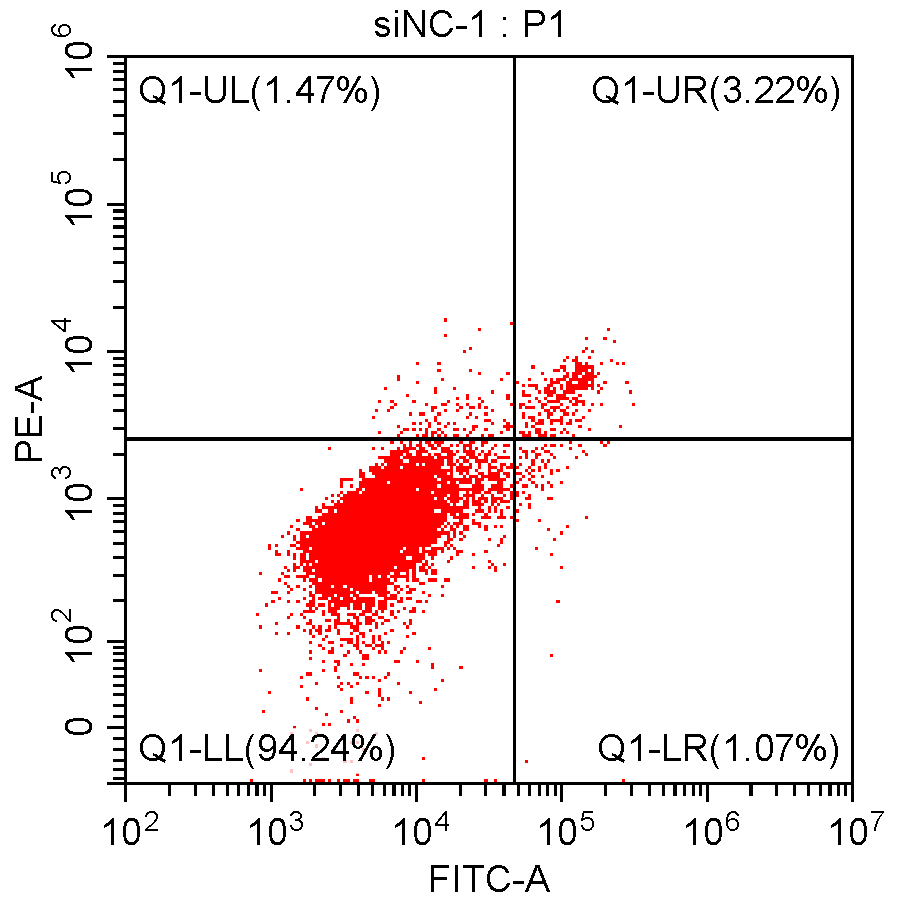

Supplement: Supplementary file 5 [file Data_Sheet_5.ZIP › Source data-Fig.5/Fig.5A/siNC.bmp]

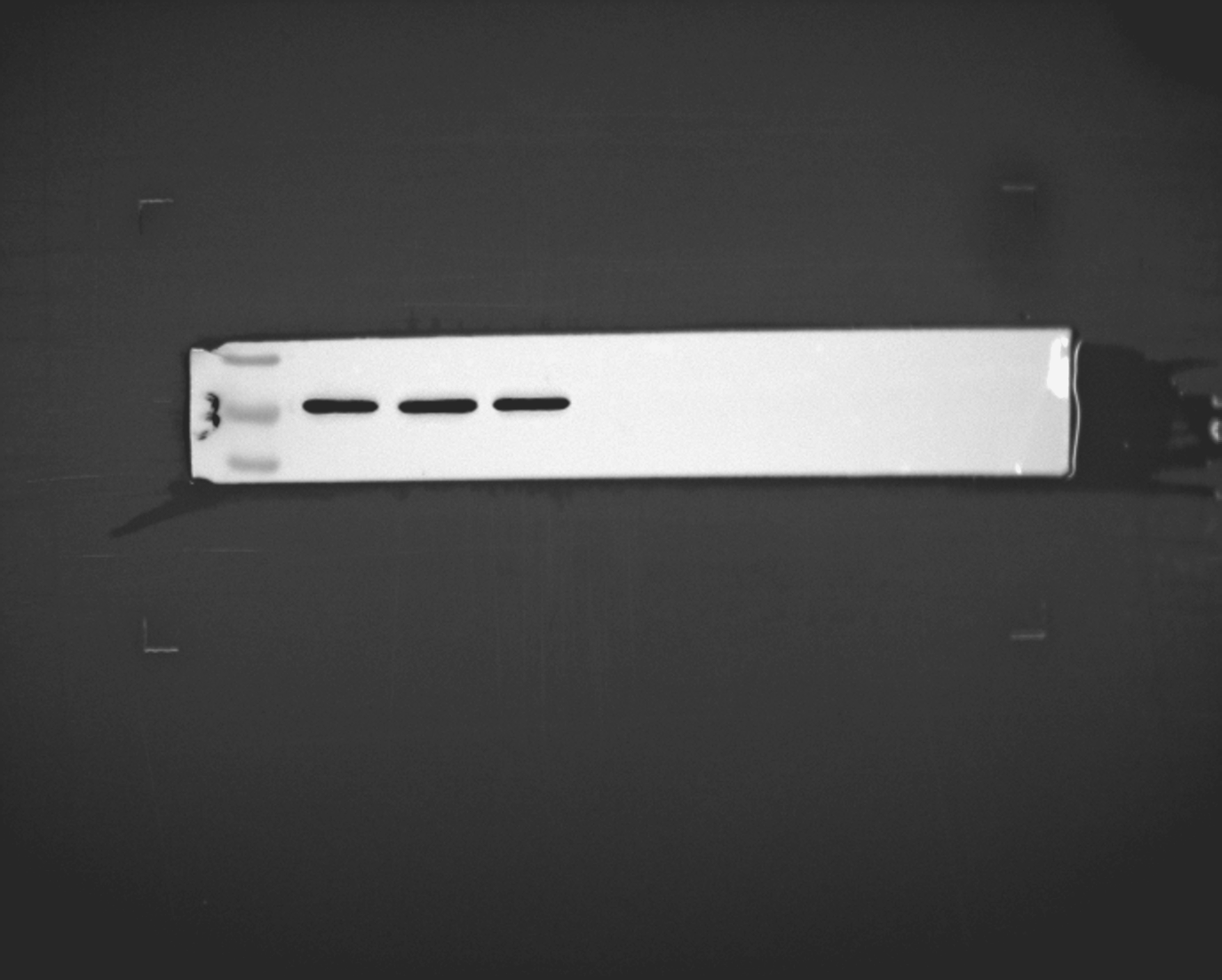

Supplement: Supplementary file 5 [file Data_Sheet_5.ZIP › Source data-Fig.5/Fig.5C/GAPDH1.tif]

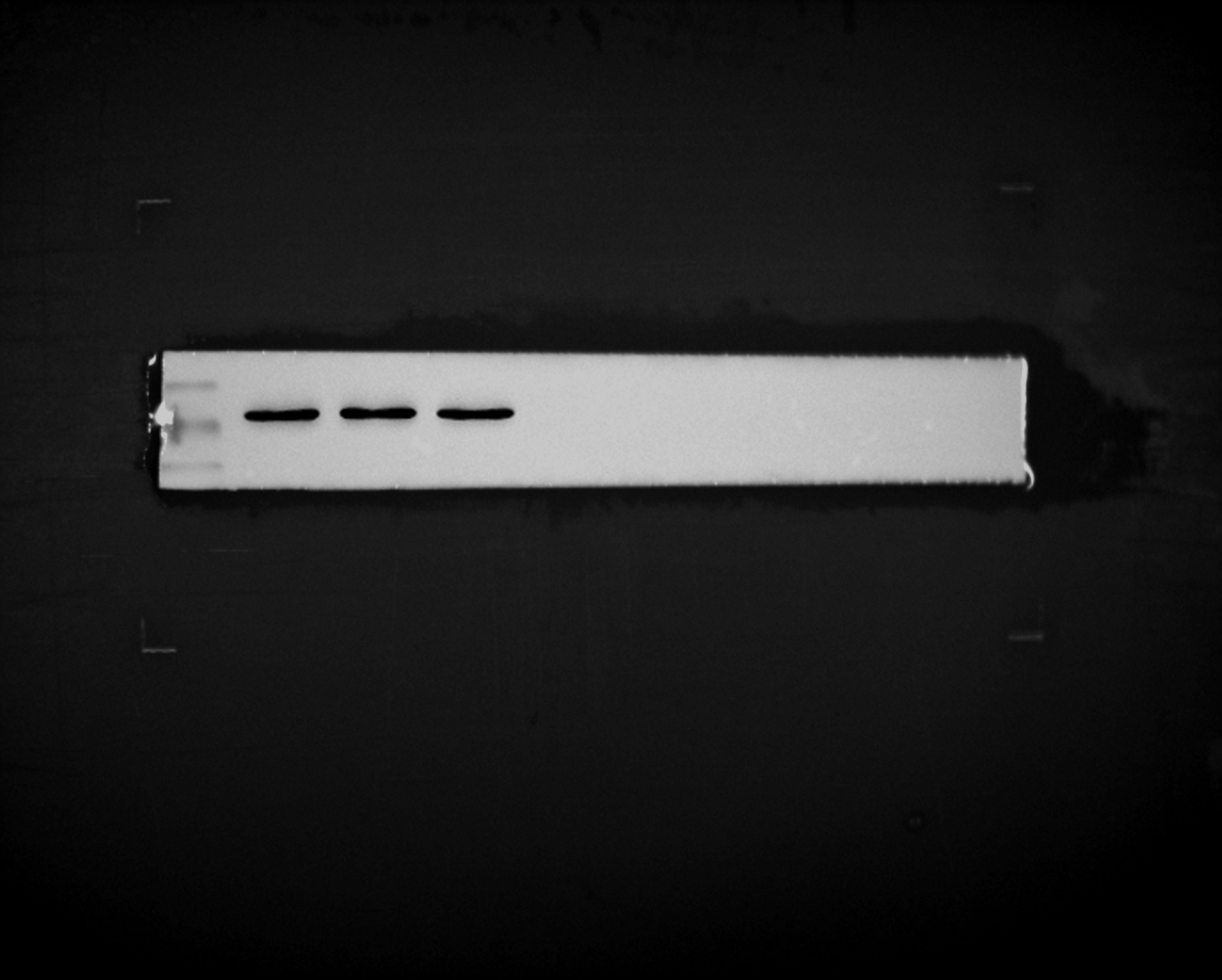

Supplement: Supplementary file 5 [file Data_Sheet_5.ZIP › Source data-Fig.5/Fig.5C/H3.tif]

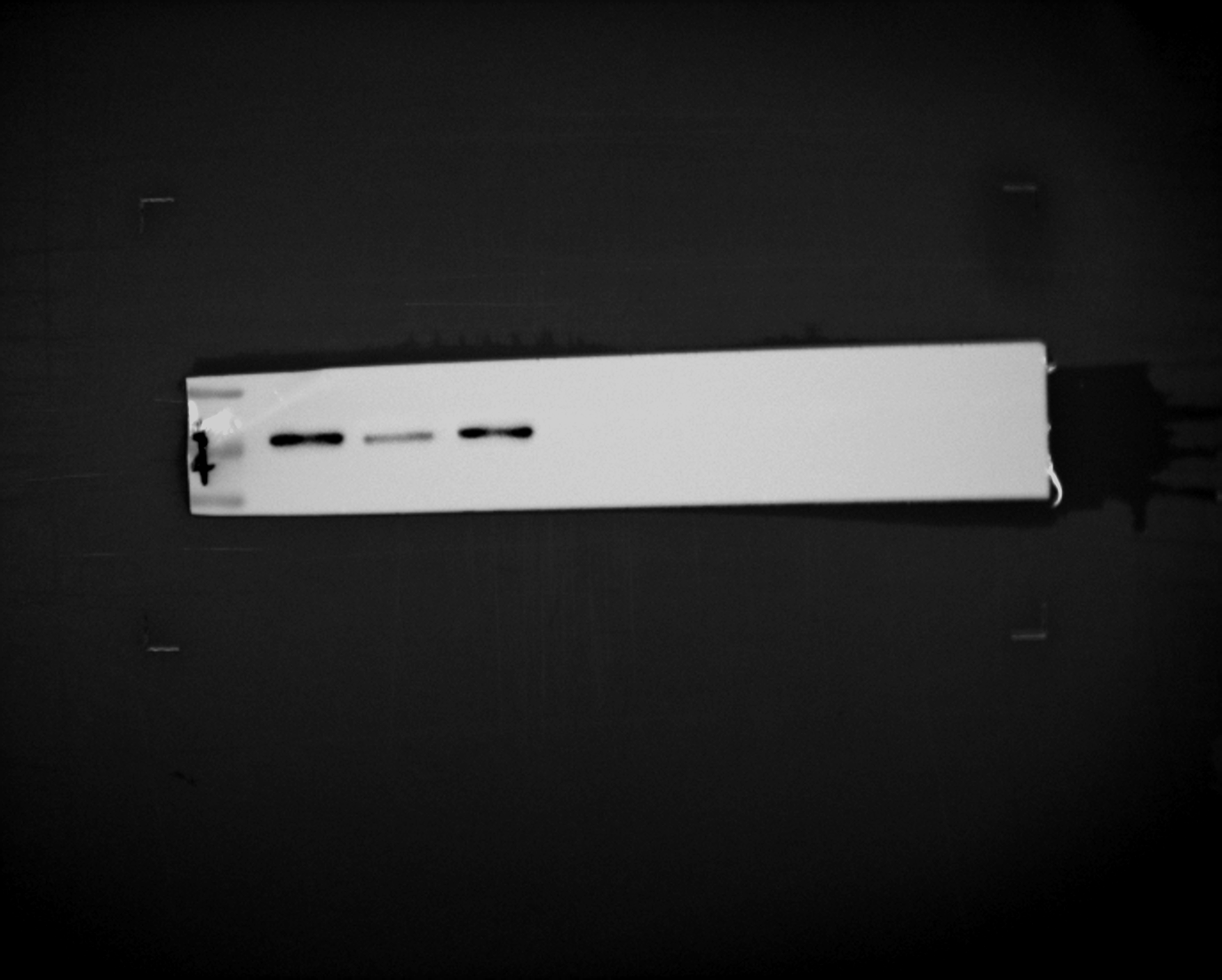

Supplement: Supplementary file 5 [file Data_Sheet_5.ZIP › Source data-Fig.5/Fig.5C/HMBOX1.tif]

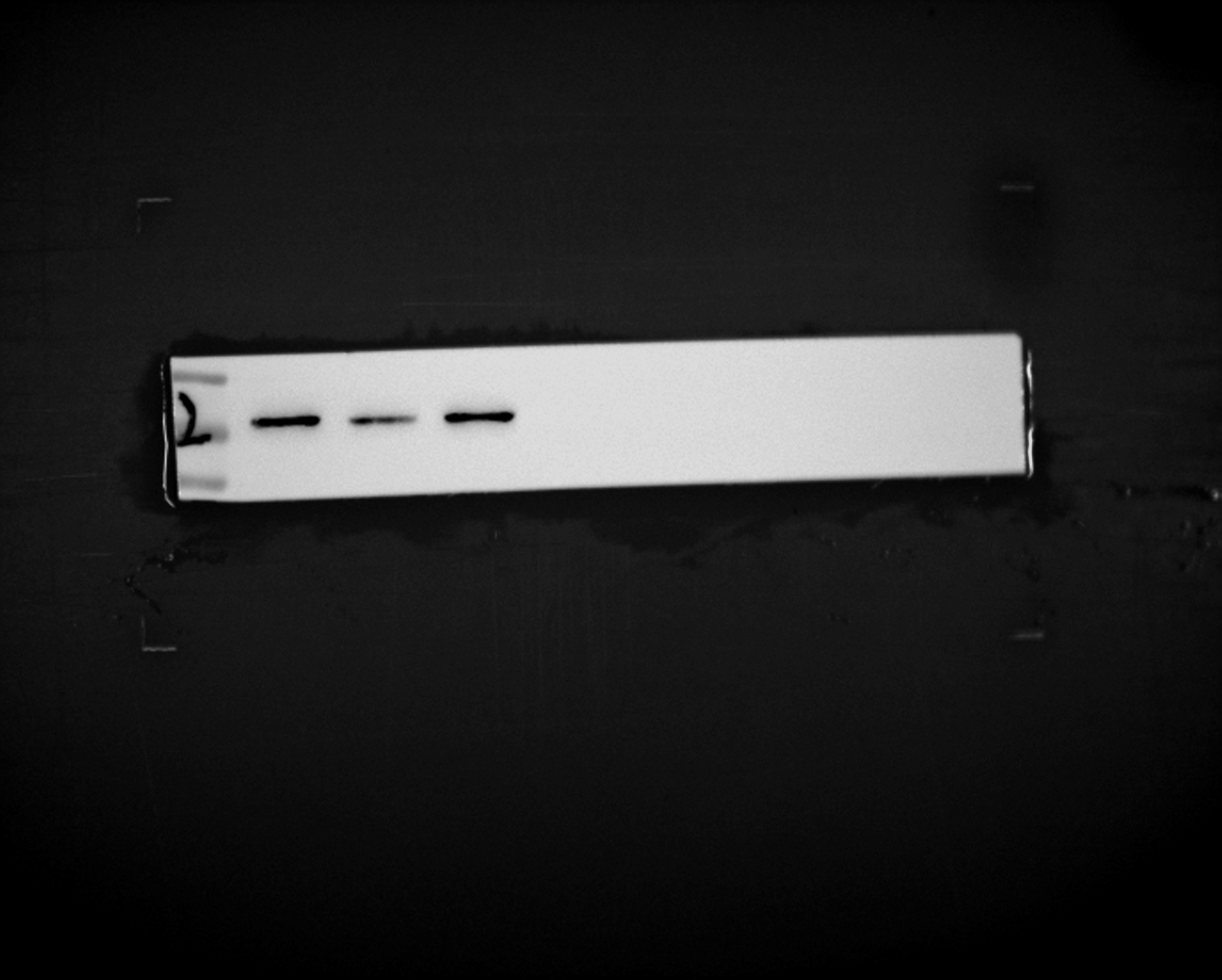

Supplement: Supplementary file 5 [file Data_Sheet_5.ZIP › Source data-Fig.5/Fig.5C/NF-a╩B(cyto).tif]

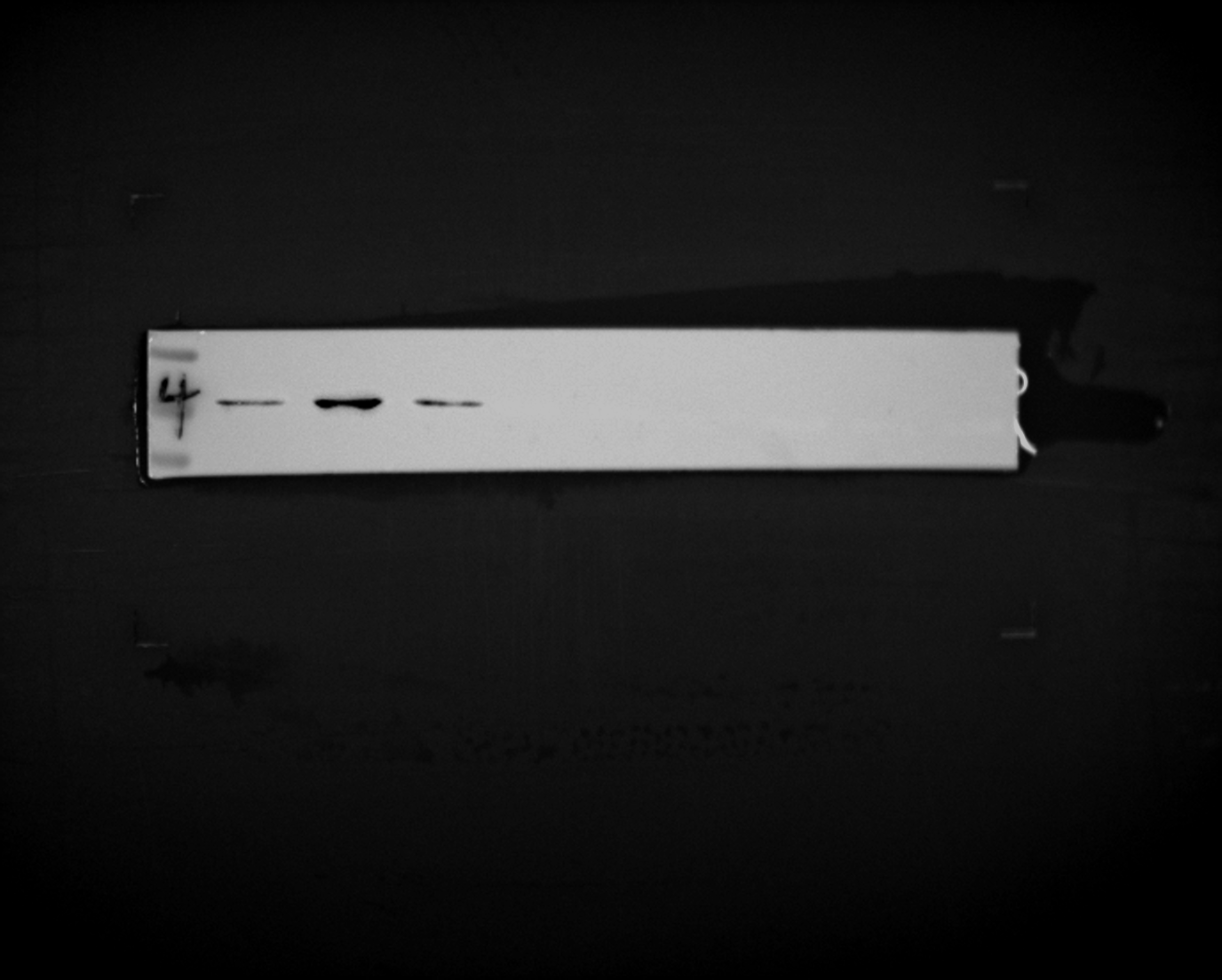

Supplement: Supplementary file 5 [file Data_Sheet_5.ZIP › Source data-Fig.5/Fig.5C/NF-a╩B(nuclear).tif]

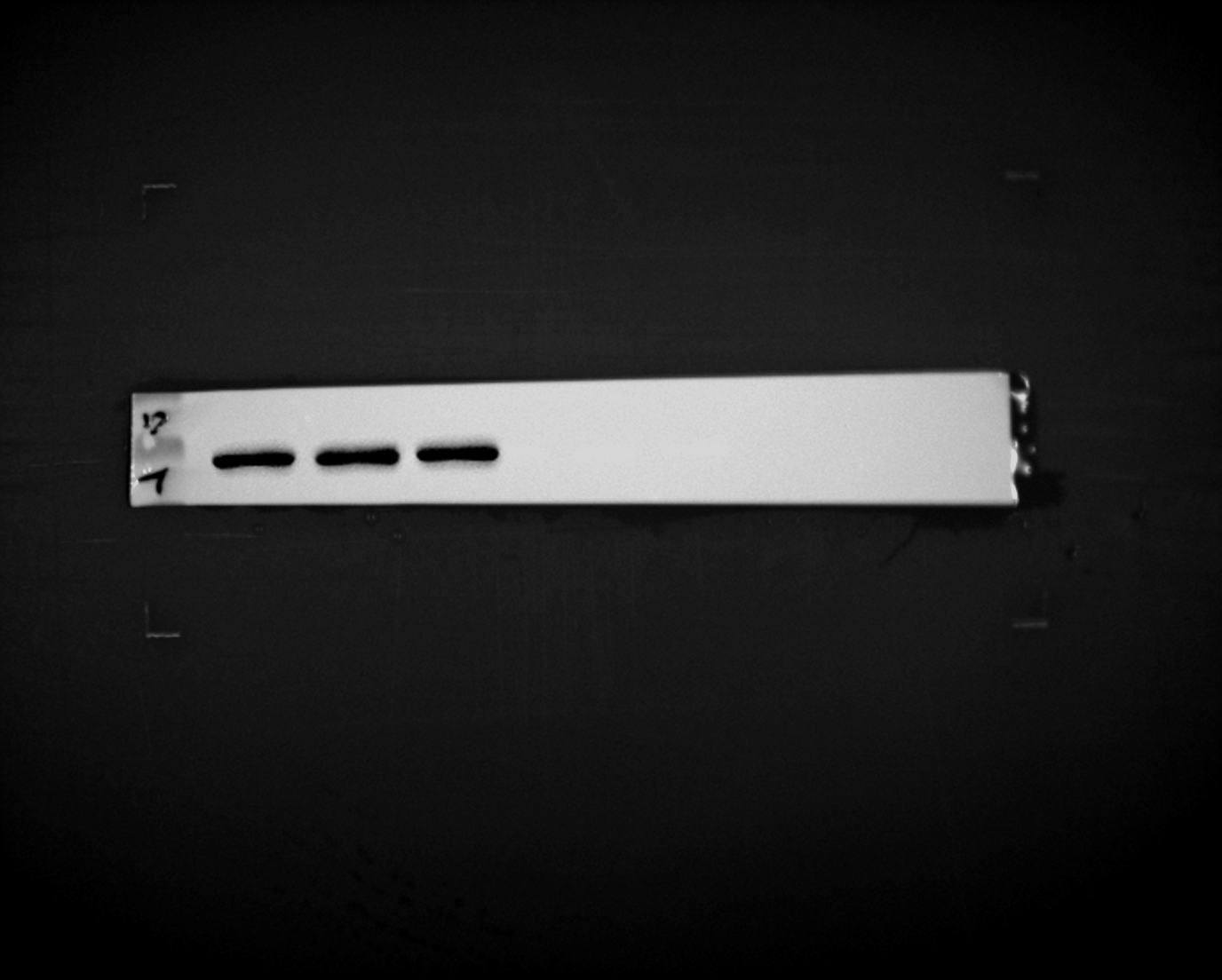

Supplement: Supplementary file 5 [file Data_Sheet_5.ZIP › Source data-Fig.5/Fig.5D/GAPDH.tif]

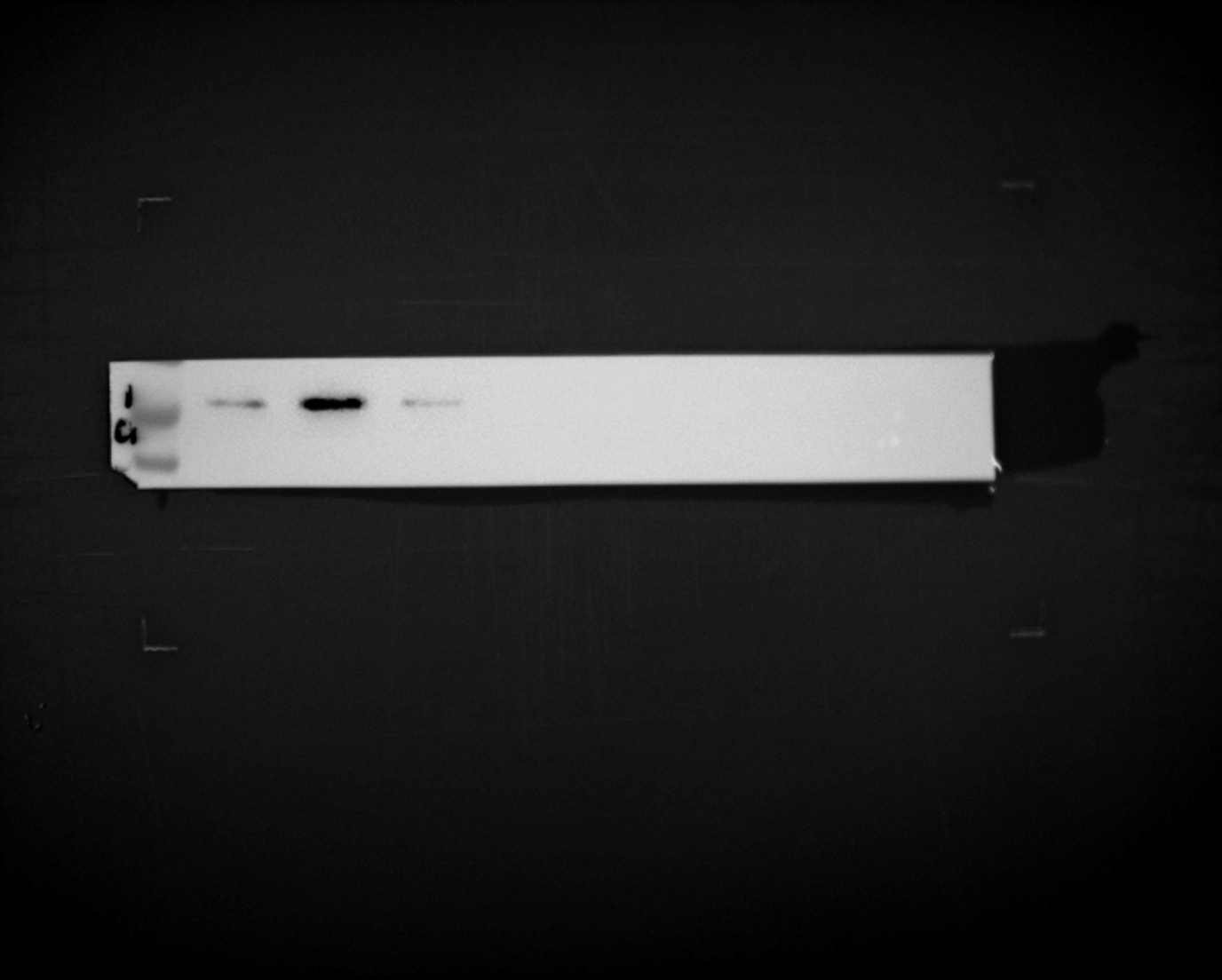

Supplement: Supplementary file 5 [file Data_Sheet_5.ZIP › Source data-Fig.5/Fig.5D/GSDMD-N.tif]

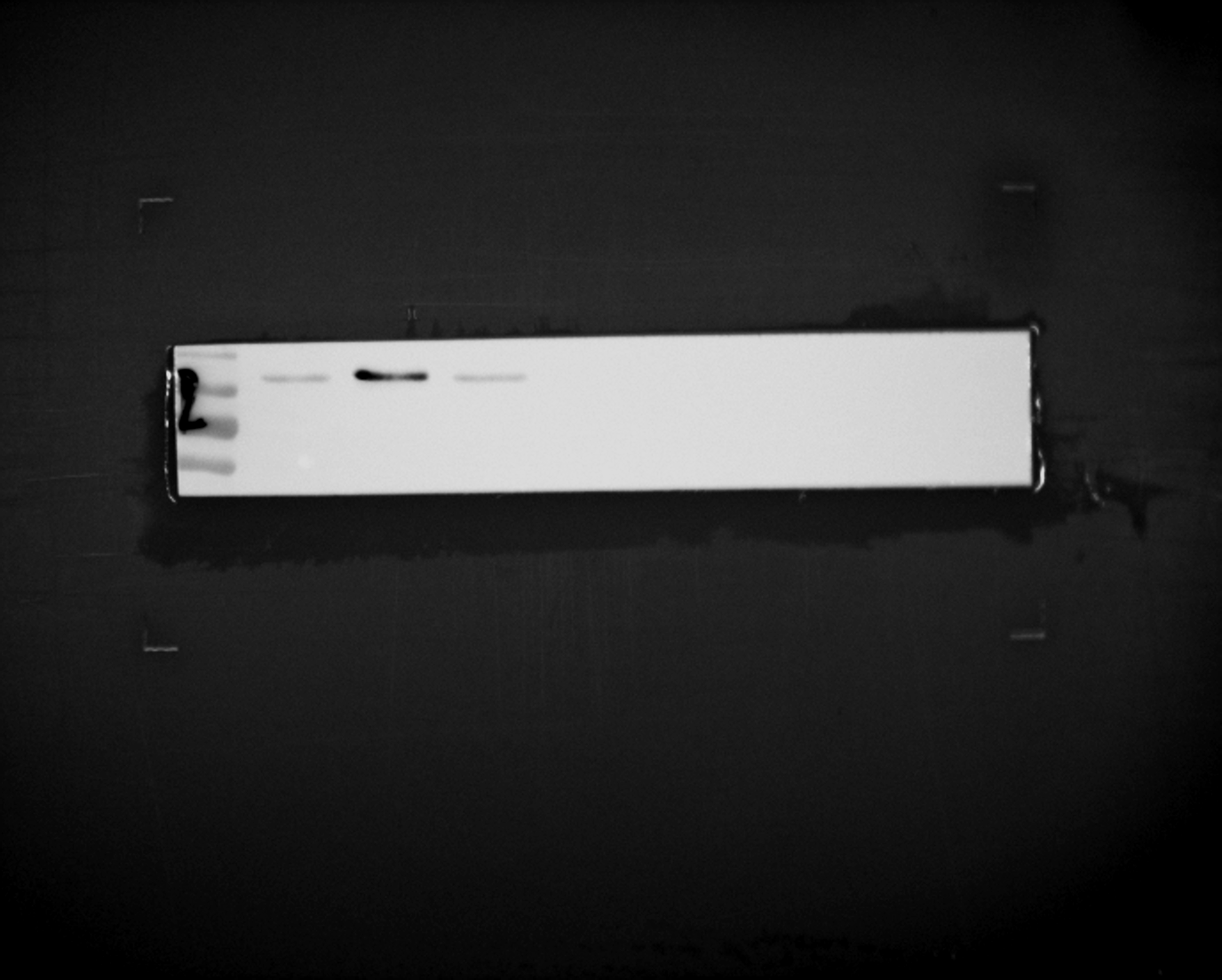

Supplement: Supplementary file 5 [file Data_Sheet_5.ZIP › Source data-Fig.5/Fig.5D/NLRP3.tif]

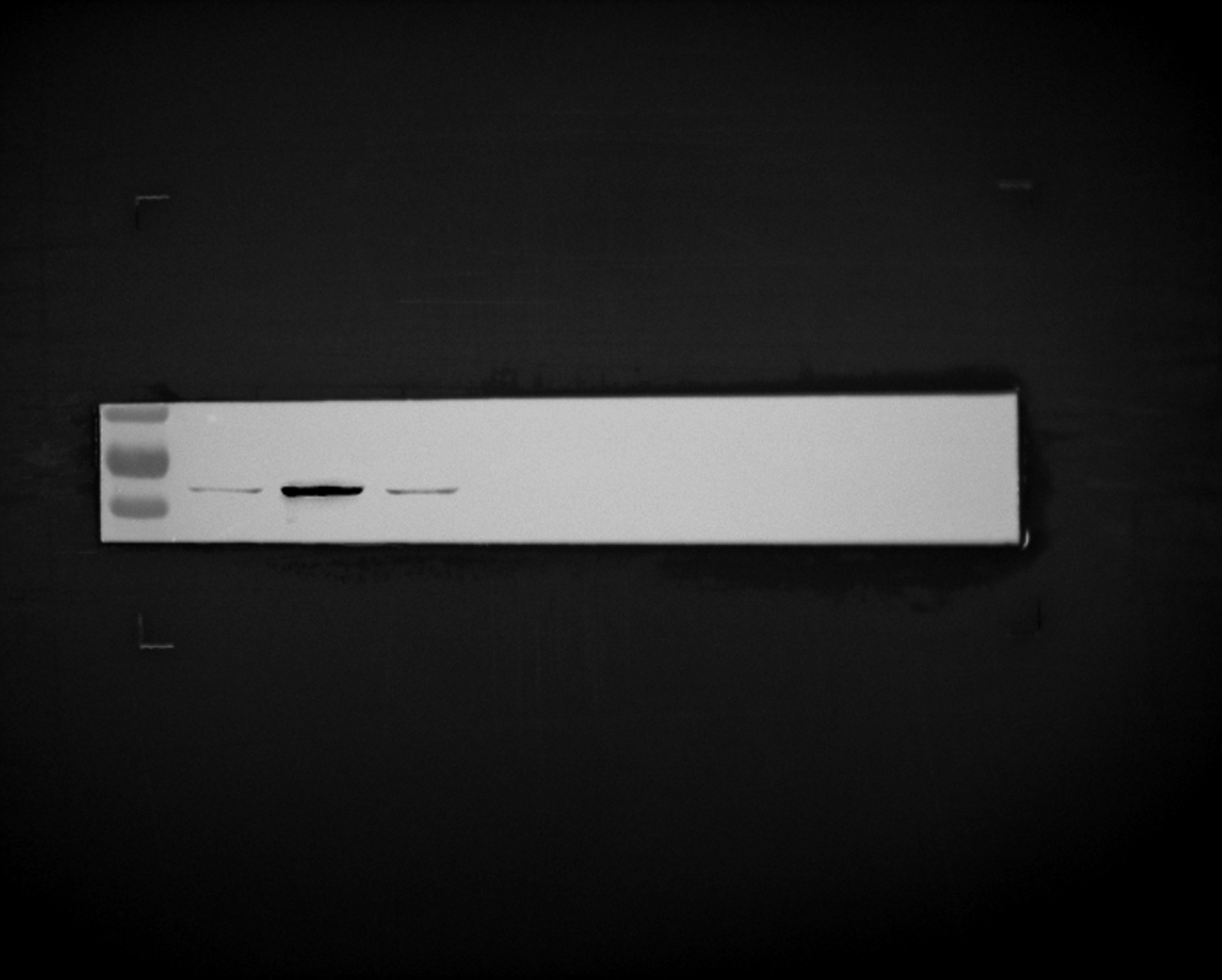

Supplement: Supplementary file 5 [file Data_Sheet_5.ZIP › Source data-Fig.5/Fig.5D/Pro-Caspase-1.tif]

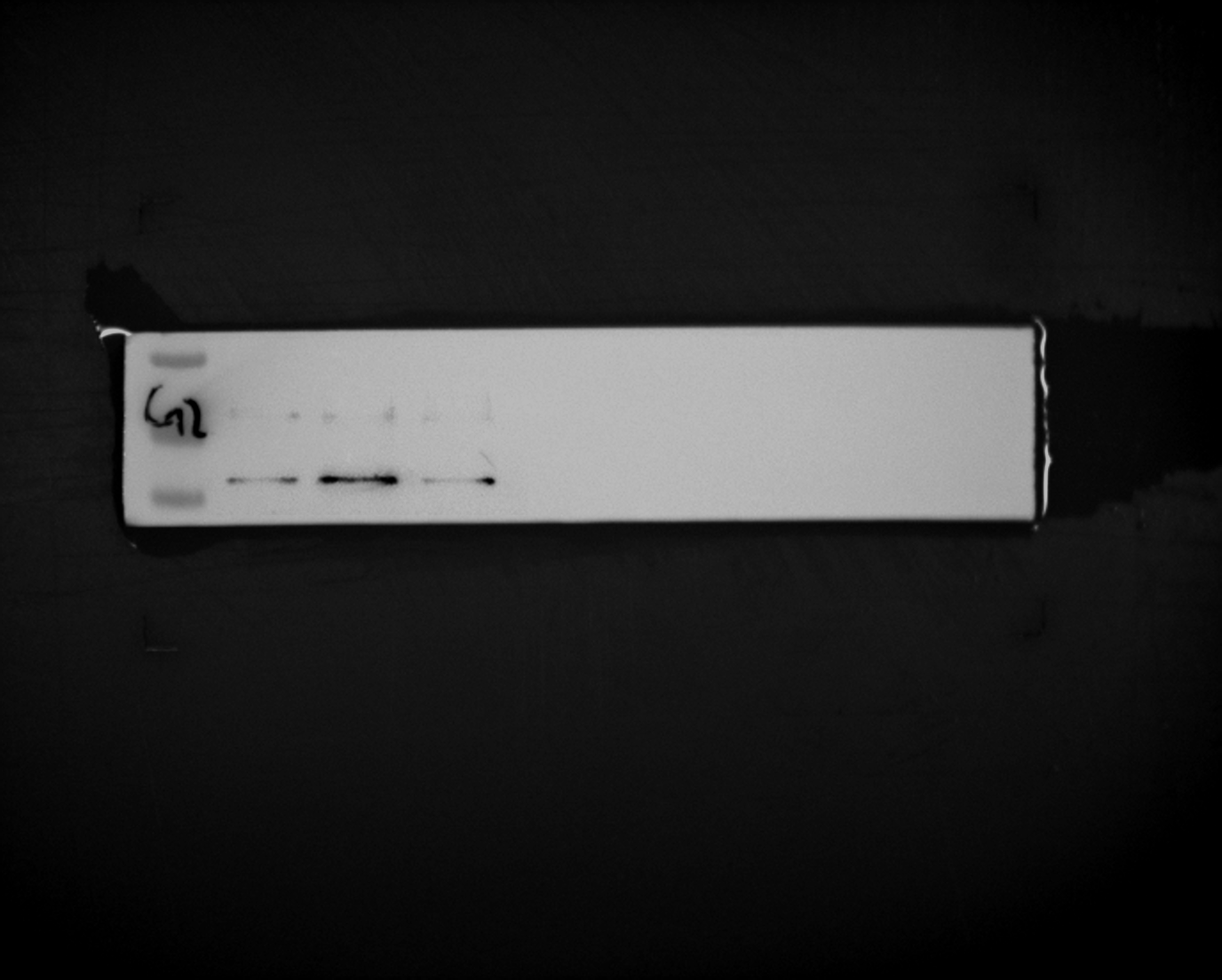

Supplement: Supplementary file 5 [file Data_Sheet_5.ZIP › Source data-Fig.5/Fig.5D/active Caspase-1.tif]

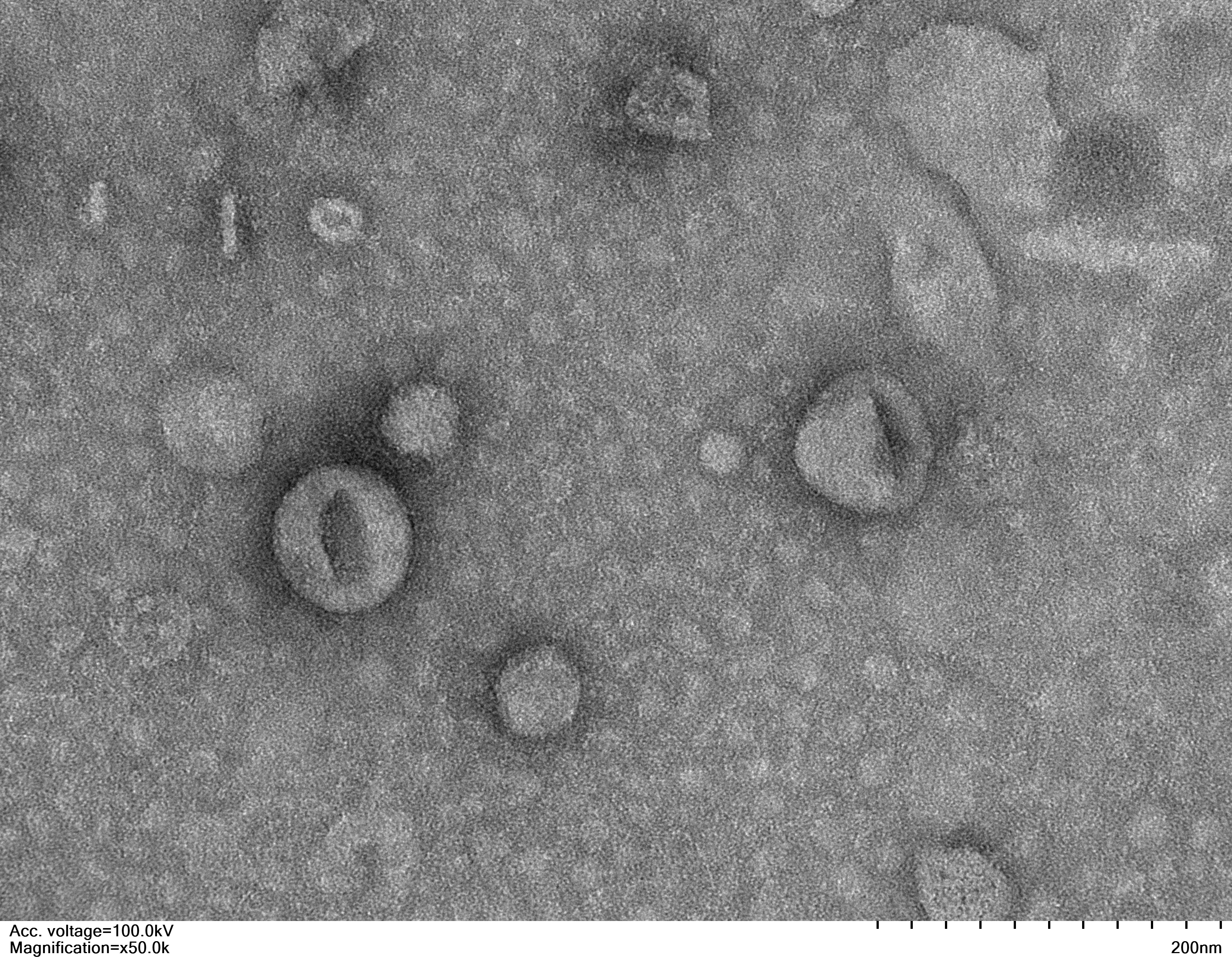

Supplement: Supplementary file 6 [file Data_Sheet_6.ZIP › Source data-Supp Fig.1A/control-exo.jpg]

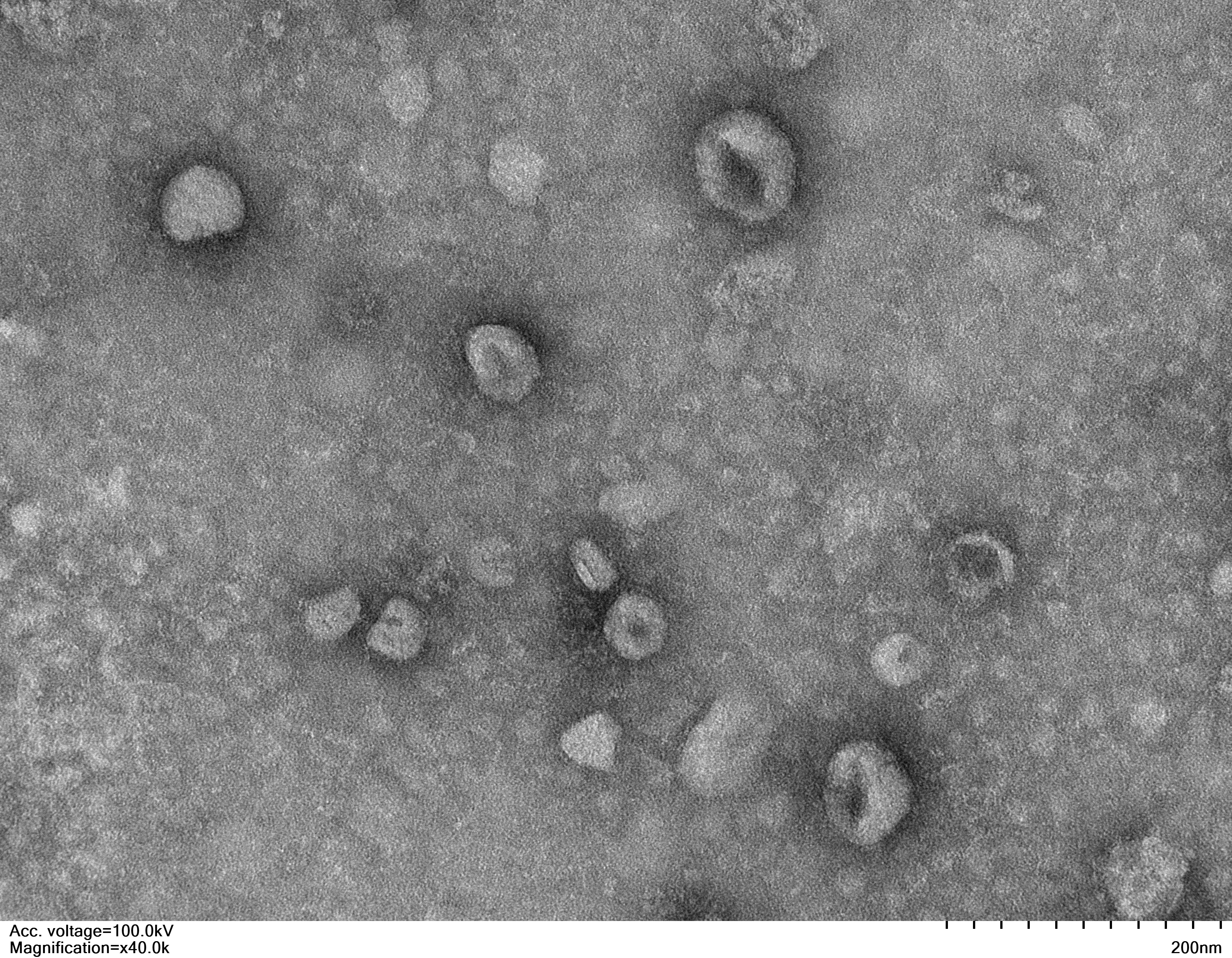

Supplement: Supplementary file 6 [file Data_Sheet_6.ZIP › Source data-Supp Fig.1A/sepsis-exo.jpg]

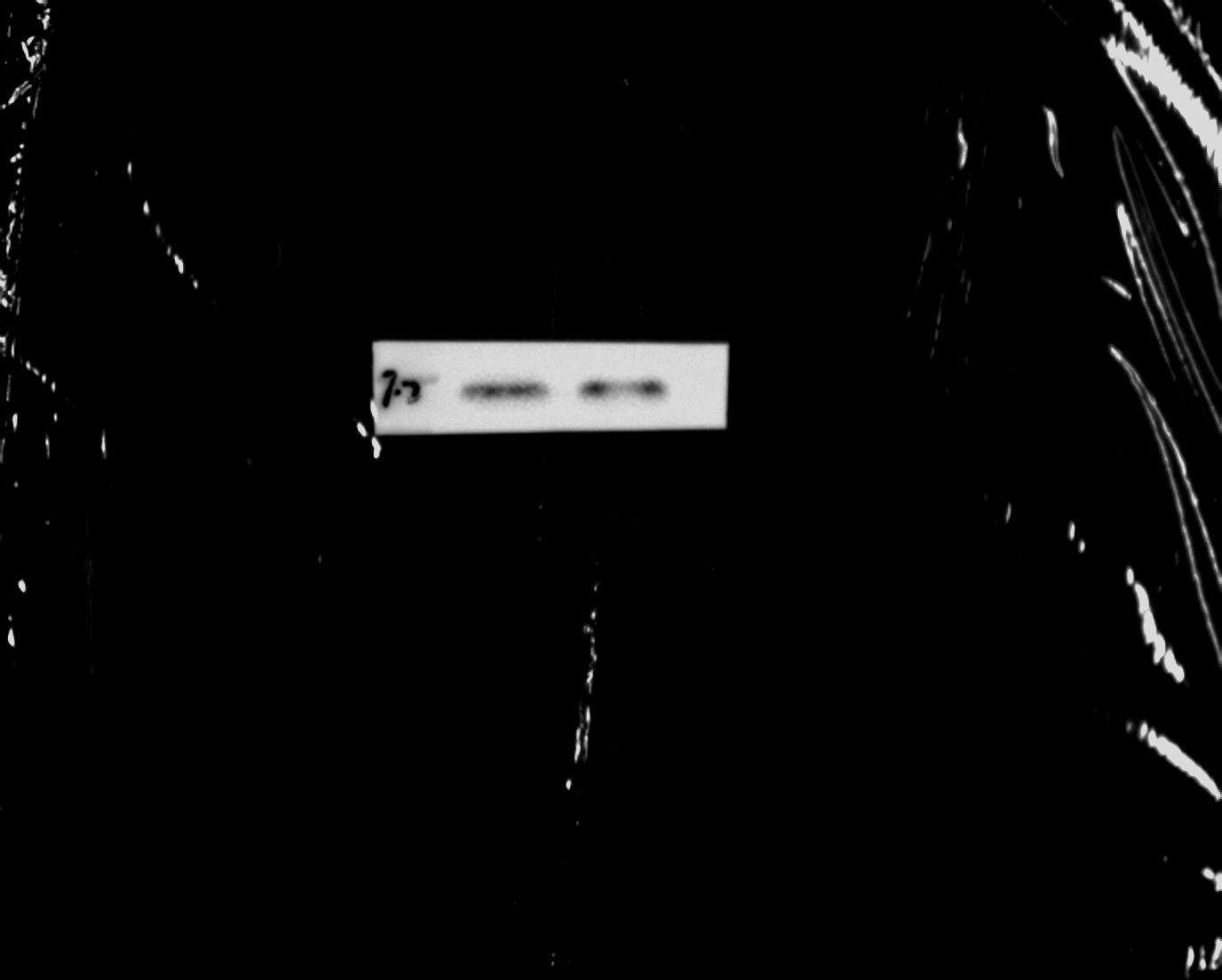

Supplement: Supplementary file 7 [file Data_Sheet_7.ZIP › Source data-Supp Fig.1B/CD63.tif]

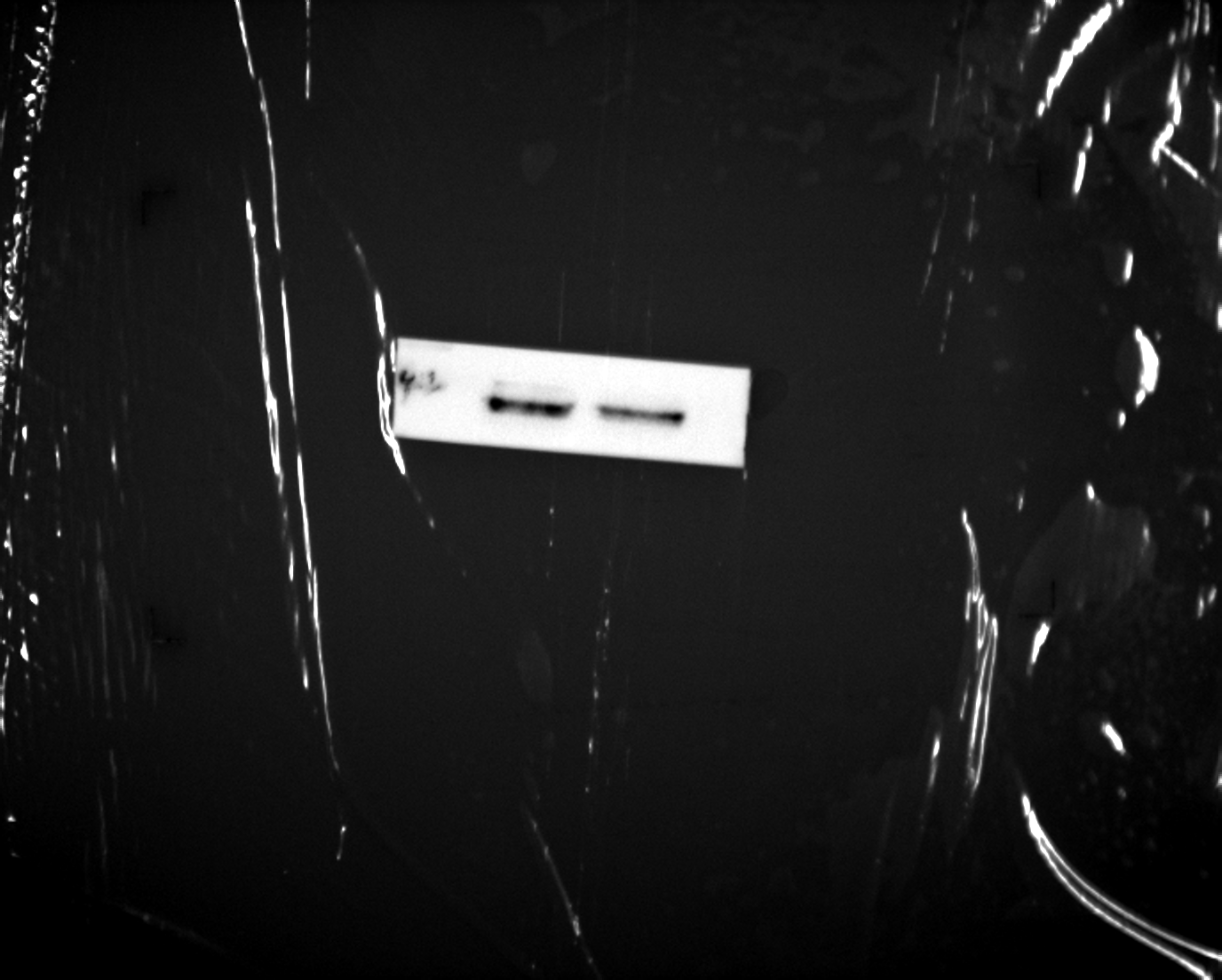

Supplement: Supplementary file 7 [file Data_Sheet_7.ZIP › Source data-Supp Fig.1B/CD81.tif]

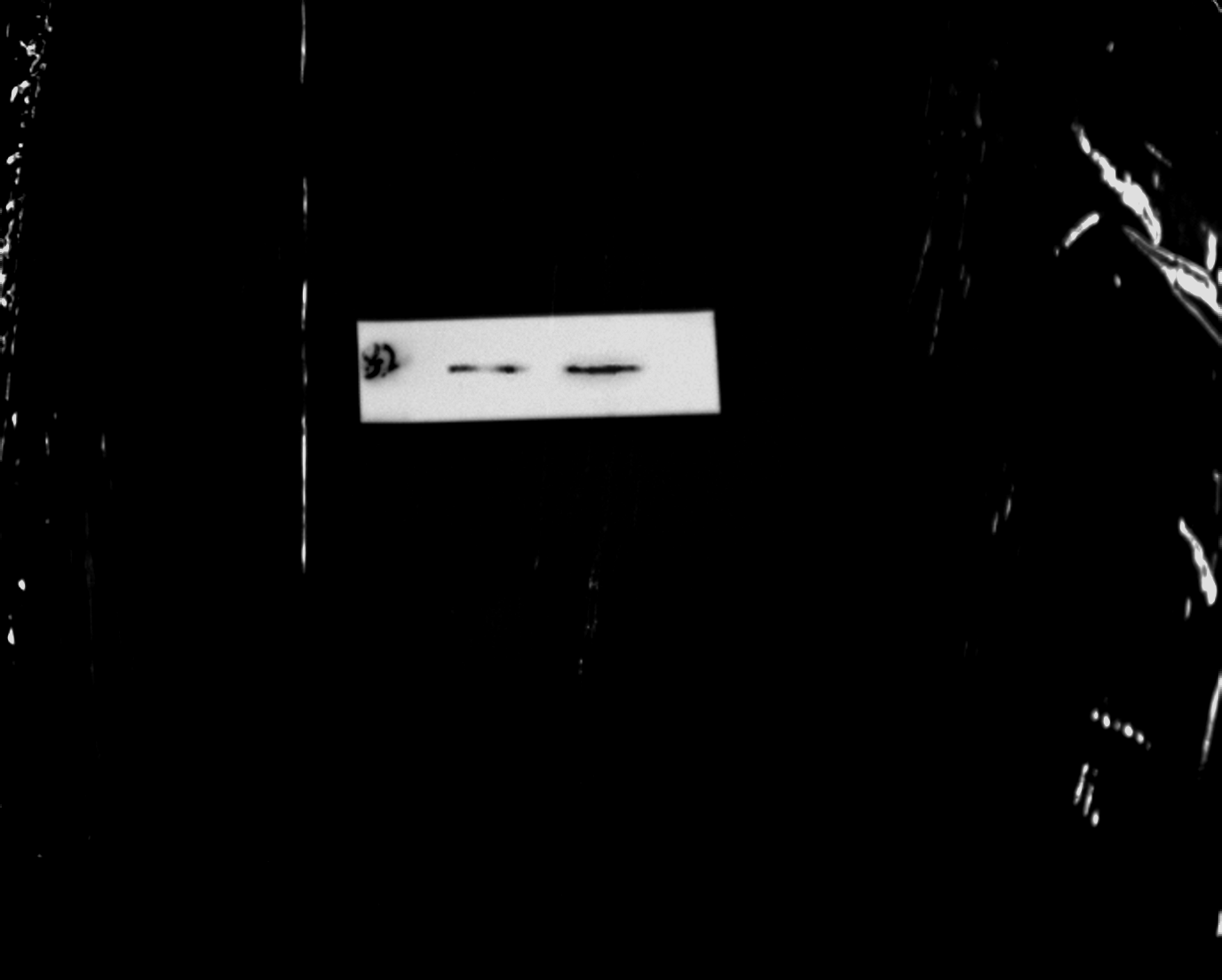

Supplement: Supplementary file 7 [file Data_Sheet_7.ZIP › Source data-Supp Fig.1B/CD9.tif]

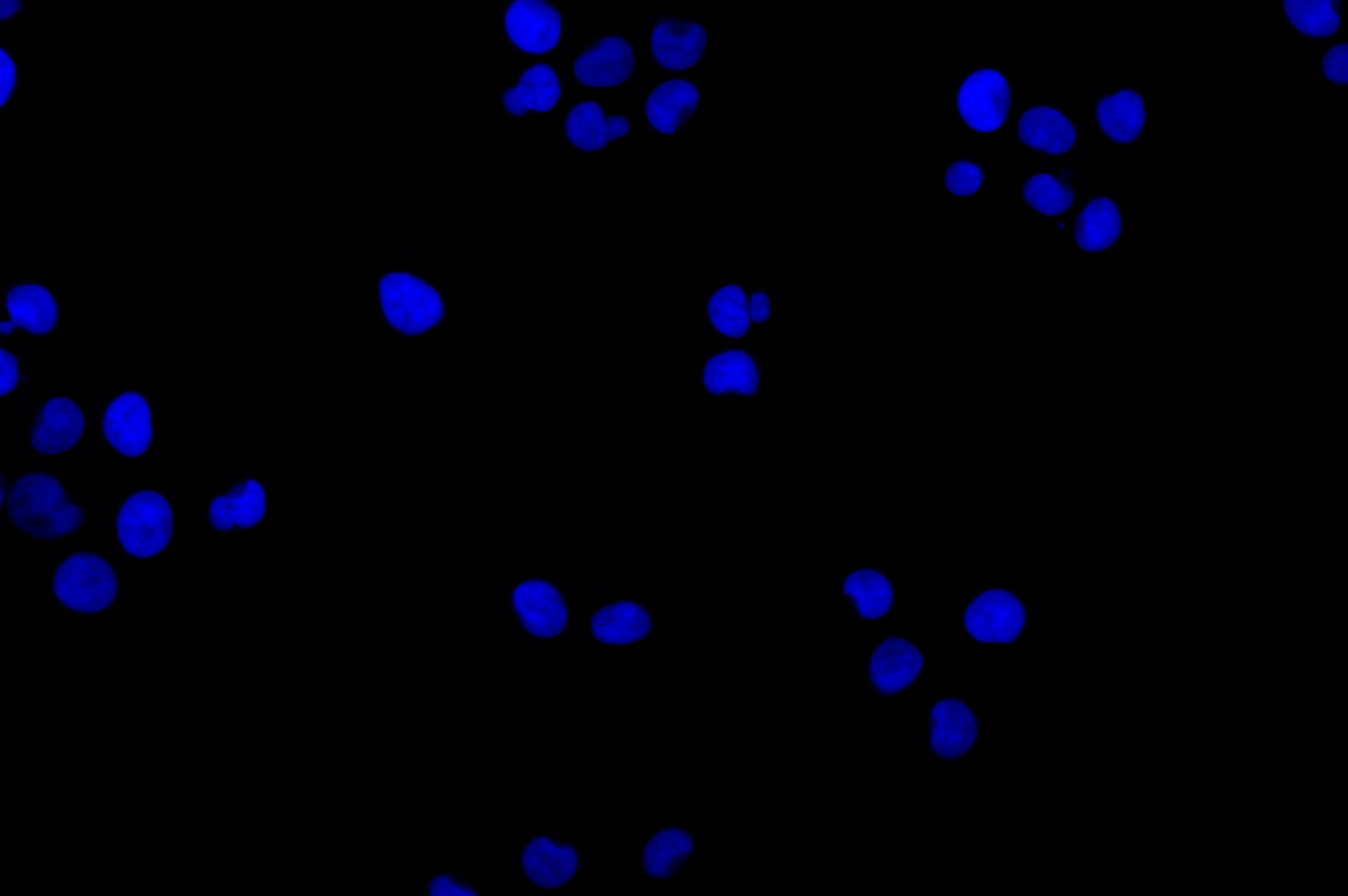

Supplement: Supplementary file 8 [file Data_Sheet_8.ZIP › Source data-Supp Fig.1C/control-DAPI.jpg]

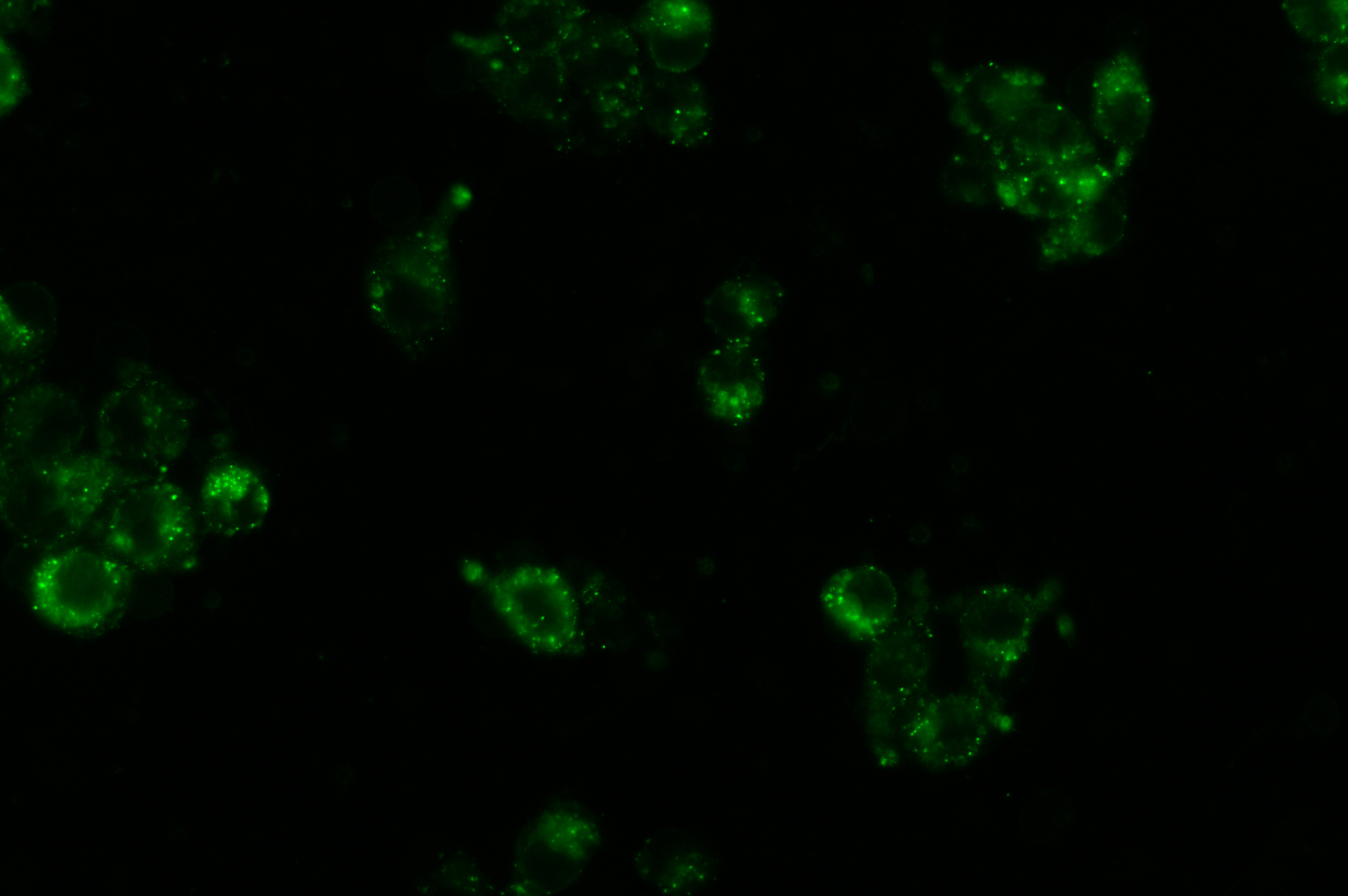

Supplement: Supplementary file 8 [file Data_Sheet_8.ZIP › Source data-Supp Fig.1C/control-PKH.jpg]

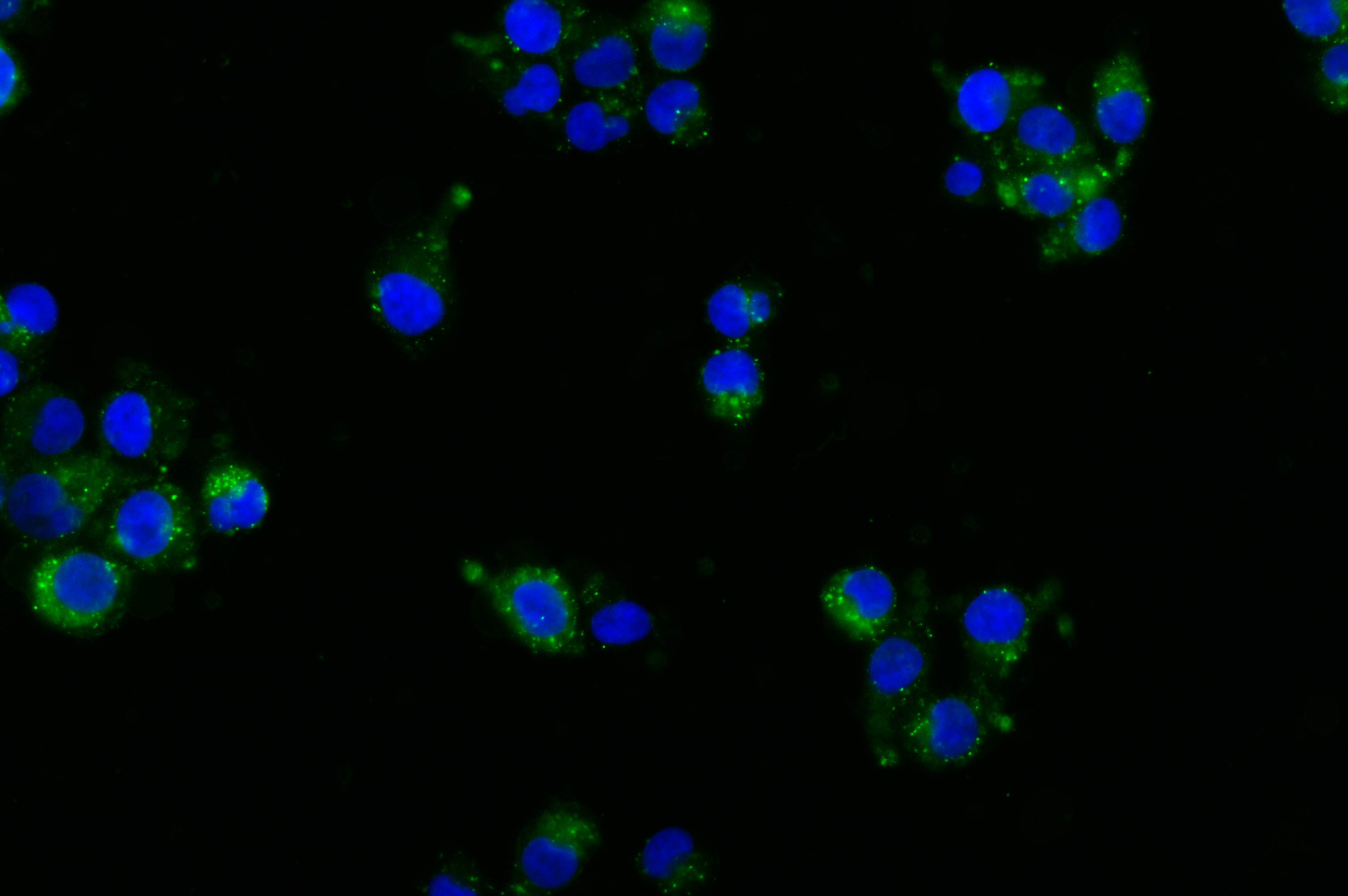

Supplement: Supplementary file 8 [file Data_Sheet_8.ZIP › Source data-Supp Fig.1C/control-merge.jpg]

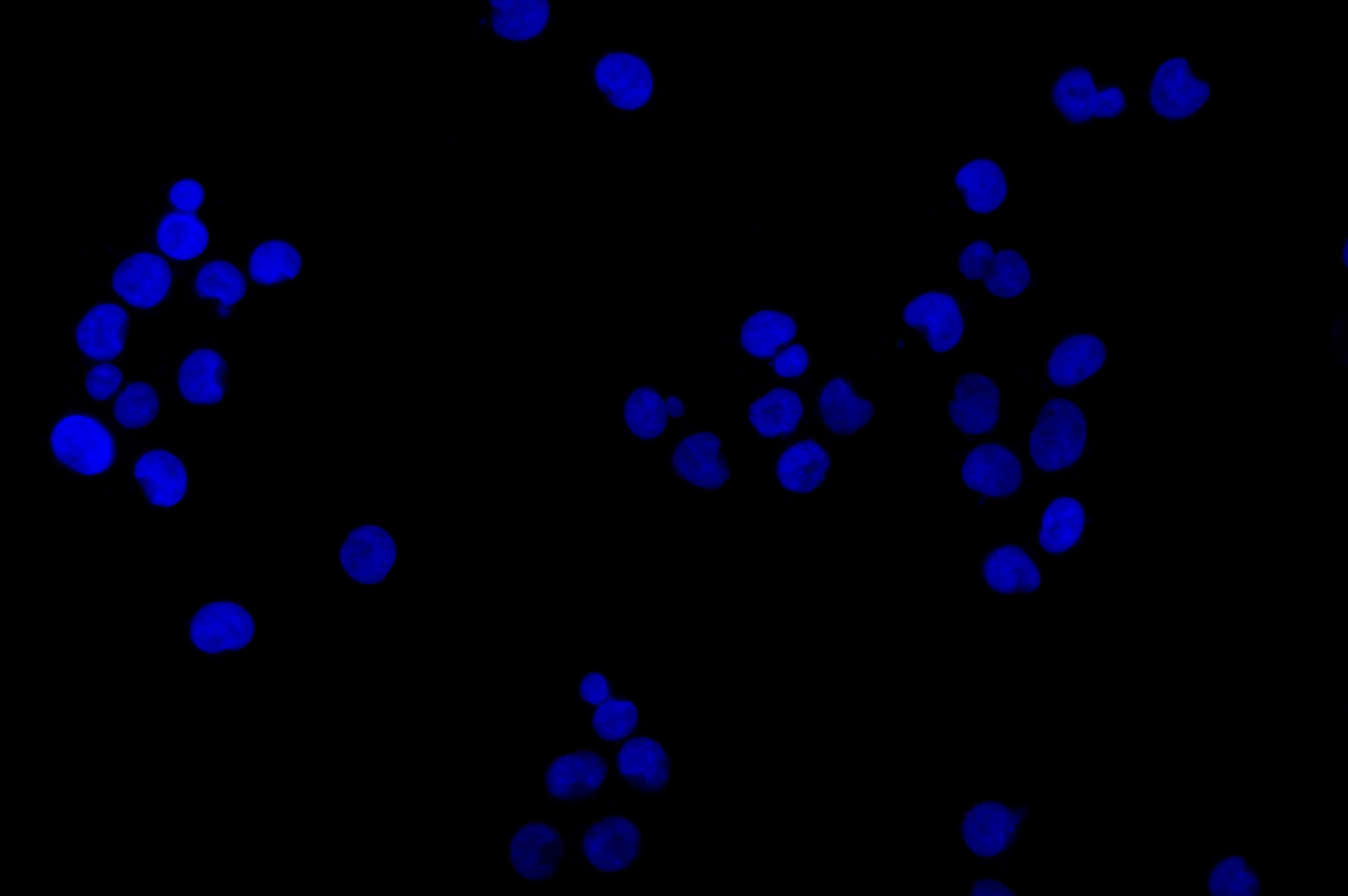

Supplement: Supplementary file 8 [file Data_Sheet_8.ZIP › Source data-Supp Fig.1C/sepsis-DAPI.jpg]

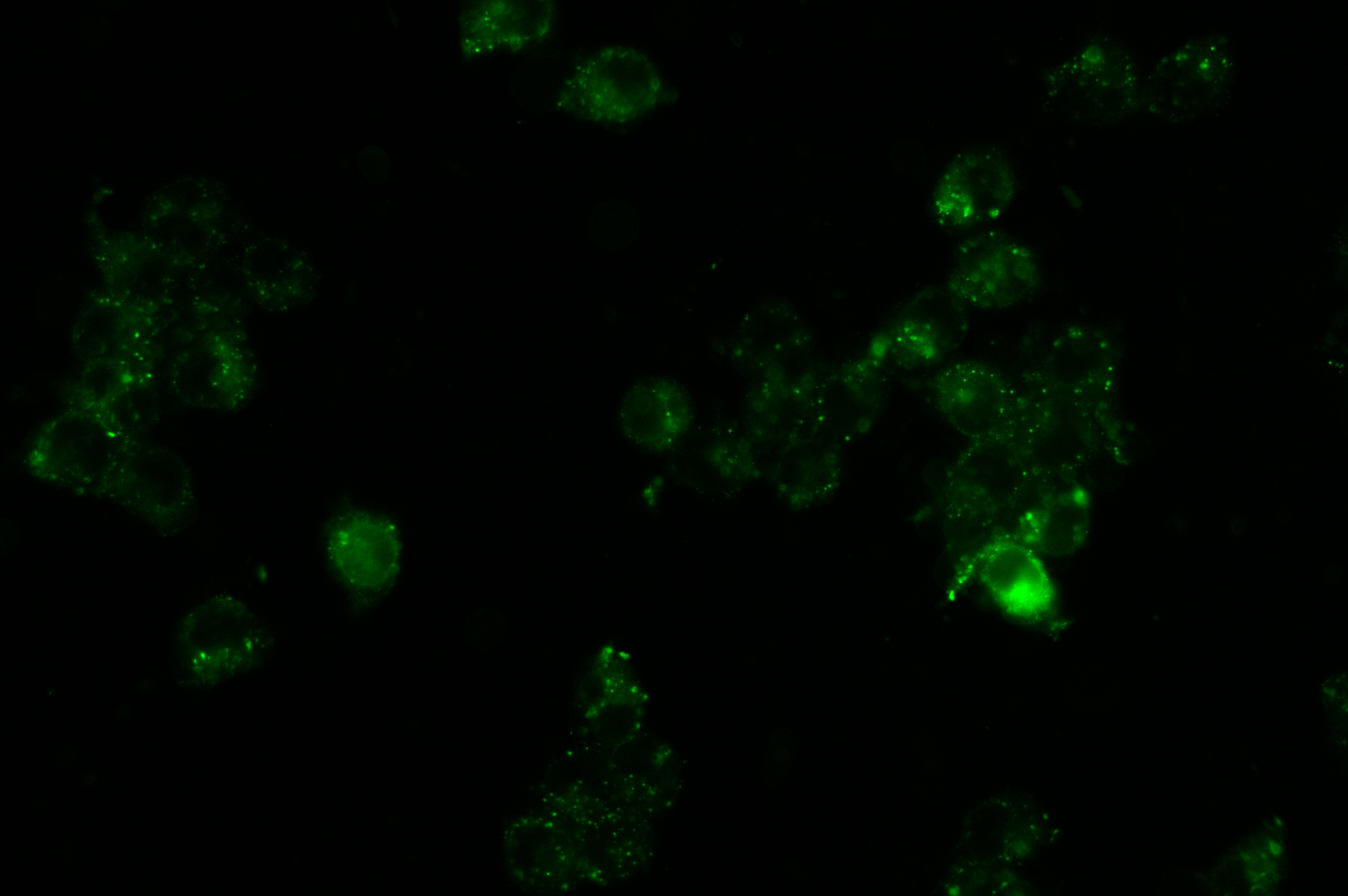

Supplement: Supplementary file 8 [file Data_Sheet_8.ZIP › Source data-Supp Fig.1C/sepsis-PKH.jpg]

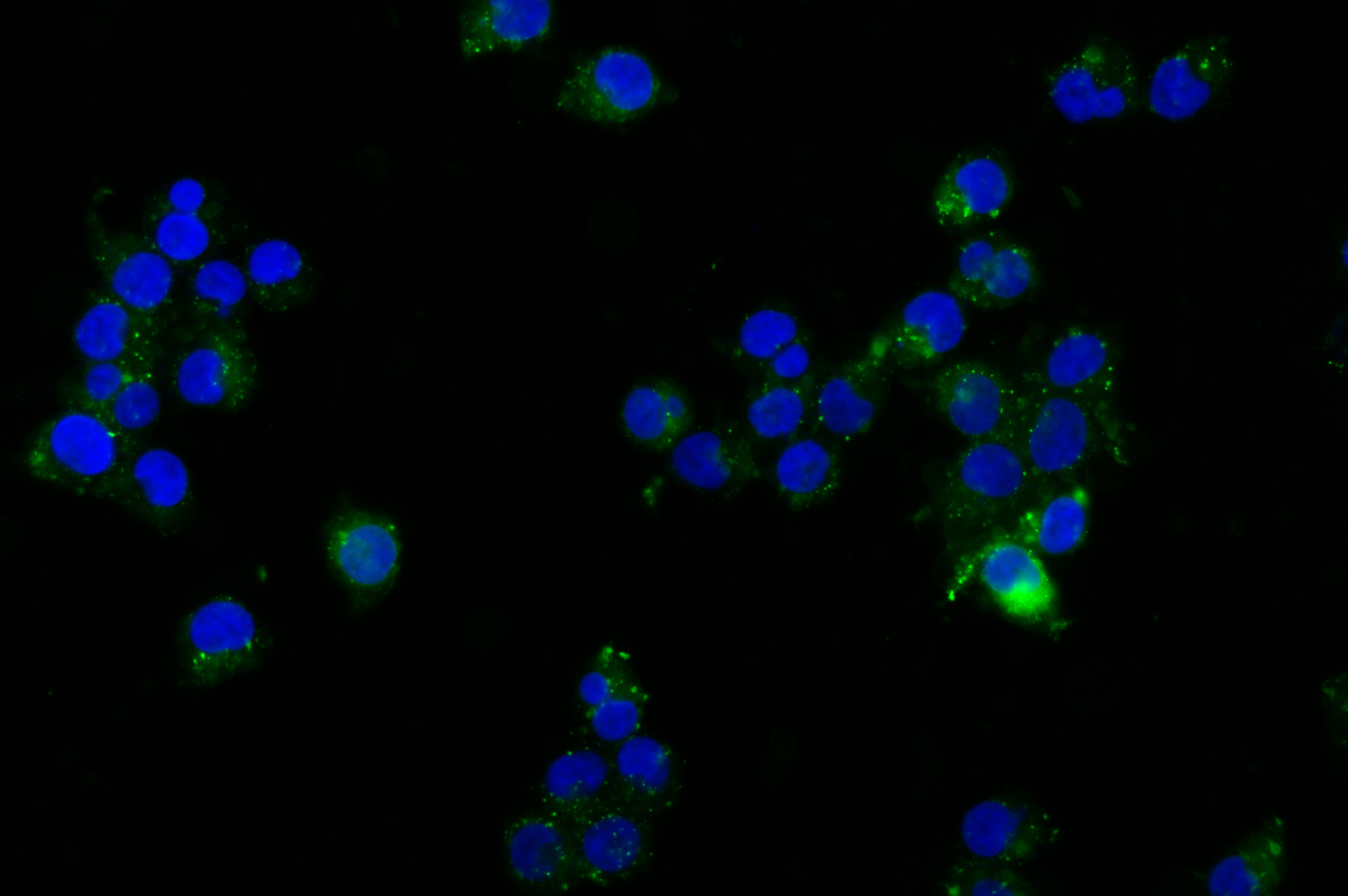

Supplement: Supplementary file 8 [file Data_Sheet_8.ZIP › Source data-Supp Fig.1C/sepsis-merge.jpg]

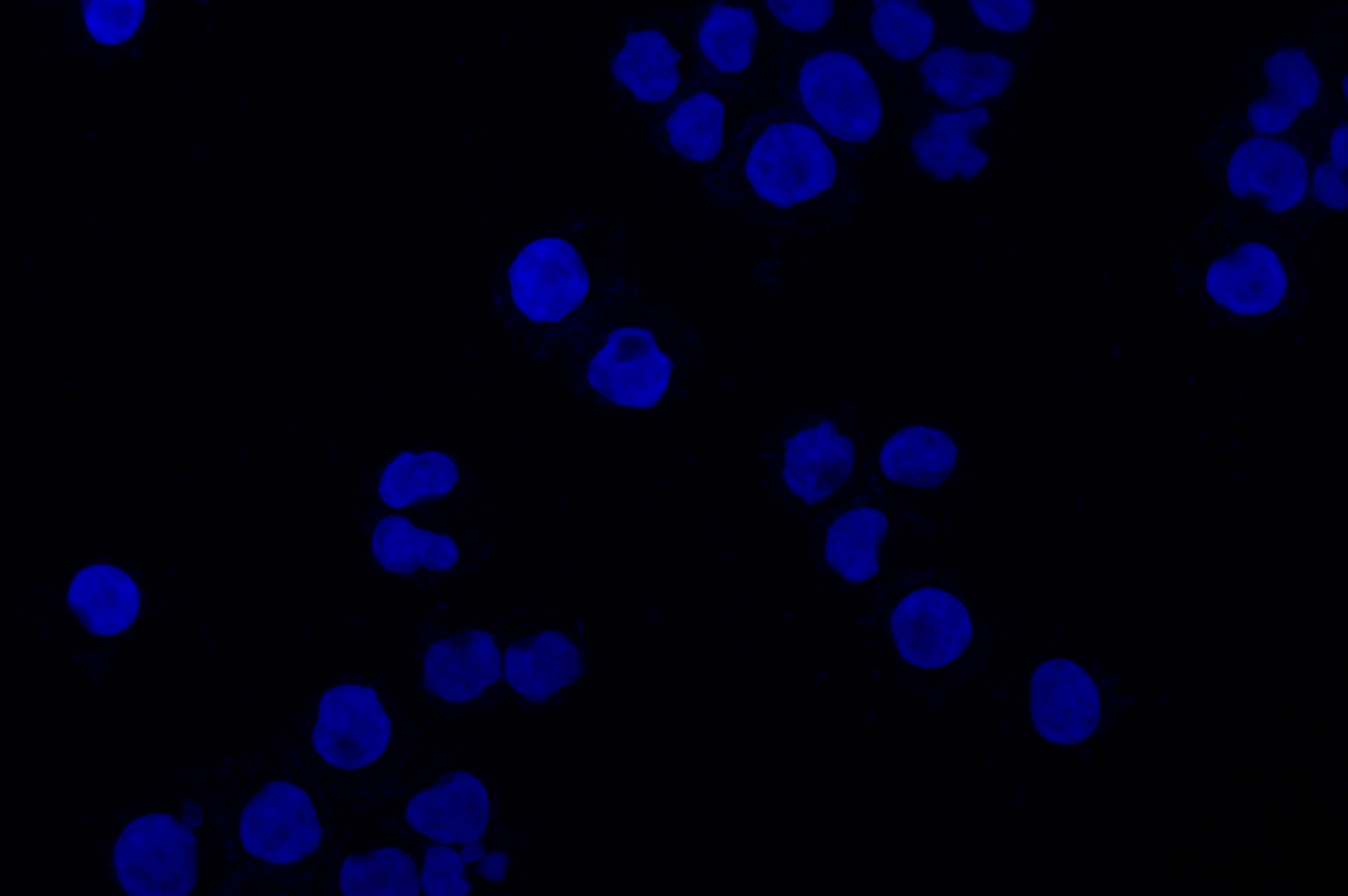

Supplement: Supplementary file 8 [file Data_Sheet_8.ZIP › Source data-Supp Fig.1C/vehicle-DAPI.jpg]

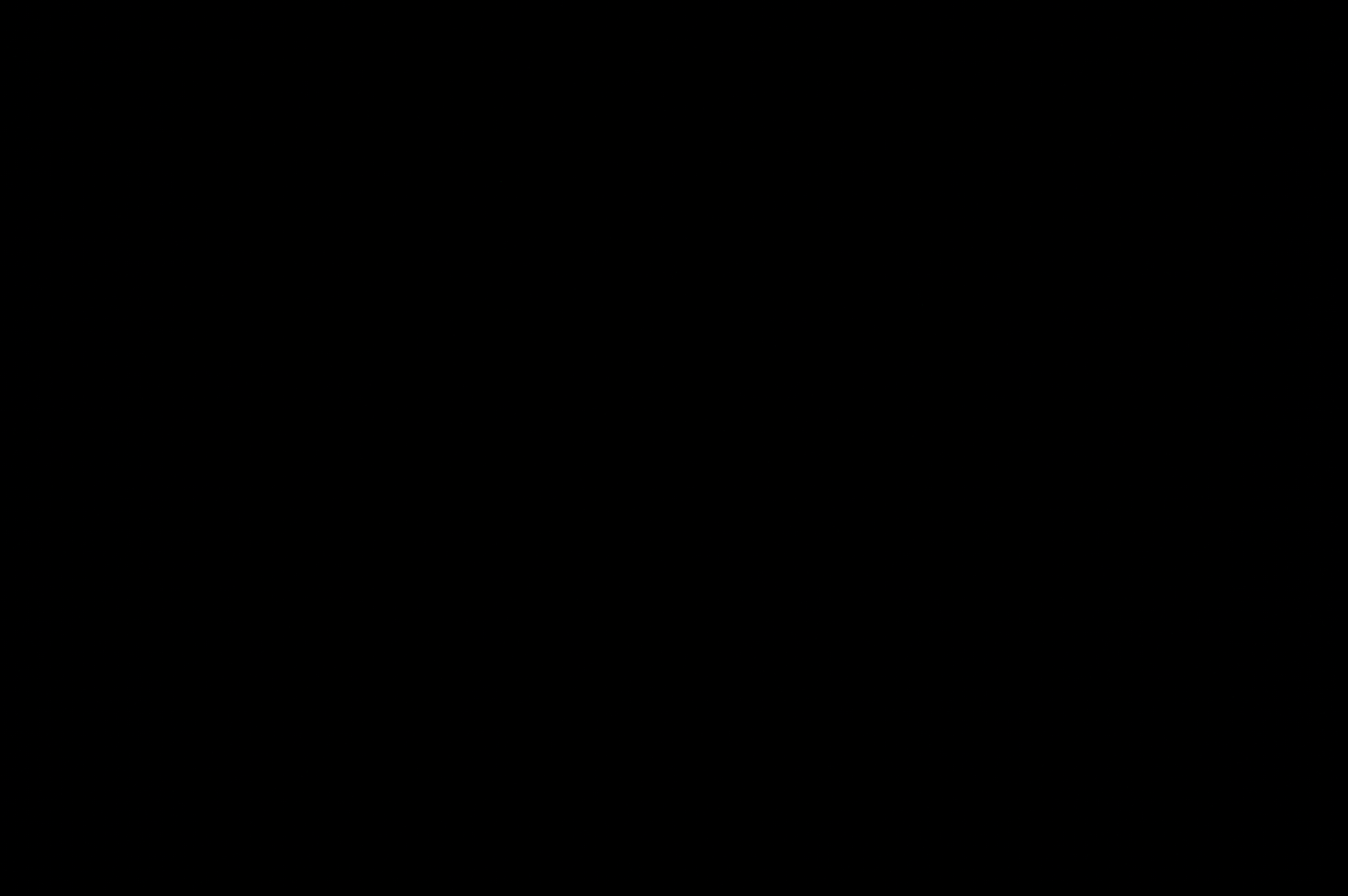

Supplement: Supplementary file 8 [file Data_Sheet_8.ZIP › Source data-Supp Fig.1C/vehicle-PKH.jpg]

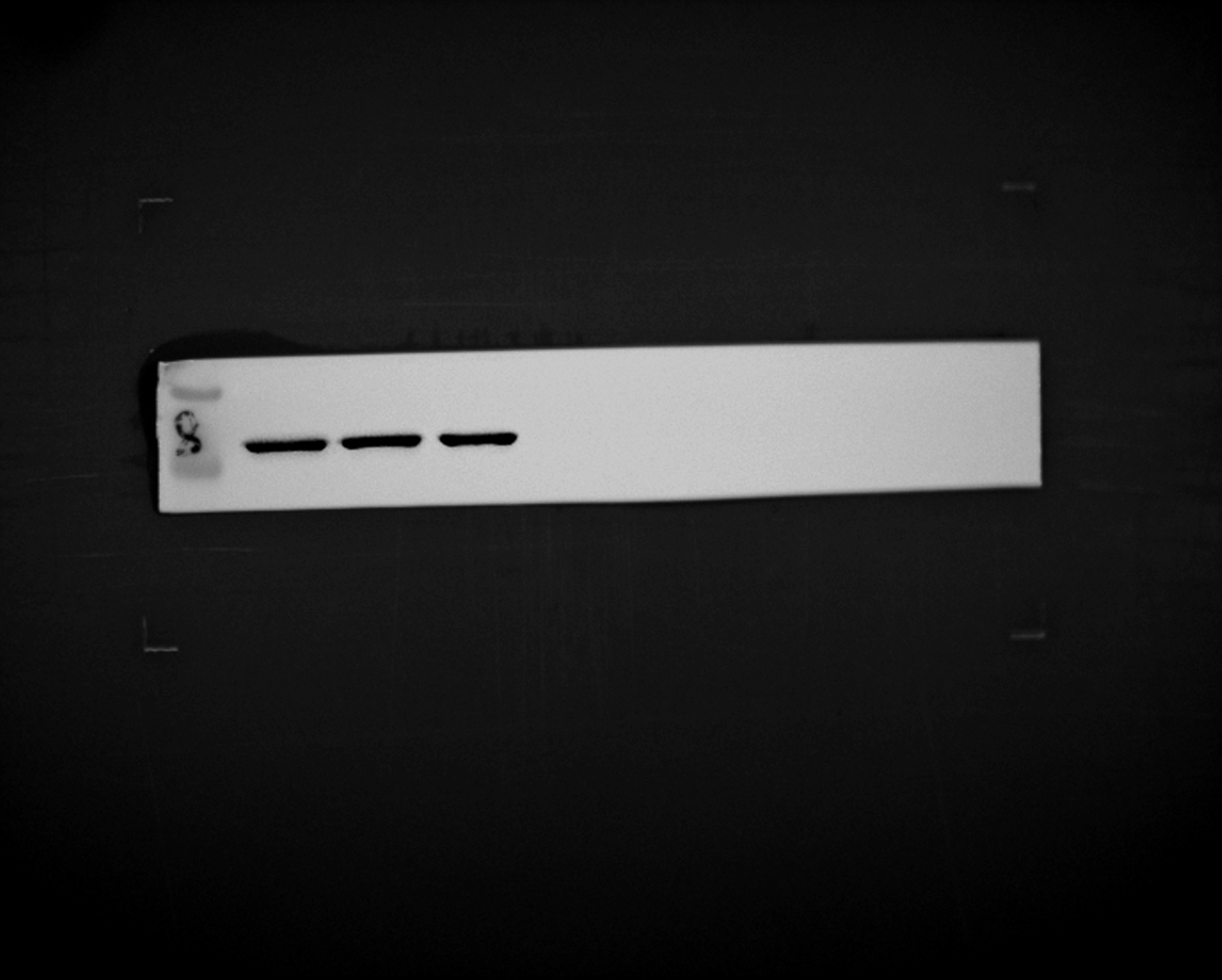

Supplement: Supplementary file 9 [file Data_Sheet_9.ZIP › Source data-Supp Fig.2/Supp Fig.2B/GAPDH.tif]

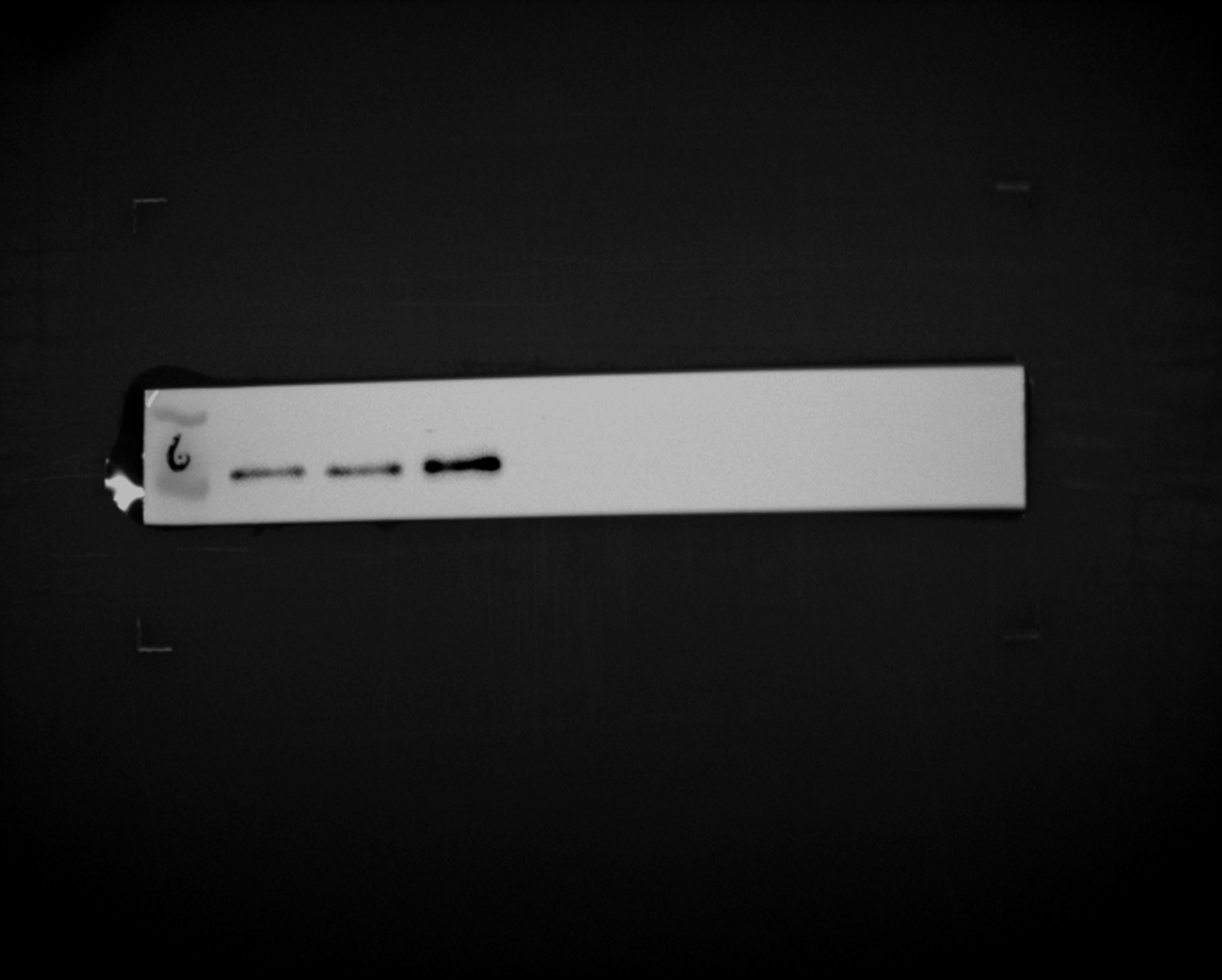

Supplement: Supplementary file 9 [file Data_Sheet_9.ZIP › Source data-Supp Fig.2/Supp Fig.2B/HMBOX1.tif]

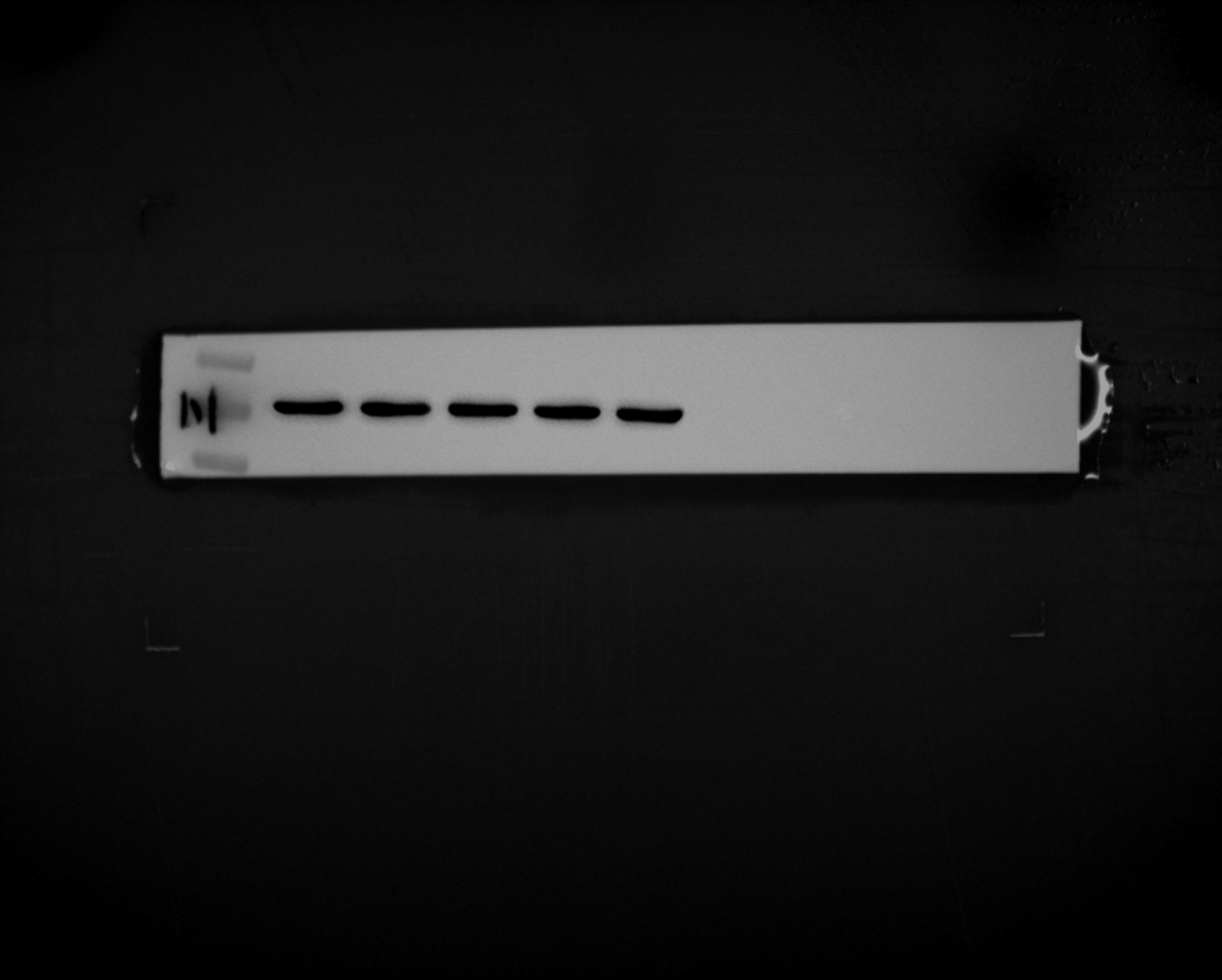

Supplement: Supplementary file 9 [file Data_Sheet_9.ZIP › Source data-Supp Fig.2/Supp Fig.2D/GAPDH.tif]

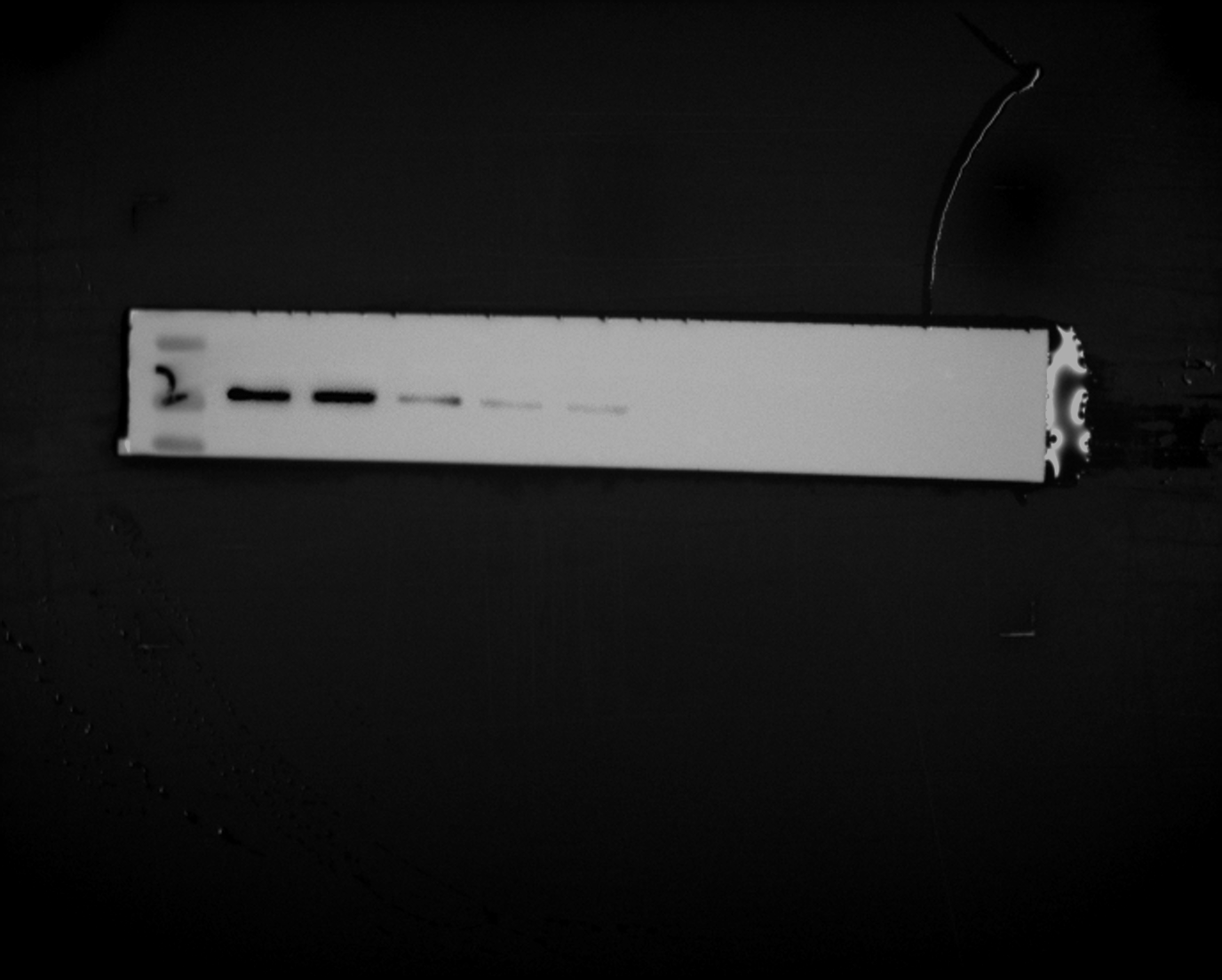

Supplement: Supplementary file 9 [file Data_Sheet_9.ZIP › Source data-Supp Fig.2/Supp Fig.2D/HMBOX1.tif]

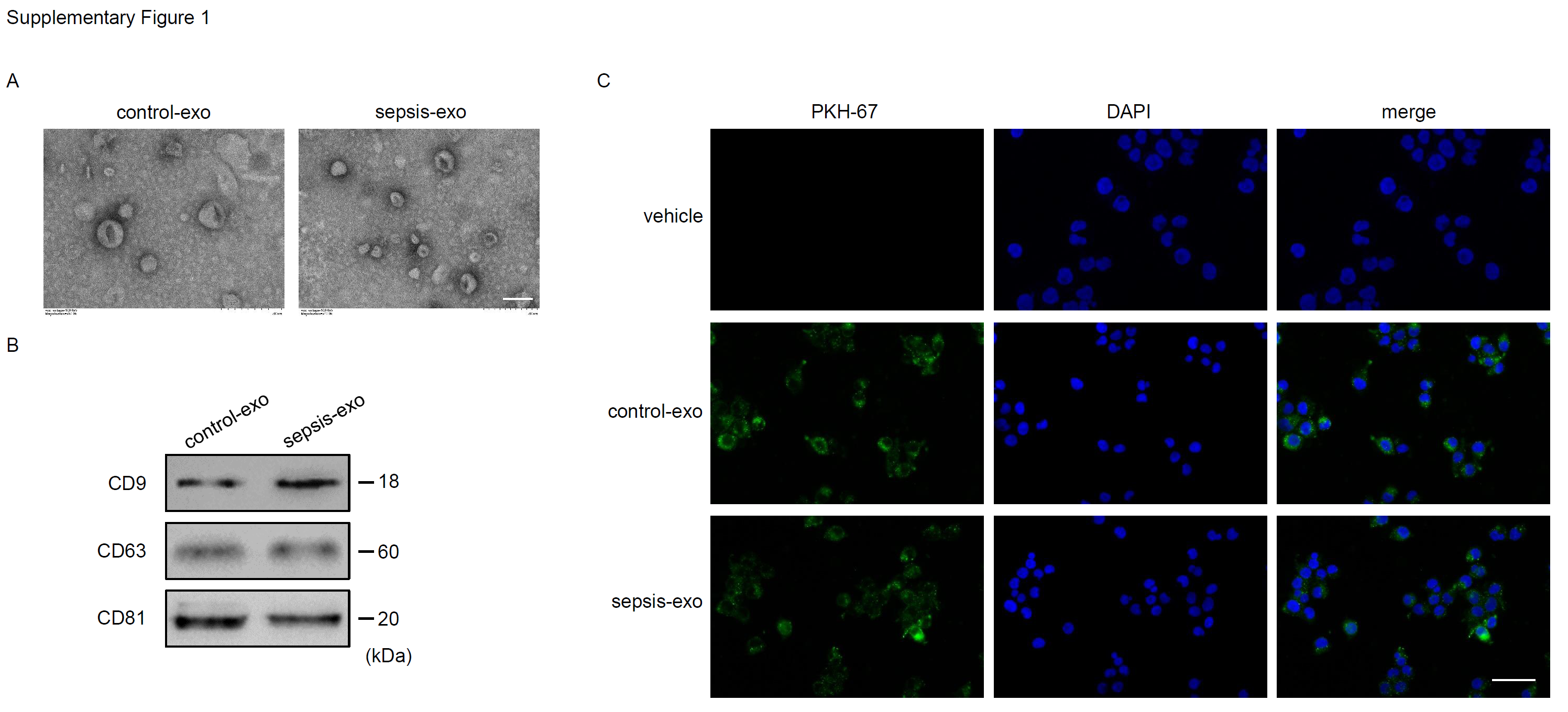

Supplement: Supplementary Figure 1 — Characterization of exosomes. (A) Sepsis-exos and control-exos were subjected to TEM observation. Scale bar, 1 μm. (B) The protein levels exosome markers CD9, CD63, and CD81 were measured by western blot. (C) The exosome endocytosis assay was observed by a fluorescent microscope. Scale bar, 25 μm. (A–C) Data are from three independent experiments. * p < 0.05. [file Image_1.TIF]

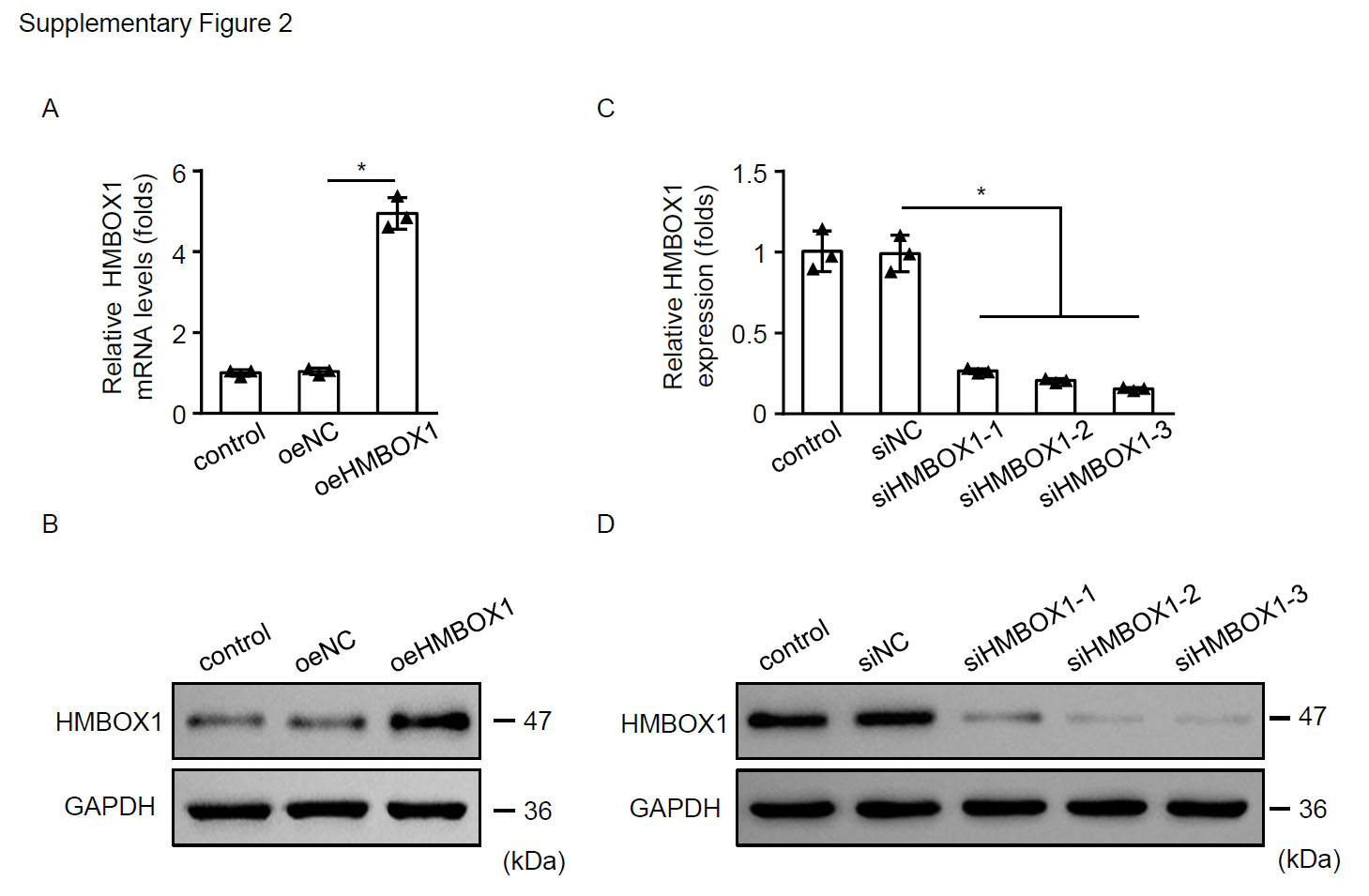

Supplement: Supplementary Figure 2 — Overexpression and knockdown of HMBOX1 in AC cells. (A,B) HMBOX1 was overexpressed in AC16 cells. (A) mRNA expression of HMBOX1 in cells was measured by qPCR. Data are normalized to expression levels of control. (B) The protein level of HMBOX1 in cells were measured by western blot. (C,D) HMBOX1 was silenced in AC cells with 3 siRNAs, respectively. (C) mRNA expression of HMBOX1 in cells was measured by qPCR. Data are normalized to expression levels of control. (D) The protein level of HMBOX1 in cells were measured by western blot. (A–C) Data are mean ± SD from three independent experiments (n = 3 per group). * p < 0.05. [file Image_2.JPEG]
